# Natural Products Repertoire of the Red Sea

**DOI:** 10.3390/md18090457

**Published:** 2020-09-04

**Authors:** Ebaa M. El-Hossary, Mohammad Abdel-Halim, Eslam S. Ibrahim, Sheila Marie Pimentel-Elardo, Justin R. Nodwell, Heba Handoussa, Miada F. Abdelwahab, Ulrike Holzgrabe, Usama Ramadan Abdelmohsen

**Affiliations:** 1 National Centre for Radiation Research & Technology, Egyptian Atomic Energy Authority, Ahmed El-Zomor St. 3, El-Zohoor Dist., Nasr City, Cairo 11765, Egypt; ebaa.elhossary@eaea.org.eg; 2 Department of Pharmaceutical Chemistry, Faculty of Pharmacy and Biotechnology, German University in Cairo, Cairo 11835, Egypt; mohammad.abdel-halim@guc.edu.eg; 3 Department of Microbiology and Immunology, Faculty of Pharmacy, Cairo University, Cairo 11562, Egypt; eslam.ebrahim@pharma.cu.edu.eg; 4 Institute for Molecular Infection Biology, University of Würzburg, Josef-Schneider-Strasse 2/Bau D15, 97080 Würzburg, Germany; 5 Department of Biochemistry, University of Toronto, MaRS Centre West, 661 University Avenue, Toronto, ON M5G 1M1, Canada; sheila.elardo@utoronto.ca (S.M.P.-E.); justin.nodwell@utoronto.ca (J.R.N.); 6 Department of Pharmaceutical Biology, Faculty of Pharmacy and Biotechnology, German University in Cairo, Cairo 11835, Egypt; heba.handoussa@guc.edu.eg; 7 Department of Pharmacognosy, Faculty of Pharmacy, Minia University, Minia 61519, Egypt; mayada.mohamed2@mu.edu.eg; 8 Institute for Pharmacy and Food Chemistry, University of Würzburg, Am Hubland, 97074 Würzburg, Germany; 9 Department of Pharmacognosy, Faculty of Pharmacy, Deraya University, Universities Zone, P.O. Box 61111 New Minia City, Minia 61519, Egypt

**Keywords:** Red Sea, marine natural products, marine organisms, biodiversity, marine metagenomics, bioactivity

## Abstract

Marine natural products have achieved great success as an important source of new lead compounds for drug discovery. The Red Sea provides enormous diversity on the biological scale in all domains of life including micro- and macro-organisms. In this review, which covers the literature to the end of 2019, we summarize the diversity of bioactive secondary metabolites derived from Red Sea micro- and macro-organisms, and discuss their biological potential whenever applicable. Moreover, the diversity of the Red Sea organisms is highlighted as well as their genomic potential. This review is a comprehensive study that compares the natural products recovered from the Red Sea in terms of ecological role and pharmacological activities.

## 1. Introduction

**Figure 1:**
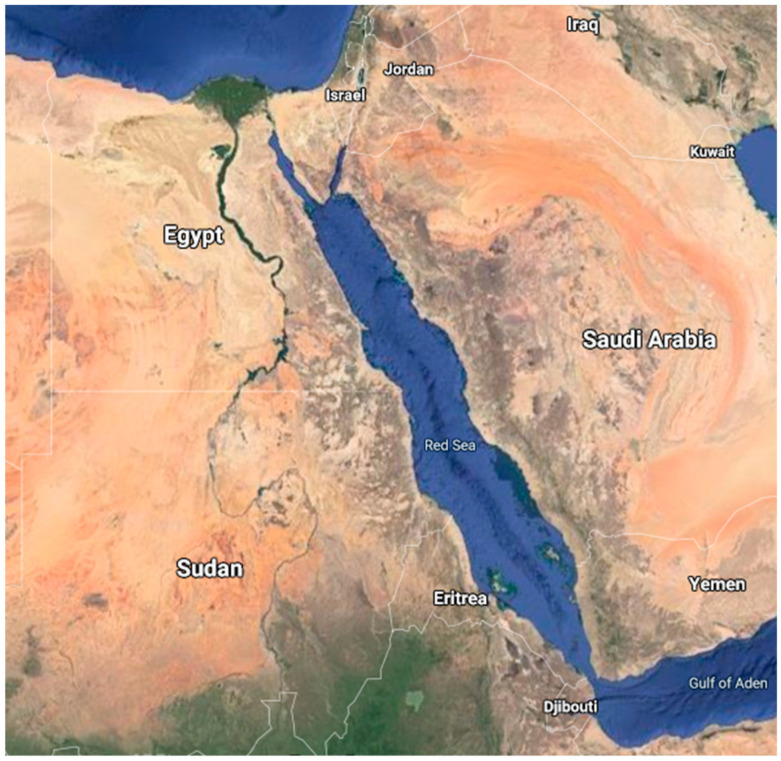
A satellite image of the Red Sea. The marine organisms were collected from Egypt (El Gouna, Hurghada, Ras Gharib, Ras Muhammad, Safaga, Gulf of Aqaba, and Sharm El-Sheikh), Saudi Arabia (Hakel area, Jazan, Obhur, Al-Lith, Jeddah, Salman Gulf and Yanbu), Israel (Gulf of Eilat), Eritrea (Dahlak archipelago and Massawa), Djibouti (Ardoukoba), Jordan (Aqaba) and Yemen (Hanish Islands).

Around 24 million years ago, the separation of the African and Arabian plates created the Red Sea [1]. Since then, the Red Sea has been characterized by exclusive assets such as location, relatively high temperature and relatively young geologic age. In the north, the Suez Canal connects the Mediterranean Sea with the Red Sea. While in the south, the Red Sea is connected to the Gulf of Aden and the Indian Ocean through Bab El-Mandeb Strait (Figure 1). The Red Sea exhibits an enormous diversity of life in all domains of life [2]. However, its biosphere is still underexplored and poorly understood [3,4]. Since 2000, no less than 58 new endemic species were identified in the Red Sea, for example, *Inermonephtys aramco* from the southern region [5,6]. These new species expanded our understanding of the previously discovered organisms such as *Aglaophamus lobatus*, *Aglaophamus* cf. *verrilli* and *Micronephthys stammeri* [6]. The Red Sea acts as a unique source of biological diversity by exporting species and genetic lineages for the global marine biodiversity patterns [7,8,9].

The Red Sea biosphere is divided into macrofauna (organisms larger than 0.5 or 1 mm in diameter) [10], meiofauna (organisms less than 0.5 mm in diameter) [11] and microorganisms [12,13,14]. In general, biodiversity may be influenced by nutrients localization [15], sediment particle size ranges [15], salinity, degree of oxygenation [16], depth (pressure) [17,18], temperature [19], organic matter, hydrodynamics [20], light conditions [21], as well as any natural or anthropogenic changes in these abiotic factors [22]. The Red Sea has an oligotrophic nature, which affects the biodiversity due to the deficiency of major nutrients, including nitrate, ammonium, phosphate and silica [21]. The most oligotrophic location is the central northern region owing to weak mixing and limited nutrient flux [2,23]. However, in the south, nutrient-rich flux from the Indian Ocean compensates for oligotrophy [24,25]. It is important to notice that the marine ecosystem has a capacity to equilibrate different factors including the major nutrients [26]. A salinity gradient is observed from north to south (42 to 37 psu) [1,27]. Surface temperatures vary from 24 to 35 °C in spring to summer, respectively [27]. Due to abiotic factors, the central Red Sea holds the highest species richness of marine organisms [27].

The anthropogenic activity of SCUBA diving (self-contained underwater breathing apparatus) was reported to negatively affect the corals which may lead to imbalances in the associated corallivorous and herbivorous fish [28]. The sedimentation rate changes depending on the diving intensities [28]. Moreover, the anthropogenic activities are related to warming of the Red Sea which was initiated in the mid-90s, with an 0.7 °C increment after 1994 [29]. Since the Red Sea is a bio-source for the global marine biodiversity, artificial conditions might be used to simulate this dynamicity to scrutinize the anthropogenic negative effects [30]. 

On the other hand, marine environment has proven to be a very rich source of diverse natural products with biological activities such as anticancer, antibacterial, antifungal, antiviral and antiparasitic [31,32,33,34]. The global marine pharmaceutical clinical pipeline comprises about 30 compounds originating from different marine invertebrates and marine microorganisms. These 30 compounds include 8 approved drugs by the most representative approving agencies and 22 drug candidates in phase I, II or III of drug development clinical phases [35,36,37,38,39]. Marine invertebrates and associated microorganisms are capable of synthesizing diverse classes of secondary metabolites and, in some cases, novel chemical leads that have never been discovered in terrestrial counterparts.

In March 2017, a review article containing a listing of natural products, isolated from Red Sea marine organisms, was published [40]. This published review covered the literature until the year 2014, and presented the chemical structures of 435 natural products together with their biological activities whenever applicable. The natural products mentioned in this published review were categorized according to their chemical classes, including terpenes, alkaloids, sterols, steroidal glycosides and other metabolites. 

In our review, we conducted a comprehensive literature search covering the time period until December 2019, with the inclusion of extra five years (from 2015 to 2019). We found that a total of 677 marine natural products were isolated form Red Sea marine organisms until the end of 2019. Publications that describe extracts and structurally uncharacterized marine natural products have been excluded from our review. The structures and biological activities of selected 111 marine natural products are illustrated in this review, and categorized according to their isolation source (marine organism), including microorganisms, invertebrates, algae and sea grasses. The presented 111 marine natural products were selected due to their potent biological activities. These selected compounds exhibited a wide range of potent biological activities, such as antioxidant, anticonvulsant, anticancer and anti-infective activities. In the Supplementary data, a list of the remaining 566 marine natural products from the Red Sea (with lower or no biological activities) is presented in Table S1.

**Figure 2:**
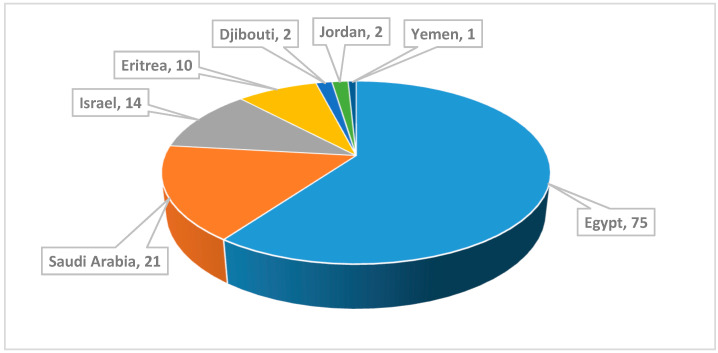
Numbers of marine organisms according to the location of collection in the Red Sea.

**Figure 3:**
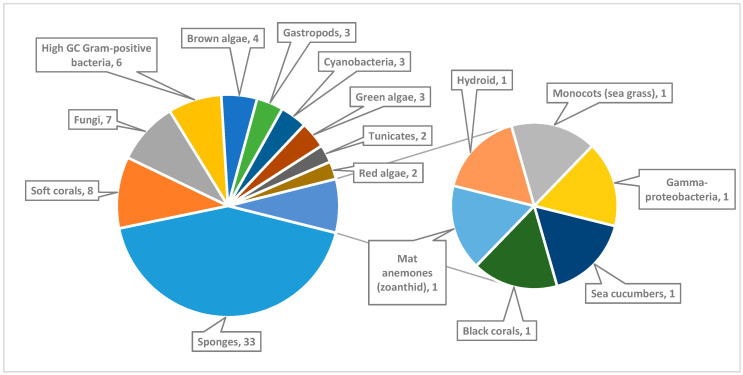
Numbers of marine organisms collected from the Red Sea.

The review illustrates also the geographical distribution of the collection sites of the marine organisms. Marine organisms, which are mentioned in this review, were collected from the Red Sea coasts of seven countries (Figure 2). These countries include Egypt, Saudi Arabia, Israel, Eritrea, Djibouti, Jordan and Yemen. The locations of collection of each marine organism are presented in Table S2 in the Supplementary data. The numbers of the collected marine micro- and macro-organisms from the Red Sea are presented in Figure 3.

**Figure 4:**
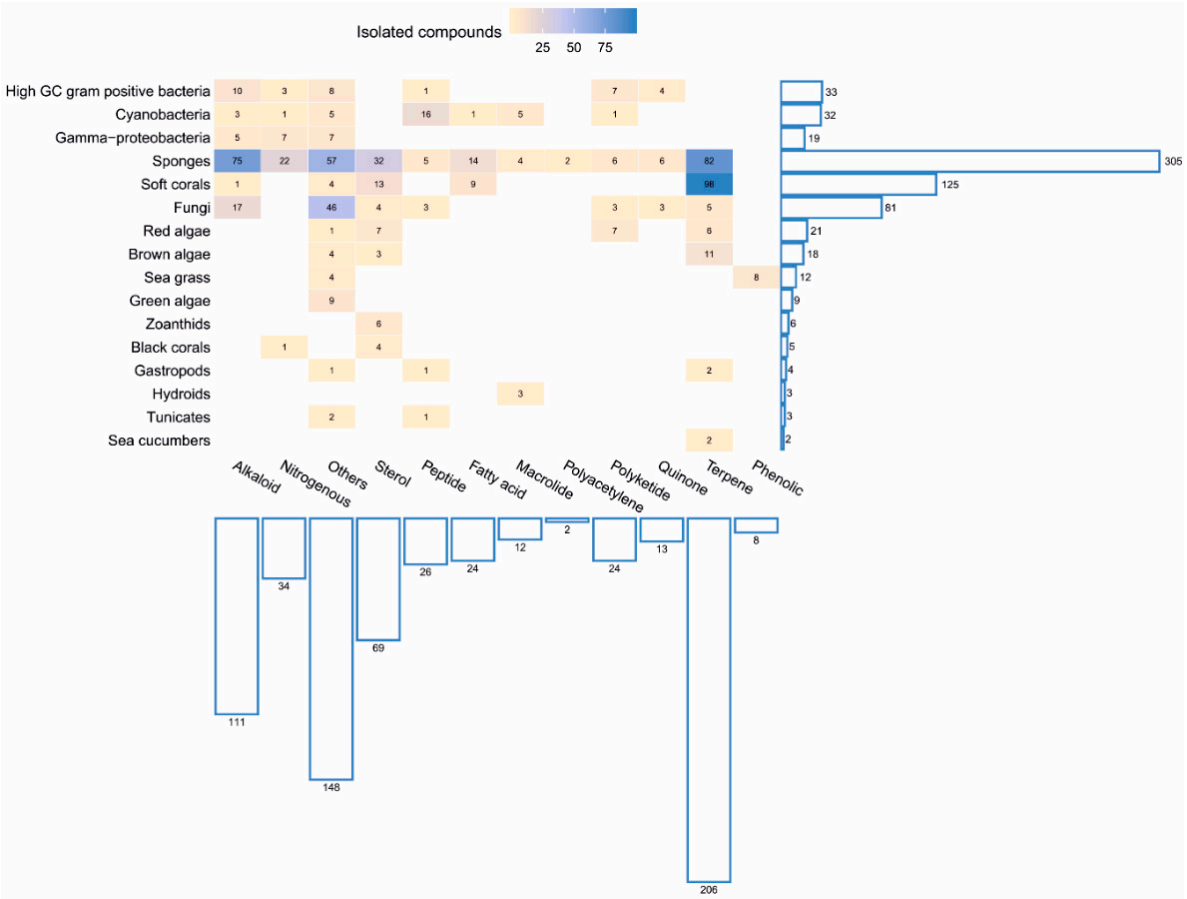
Numbers and chemical classes of the isolated compounds and their isolation sources (marine organisms).

Taxonomy of the marine organisms collected from the Red Sea is also presented in Table S3 in the Supplementary data. The correlation between the numbers and chemical classes of the isolated compounds and their isolation sources (marine organisms) is shown in Figure 4. Moreover, a comprehensive study on the genomic potential of the Red Sea organisms is included in the review. To the best of our knowledge, this is the first comprehensive review that presents and discusses the marine natural products of the Red Sea until the end of 2019, with regard to their chemistry, biological activities, biodiversity of the collected marine organisms, geographical distribution of the locations of collection of the marine organisms and the genomic potential of the marine organisms of the Red Sea.

## 2. Marine Natural Products Isolated from Marine Microorganisms

### 2.1. Marine Bacteria

**Figure 5:**
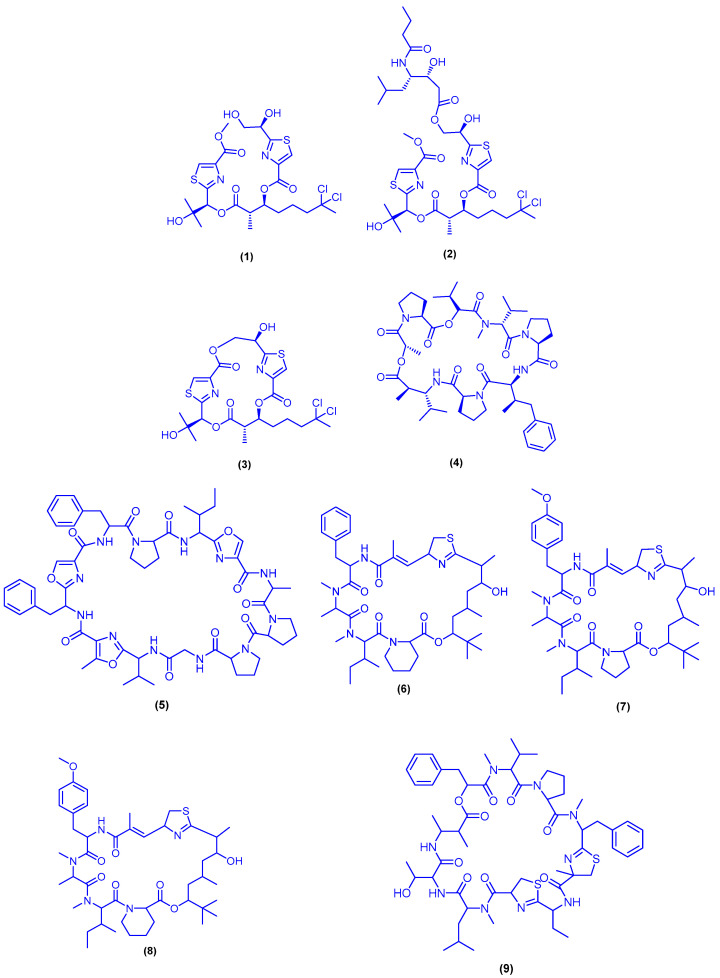
Chemical structures of lyngbyabellin O (**1**), lyngbyabellin P (**2**), lyngbyabellin G (**3**), dolastatin 16 (**4**), wewakazole B (**5**), apratoxin H (**6**), apratoxin A sulfoxide (**7**), grassypeptolide D (**8**) and grassypeptolide E (**9**).

Nuclear magnetic resonance- and mass spectrometry-guided fractionation of the extract of *Okeania* sp. marine cyanobacterium, collected from the Saudi coast from the Algetah Alkabira reef near Jeddah, afforded the bioactive metabolites lyngbyabellin O (**1**), lyngbyabellin P (**2**), lyngbyabellin G (**3**) and dolastatin 16 (**4**) (Figure 5). The identified compounds **1**, **2**, **3** and **4** were evaluated for their antifouling activity, using *Amphibalanus amphitrite* larvae. Compounds **1**, **2** and **3** displayed remarkable antifouling activity with EC_50_ values of 0.38, 0.73 and 7.4 μM, respectively. Compound **4**, which is reported as a potent antifouling agent, was the most active one with EC_50_ value of 0.09 μM. On the other hand, compound **2** was cytotoxic to MCF7 cells with GI_50_ value of 9 µM, while compound **3** showed no cytotoxicity to MCF7 cells (GI_50_ > 160 µM). Lyngbyabellin G (**3**) exhibited cytotoxicity on MCF7, H460 and Neuro-2a cells with GI_50_ values of 120, 2.2 and 4.8 μM, respectively [41,42,43]. Okino and co-workers isolated a cytotoxic cyanobactin, wewakazole B (**5**), from the brownish-red filamentous cyanobacterium *Moorea producens* (formerly *Lyngbya majuscula*), which was collected near Jeddah, Saudi Arabia (Figure 5). Wewakazole B (**5**) exhibited cytotoxic activity towards human MCF7 breast cancer cells (IC_50_ = 0.58 μM) and human H460 lung cancer cells (IC_50_ = 1 μM). Unlike what was reported for many cyanobactins, wewakazole B (**5**) exhibited no metal-binding activity at 89 μM, excluding its function as siderophore [44].

A cultivated *Moorea producens,* collected from the Nabq Mangroves in the Gulf of Aqaba near Sharm el-Sheikh, Egypt, was the source of the cyclic depsipeptides apratoxin H (**6**) and apratoxin A sulfoxide (**7**) (Figure 5). Apratoxin H (**6**) showed potent cytotoxicity to human NCI-H460 lung cancer cells with IC_50_ value of 3.4 nM, while apratoxin A sulfoxide (**7**) was 26-fold less active than apratoxin H (**6**) (IC_50_ = 89.9 nM). It is worth mentioning that the S-oxide function in apratoxin A sulfoxide (**7**) led to about 36-fold reduction in potency in comparison with its parent compound apratoxin A, the well-studied analog as anticancer agent [45]. Two macrocyclic depsipeptides, namely grassypeptolide D (**8**) and grassypeptolide E (**9**), were isolated from the marine cyanobacterium *Leptolyngbya* sp., which was collected from the *SS Thistlegorm* shipwreck in the Red Sea (Figure 5). Grassypeptolide D (**8**) and grassypeptolide E (**9**) exhibited potent cytotoxicity at nanomolar concentrations to HeLa cells (IC_50_ = 335 and 192 nM, respectively) and mouse neuro-2a blastoma cells (IC_50_ = 599 and 407 nM, respectively) [46].

**Figure 6:**
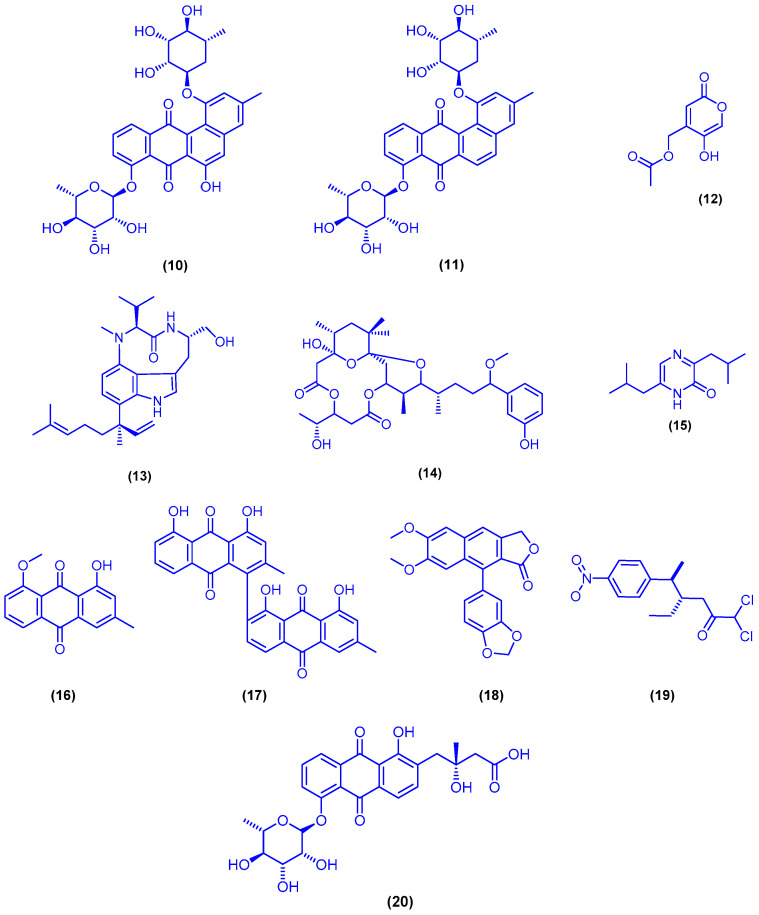
Chemical structures of actinosporin C (**10**), actinosporin D (**11**), saadamycin (**12**), lyngbyatoxin A (**13**), debromoaplysiatoxin (**14**), 3,6-diisobutyl-2(1*H*)-pyrazinone (**15**), chrysophanol 8-methyl ether (**16**), asphodelin (**17**), justicidin B (**18**), ayamycin (**19**) and fridamycin H (**20**).

The actinosporin analogues actinosporin C (**10**) and actinosporin D (**11**) were isolated from the calcium alginate beads culture of sponge-associated *Actinokineospora* sp. strain EG49, which was cultivated from the sponge *Spheciospongia vagabunda* (Figure 6). Actinosporin C (**10**) and actinosporin D (**11**) exhibited significant antioxidant activity at a concentration of at 1.25 µM, and protective capacity from the genomic damage induced by hydrogen peroxide in the human promyelocytic (HL-60) cell line. Compounds **10** and **11** did not show any significant cytotoxicity against the mammalian cells HL-60 after 4 h at concentrations of up to 50 µM, while after 24 h, significant cytotoxicity for compound **10** was observed at a concentration of 50 µM [47]. In another study, compounds **10** and 11 were also isolated from *Actinokineospora spheciospongiae* sp. nov, which was isolated from the sponge *Spheciospongia vagabunda* that was collected from offshore Ras Mohamed, Egypt [48]. Saadamycin (**12**) was isolated from the endophytic *Streptomyces* sp. Hedaya48, isolated from the marine sponge *Aplysina fistularis*, which was collected from the Egyptian coast of Sharm El-Sheikh (Figure 6). Saadamycin (**12**) displayed significant antifungal activity against nine dermatophytes and other clinical fungi, with IC_50_ values of 1–5.16 µg/mL [49]. Chromatographic separation of the organic extract of the marine cyanobacterium *Moorea producens*, collected from the Saudi coast near Obhur, led to the isolation of lyngbyatoxin A (**13**) and debromoaplysiatoxin (**14**) (Figure 6) [50]. In a previous report, compound **14** was also isolated form the same marine cyanobacterium, which was collected near Jeddah [51]. Compounds **13** and **14** exhibited potent antiproliferative activity against HeLa cancer cells with IC_50_ value of 9.2 nM and 3.03 µM [50]. The (1*H*)-pyrazinone alkaloid 3,6-diisobutyl-2(1*H*)-pyrazinone (**15**) was isolated from the tunicate-derived actinomycete *Streptomyces* sp. Did-27, collected from a location near Obhur, Saudi Arabia (Figure 6). The alkaloid 15 displayed selective cytotoxicity against HCT-116 cell line with IC_50_ value of 1.5 µg/mL [52]. The four bioactive metabolites chrysophanol 8-methyl ether (**16**), asphodelin (**17**), justicidin B (**18**) and ayamycin (**19**) were isolated from the actinomycete *Nocardia* sp. ALAA 2000, derived from the marine red alga *Laurencia spectabilis,* which was collected from the Egyptian coast in Ras-Gharib (Figure 6). Compounds **16**–**19** showed significant antimicrobial potential against eight pathogenic bacterial strains, including Gram-positive and Gram-negative bacteria, as well as six fungal strains, with MIC values ranging from 0.1 to 10 µg/mL [53]. Fridamycin H (**20**) was isolated from the elicited marine sponge-derived bacterium *Actinokineospora spheciospongiae* sp. nov., collected from offshore Ras Mohamed, Egypt. Fridamycin H (**20**) exhibited antitrypanosomal activity (against *Trypanosoma brucei* strain TC221) after 48 and 72 h, with IC_50_ values of 7.18 and 3.35 μM, respectively. Furthermore, compound **20** has no cytotoxic activity against J774.1 macrophages (IC_50_ > 200 μM) [48].

### 2.2. Marine Fungi

**Figure 7:**
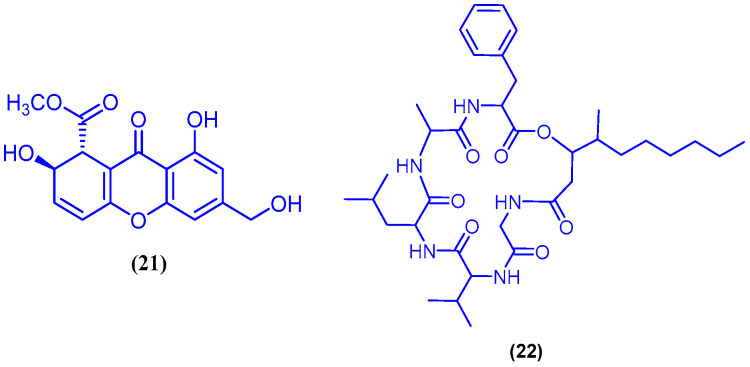
Chemical structures of AGI-B4 (**21**) and scopularide A (**22**).

The xanthone derivative AGI-B4 (**21**) and the cyclic depsipeptide scopularide A (**22**) were isolated from solid rice cultures of the marine-derived fungus *Scopulariopsis* sp., which was obtained from the hard coral *Stylophora* sp. (Figure 7). Compounds **21** and **22** showed significant cytotoxicity against L5178Y mouse lymphoma cells with IC_50_ values of 1.5 and 1.2 µM, respectively, which were lower than that of the positive control kahalalide F (IC_50_ = 4.3 µM) [54].

## 3. Marine Natural Products Isolated from Marine Invertebrates

### 3.1. Sponges

**Figure 8:**
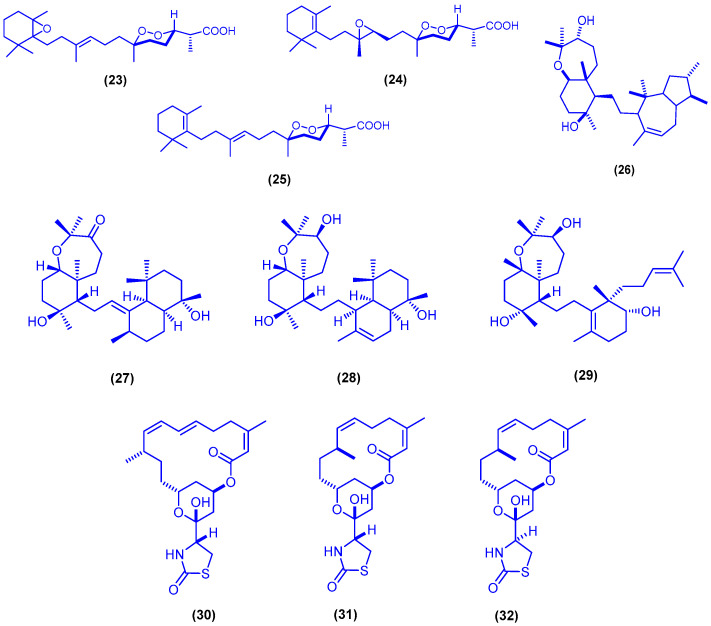
Chemical structures of compound **23**, compound **24**, (−)-muqubilin A (**25**), sipholenol A (**26**), sipholenone E (**27**), sipholenol L (**28**), siphonellinol D (**29**), latrunculin A (**30**), latrunculin B (**31**) and 16-*epi*-latrunculin B (**32**).

Several cyclic peroxide norterpenoids have been isolated from the sponge *Diacarnus erythraeanus*, which was obtained from Elfanadir (Hurghada Coast, Egypt). These metabolites include norsesterterpene derivatives **23**, **24** and (−)-muqubilin A (**25**) (Figure 8). The three compounds displayed potent growth inhibitory activity against seven different human cancer cell lines, the Hs683 oligodendroglioma, MCF-7 breast, PC-3 prostate, U373 and U251 glioblastoma, SKMEL-28 melanoma and A549 non-small cell lung cancer cells, with a range of IC_50_ values from 1 to 8 μM. Further studies on (−)-muqubilin A (**25**) revealed that it possesses cytotoxicity without selectivity between normal and cancer cells. It induces reactive oxygen species (ROS) production in cancer cells; it is less likely that (−)-muqubilin A (**25**) exerts its cytotoxic action via stimulation of pro-apoptotic processes. It is worth mentioning that, the presence of the free carboxylic acid group in the structure of these metabolites may be critical for their growth inhibitory activity in various cancer cell lines [55]. Sipholenol A (**26**) is a sipholane triterpenoid that was isolated from the marine sponge *Callyspongia siphonella* (formerly known as *Siphonochalina siphonella*), which was collected from Hurghada, at the Egyptian coast (Figure 8) [56]. In another three published studies, compound **26** was also isolated from the marine sponge *Callyspongia siphonell*, collected from Sharm Obhur in Jeddah, Saudi Arabia [57] and from Hurghada in Egypt [58,59]. It was shown that Sipholenol A (**26**)—at non-cytotoxic concentration (5 µM)- could increase the sensitivity of the resistant cervical adenocarcinoma KB-C2 cells by 16 times towards colchicine through the reversal of P-Glycoprotein-mediated MDR. Compound (**26**) did not affect the IC_50_ value of colchicine to the parent cell line KB-3-1(which do not express P-gp) [56]. In a later report, the same research group isolated from the marine sponge *Callyspongia* (*Siphonochalina*) *siphonell* other sipholane triterpenoids with the perhydrobenzoxepine nucleus including sipholenone E (**27**), Sipholenol L (**28**) and siphonellinol D (**29**) (Figure 8) [60]. Compound **28** was also isolated from the marine sponge *Siphonochalina siphonella*, collected from Sharm Obhur, Jeddah, Saudi Arabia [57]. Compound **29** was also isolated from the sponge *Siphonochalina siphonella*, which was collected near Hurghada, Egypt [58]. The sipholane triterpenoid 27 was shown to be a superior compound compared to sipholenol A (**26**), in reversing the P-gp-mediated multidrug resistance. Compounds **28** and **29** were comparable to sipholenol A (**26**) in potency. Compounds **26**–**29** were found to be non-toxic to the human epidermoid cancer cells KB-3-1 and KB-C2, with IC_50_ value higher than 50 µM for both cell lines [60]. Latrunculin A (**30**), latrunculin B (**31**) and 16-*epi*-latrunculin B (**32**) were isolated from the marine sponge *Negombata magnifica*, collected from the coast of Eilat (Figure 8). Latrunculins are macrolides that were reported to cause unique microfilament inhibition due to one-to-one reversible complex with monomeric actin, disrupting its polymerization and consequently its functions within the cell. Latrunculin A (**30**) and latrunculin B (**31**) were able to show activity in the actin-disruption assay in concentrations of 0.5–1 and 0.5–10 µg/mL, respectively (these are the concentrations where microfilament loss is detected). Compound **32** was less active than compounds **30** and **31**, and showed activity at 5–10 µg/mL [61]. Latrunculin B (**31**) inhibited also the migration of B16B15b tumor cell in a wound-healing assay at 1 µM [62].

**Figure 9:**
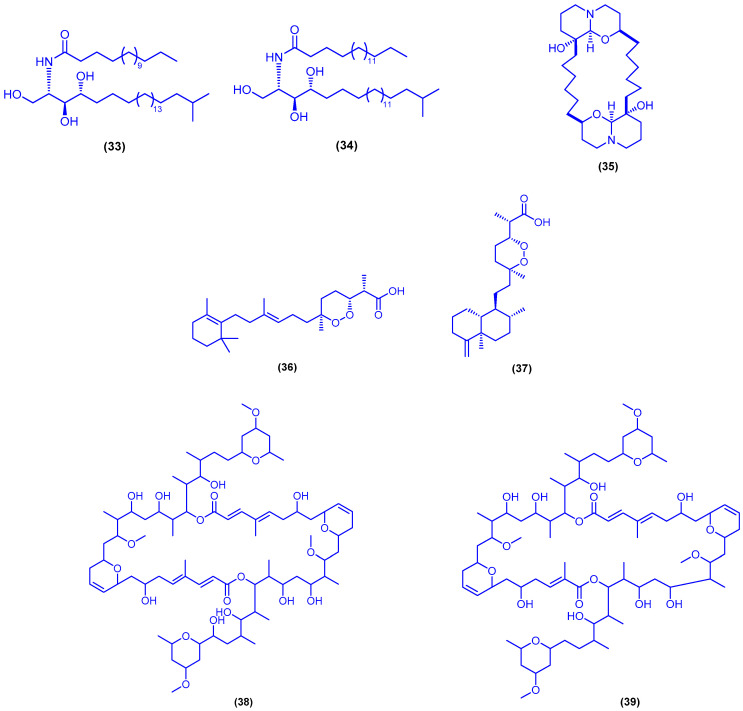
Chemical structures of compounds **33**, compound **34**, araguspongine C (**35**), muqubilin (**36**), sigmosceptrellin B (**37**), swinholide I (**38**), hurghadolide A (**39**).

Compounds **33** and **34** were isolated as a ceramide mixture from the marine sponge *Negombata corticata*, which was collected from Safaga in Egypt (Figure 9). The mixture was evaluated for its in vitro anticonvulsant activity in the pentylenetetrazole-induced seizure model. It showed antiepileptic effect comparable to that of the reference drug diazepam [63]. The bis-1-oxaquinolizidine *N*-oxide alkaloid araguspongine C (**35**) was isolated from the sponge *Xestospongia exigua*, which was obtained from vertical reef slopes at Bayadha, north of Jeddah on the Saudi Arabian coast (Figure 9). Araguspongine C (**35**) was reported to be active against *Plasmodium falciparum* African (D6) clone with an IC_50_ value of 0.67 µg/mL and selectivity index >7.1, and against *P. falciparum* Indochina (W2) clone with IC_50_ value of 0.28 µg/mL and selectivity index >17. Additionally, araguspongine C (**35**) was also shown to possess high activity against the H37Rv strain of *Mycobacterium tuberculosis* with MIC value of 3.94 µM [64]. The norsesterterpene peroxide acids muqubilin (**36**) and sigmosceptrellin B (**37**) were isolated from the marine sponge *Diacarnus erythraeanus*, which was obtained from the Egyptian coast northeast of Hurghada (Figure 9) [65]. In another published study, muquabilin (**36**) was also identified and isolated from the sponge *Diacarnus erythraenus*, which was collected from El Qusier, 120 km south of Hurghada, Egypt [66]. Sigmosceptrellin B (**37**) was also identified and isolated in another two studies from the same marine sponge, which was collected from Hurghada [55,67]. Muqubilin (**36**) and sigmosceptrellin B (**37**) could show potent in vitro antiparasitic activity against *Toxoplasma gondii* at a concentration of 0.1 µM without significant toxicity to HFF or L929 cells. Sigmosceptrellin B (**37**) exhibited also a significant in vitro antimalarial activity against *Plasmodium falciparum* (D6 and W2 clones) with IC_50_ values of 1.2 and 3.4 µg/mL, respectively. The functional group homology of compounds **36** and **37** with artemisinin suggests that the bioactivity may be due to the peroxide moieties [65]. The sponge *Theonella swinhoei*, which was collected in Hurghada, was reported to be the source of swinholide I (**38**) (a macrolide with a symmetric 44-membered dilactone skeleton) and hurghadolide A (**39**) (asymmetric 42-membered dilactone) (Figure 9). Both compounds showed in vitro cytotoxicity against human colon adenocarcinoma (HCT-116) with IC_50_ values of 5.6 and 365 nM, respectively. However, on measuring the actin microfilament-disrupting effects, hurghadolide A (**39**) showed 10 times higher potency compared to swinholide I (**38**) (these compounds caused disruption of the actin cytoskeleton at concentrations of 7.3 and 70 nM, respectively). This suggested that swinholide I (**38**) has higher selectivity for cancer cells and there might be other mechanisms that initiate cytotoxicity in cancer cells. The nonspecific toxicity of hurghadolide A (**39**) might be also indicated by its higher potency against *Candida albicans* (MIC = 31.1 µg/mL for compound **39** vs. 500 µg/mL for compound **38**) [68].

**Figure 10:**
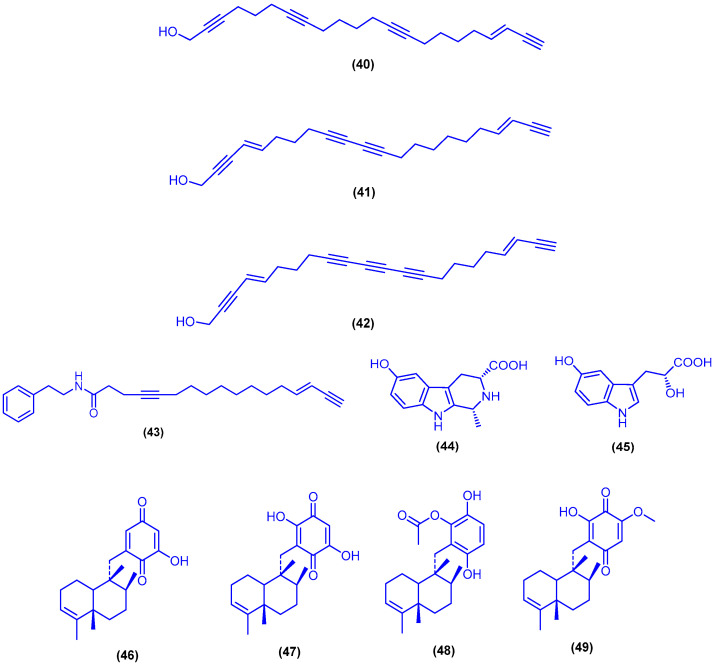
Chemical structures of callyspongenol A (**40**), callyspongenol B (**41**), dehydroisophonochalynol (**42**), callyspongamide A (**43**), hyrtioerectine B (**44**), hyrtioerectine C (**45**), avarone A (**46**), 3′,6′-dihydroxyavarone (**47**), avarol C (**48**) and avarone E (**49**).

The C22-polyacetylenic alcohols callyspongenol A (**40**) and callyspongenol B (**41**) together with dehydroisophonochalynol (**42**) were isolated from the organic extract of the sponge *Callyspongia* sp., which was collected from the Hurghada, Egypt (Figure 10). Compounds **40**, **41** and **42** displayed cytotoxicity against P388 and HeLa cells with a range of IC_50_ values ranging from 2.2 to 5.1 µg/mL [69]. A cytotoxic polyacetylenic amide, callyspongamide A (**43**), was isolated from the marine sponge *Callyspongia fistularis* collected from Hurghada, Egypt (Figure 10). Callyspongamide A (**43**) exhibited a cytotoxic activity against HeLa cells with an IC_50_ value of 4.1 µg/mL [70]. The two indole alkaloids hyrtioerectine B (**44**) and hyrtioerectine C (**45**) were isolated from the marine sponge *Hyrtios erectus*, which was collected from Hurghada, Egypt (Figure 10). The alkaloids 44 and 45 exhibited cytotoxic activity against HeLa cells with IC_50_ values of 5 and 4.5 µg/mL, respectively [71]. The dichloromethane extract of the sponge *Dysidea cinereal*, which was collected near Ras Zaatir in the Gulf of Eilat, yielded the avarol and avarone derivatives avarone A (**46**), 3′,6′-dihydroxyavarone (**47**), avarol C (**48**) and avarone E (**49**) (Figure 10). The avarone derivatives 47 and 49 showed activity as inhibitors of HIV-1 reverse transcriptase, with IC_50_ values of 5 and 1 µg/mL, respectively. The cytotoxicity of the compounds was also measured against P388 mouse leukemia cells. The IC_50_ values were found to be 0.6, 1.2 and <0.6 µg/mL for compounds **46**–**48**, respectively [72].

**Figure 11:**
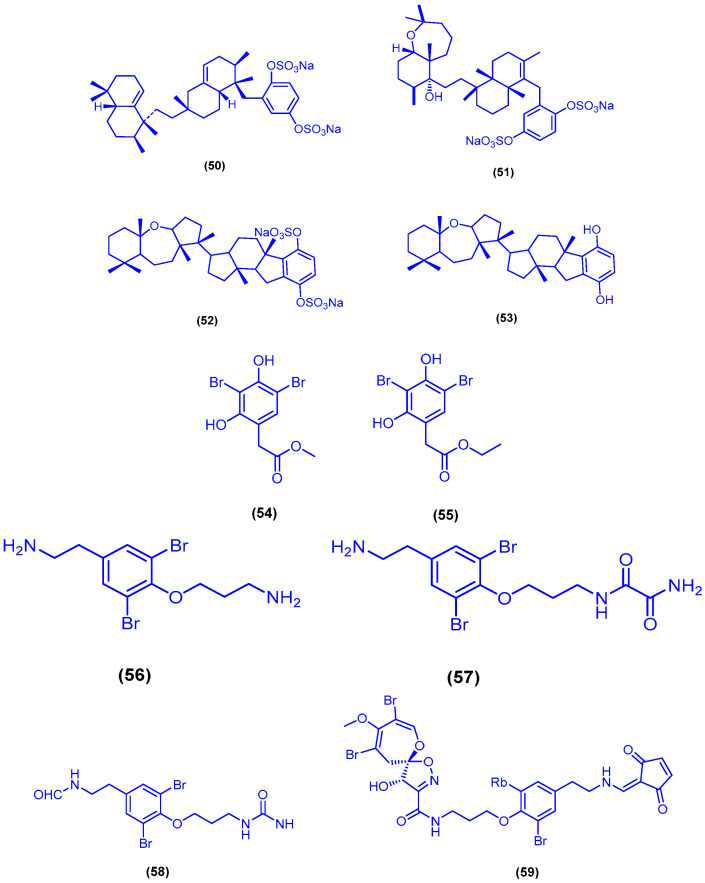

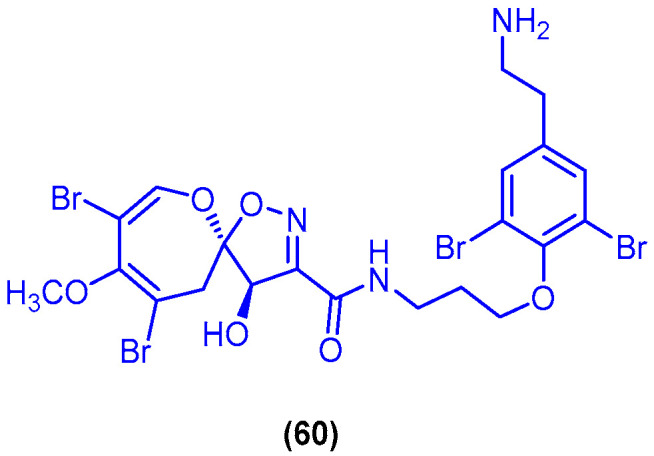
Chemical structures of toxiusol (**50**), shaagrockol C (**51**), toxicol A (**52**), toxicol B (**53**), subereaphenol B (**54**) and subereaphenol C (**55**), moloka’iamine (**56**), moloka’iakitamide (**57**), ceratinine H (**58**), psammaplysin E (**59**) and psammaplysin A (**60**).

In 1993, a bioassay-guided fractionation of an organic extract of the sponge *Toxiclona toxius* yielded the hexaprenoid hydroquinones toxiusol (**50**), shaagrockol C (**51**), toxicol A (**52**) and toxicol B (**53**) (Figure 11) [73]. In the same year, another study published the isolation of toxiusol (**50**), toxicol A (**52**) and toxicol B (**53**) form the marine sponge *Toxiclona toxius* [74]. One year earlier, shaagrockol C (**51**) was also identified and isolated from the sponge *Toxiclona toxius* [75]. Compounds **50**, **51**, **52** and **53** exhibited inhibitory activity of both DNA polymerizing functions of HIV-1 RT (RNA-dependent DNA polymerase RDDP and DNA-dependent DNA polymerase DDDP), but did not inhibit the RT-associated ribonuclease H activity. The hexaprenoid hydroquinones 50, 51, 52 and 53 inhibited RDDP function with IC_50_ values of 1.5, 3.3, 3.1 and 3.7 µM, and DDDP function with IC_50_ values of 6.6, 0.8, 2.7 and 8.2 µM, respectively [73]. Chemical investigation of *Suberea mollis*, a marine sponge collected from Hurghada, yielded the two brominated compounds subereaphenol B (**54**) and subereaphenol C (**55**) [76]. In a more recent study, subereaphenol C (**55**) was also isolated from the verongid sponge *Suberea* species, which was collected off Yanbu in Saudi Arabia [77]. Biological evaluation of compounds **54** and **55**, using the 2,2-diphenyl-1-picrylhydrazyl radical (DPPH) solution-based chemical assay, revealed a significant antioxidant activity (Figure 11). The high antioxidant activity could be due to the phenolic nature of the two compounds. None of compounds **54** and **55** exhibited any cytotoxicity against the human colon cancer cells, at a concentration of 10 µg/mL [76]. In another study, compound **55** showed antiproliferative activity against HeLa cells with IC_50_ value of 13.3 µM and no antimigratory activity against MDA-MB-321 cells (IC_50_ > 50 µM) [77]. The two brominated compounds moloka’iamine (**56**) and moloka’iakitamide (**57**) were isolated from the marine sponge *Pseudoceratina arabica*, which was collected from Sharm El-Sheikh [78] and Hurghada [79] in Egypt (Figure 11). Compounds **56** and **57** displayed significant parasympatholytic effects on isolated rabbit heart and jejunum, with no cytotoxicity against the HCT-116 cells, at a concentration of 10 µg/mL [78]. Moloka’iamine (**56**) was also isolated from the same marine sponge (*Pseudoceratina arabica*) collected from Anas Reef off Obhur in Saudi Arabia [80]. The dibrominated alkaloid ceratinine H (**58**) together with psammaplysin E (**59**) were isolated from the Red Sea marine Verongid sponge *Pseudoceratina arabica*, collected from Anas Reef off Obhur (Figure 11). The two alkaloids **58** and **59** were examined for their antimigratory activity against the highly metastatic human breast cancer cell line MDA-MB-231, and for their antiproliferative activity against HeLa cells. Ceratinine H (**58**) and psammaplysin E (**59**) exhibited antiproliferative activity against HeLa cells with IC_50_ values of 2.56 and 2.19 µM, respectively. Compound **59** displayed also high antimigratory activity against MDA-MB-231 cells with IC_50_ value of 0.31 µM [80]. Psammaplysin A (**60**) was isolated from the marine sponge *Pseudoceratina arabica*, which was obtained from both Anas Reef off Obhur at the Saudi coast [80] and Sharm El-Sheikh at the Egyptian coast [78]. In another report, psammaplysin A (**60**) and psammaplysin E (**59**) were isolated and identified from the verongid sponge *Aplysinella* species, harvested from Jizan, Saudi Arabia. Psammaplysin A (**60**) exhibited cytotoxic activity against MDA-MB-231, HeLa and HCT 116 cell lines with IC_50_ values of 3.9, 8.5 and 5.1 µM, respectively. While, psammaplysin E (**59**) showed a more potent cytotoxic activity against the same cell lines with IC_50_ values of 0.29, 2.1 and 3.7 µM, respectively [81].

**Figure 12:**
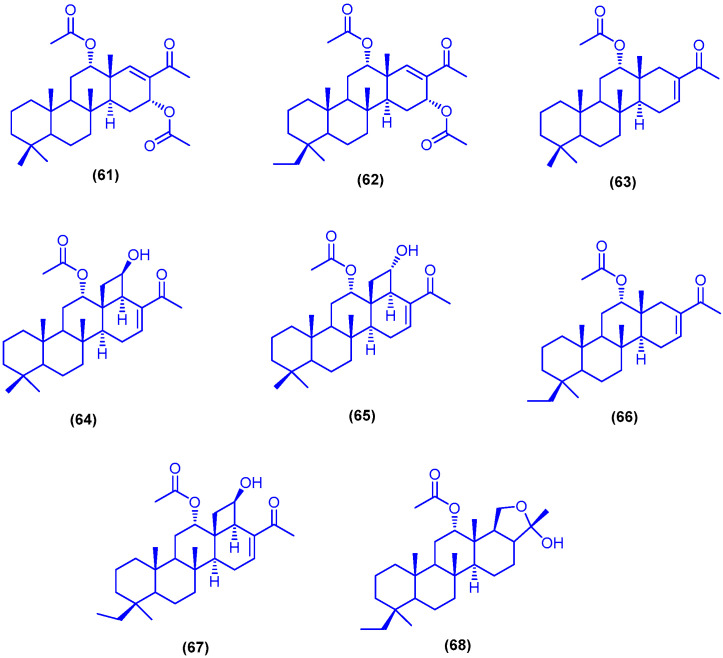
Chemical structures of phyllospongin A (**61**), phyllospongin B (**62**), phyllospongin C (**63**), phyllospongin D (**64**), phyllospongin E (**65**), compounds **66**–**68**.

The scalarane sesterterpene phyllospongin A (**61**), phyllospongin B (**62**), phyllospongin C (**63**), phyllospongin D (**64**), phyllospongin E (**65**), 12α-acetoxy20,24-dimethyl-25-norscalar-16-en-24-one (**66**), 12α-acetoxy-13β,18β-cyclobutan-20,24-dimethyl-24oxoscalar-16-en-25β-ol (**67**) and 12α-acetoxy-24,25-epoxy-24-hydroxy-20,24-dimethylscalarane (**68**) were isolated from the sponge *Phyllospongia lamellosa*, which was collected from Shaab Saad area northern Hurghada (Figure 12). Compounds **64**, **65** and **67** showed antibacterial activities against the Gram-positive pathogens *Staphylococcus aureus* ATCC25923 and *Bacillus subtilis* NCTC2116, with a range of MIC values from 1.7 to 2.5 µg/mL, and against the Gram-negative bacteria *Vibrio parahaemolyticus* NCTC10441 with MIC values of 6.8, 9.8 and 7 µg/mL, respectively. In addition, compounds **61**–**68** showed cytotoxic activities against three human cancer cell lines (HePG-2, MCF-7, and HCT-116), with a range of IC_50_ values from 0.29 to 2.1 µg/mL [82]. 

**Figure 13:**
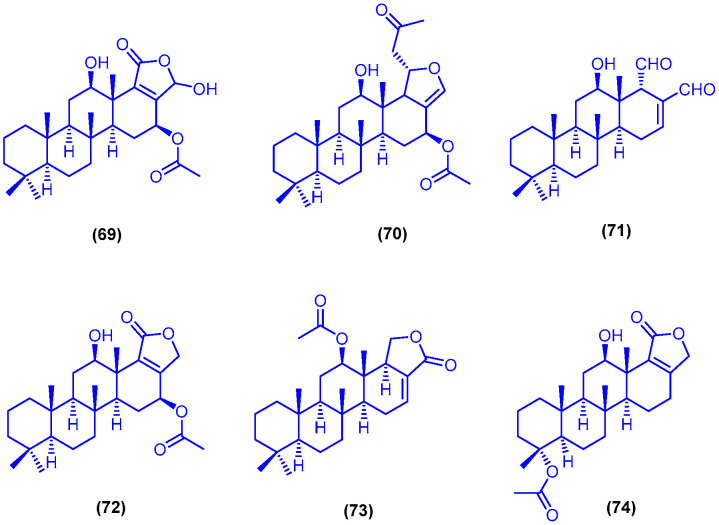
Chemical structures of compound **69**, heteronemin (**70**), compound **71**, sesterstatin 7 (**72**), 12-*epi*-24-deoxyscalarin (**73**) and 19 acetylsesterstatin 3 (**74**).

The scalarane-type sesterterpenes 12β,20-αdihydroxy-16β-acetoxy-17-scalaren-19,20-olide (**69**), heteronemin (**70**) and 12-deacetyl-12,18-di-*epi*-scalaradial (**71**) were isolated and identified from the marine sponge *Hyrtios erectus*, collected from Sharm El-Sheikh in Egypt (Figure 13). Compounds **69**–**71** displayed remarkable antiproliferative activities against the breast adenocarcinoma MCF-7 cells (IC_50_ = 12.7, 1.1 and 3.3 µM, respectively), the colorectal carcinoma HCT-116 cells (IC_50_ = 3.5, 0.7 and 3.4 µM, respectively) and the hepatocellular carcinoma HepG2 cells (IC_50_ = 9.6, 1.1 and 1.7 µM, respectively) [83]. The scalarane-type sesterterpenes sesterstatin 7 (**72**), 12-*epi*-24-deoxyscalarin (**73**) and 19-acetylsesterstatin 3 (**74**) were isolated from the sponge *Hyrtios erectus*, together with compounds **69**–**71** (Figure 13) [84]. Sesterstatin 7 (**72**) was also isolated from the sponge *Hyrtios erectus*, collected from Safaga in Egypt [85]. In another published study, compounds **72** and **73** were isolated from specimen of the marine sponge *Hyrtios erectus*, which was collected from Sharm El-Sheikh [83]. Compound **74** was also identified in another study and isolated from the sponge *Hyrtios erecta*, which was collected from El Quseir, 120 km south of Hurghada, Egypt [86]. Compounds **69**, **71**–**74** exhibited anti-tubercular activity against a nonvirulent *Mycobacterium tuberculosis* strain (ATCC 25177, H37Ra), with MIC values of 4.23, 5.05, 0.45, 1.12 and 4.39 µM, respectively. Sesterstatin 7 (**72**) exhibited also antibacterial activity against a strain of *Helicobacter pylori* (American Type Culture Collection, H.b., ATCC 700392), with MIC value of 4.39 µM. On the other hand, Compounds **69**–**71** showed significant cytotoxic activities against three human cancer cell lines (MCF-7, HCT-116 and HepG2) with a range IC_50_ values ranging from 0.4 to 12.4 µM [84]. 

**Figure 14:**
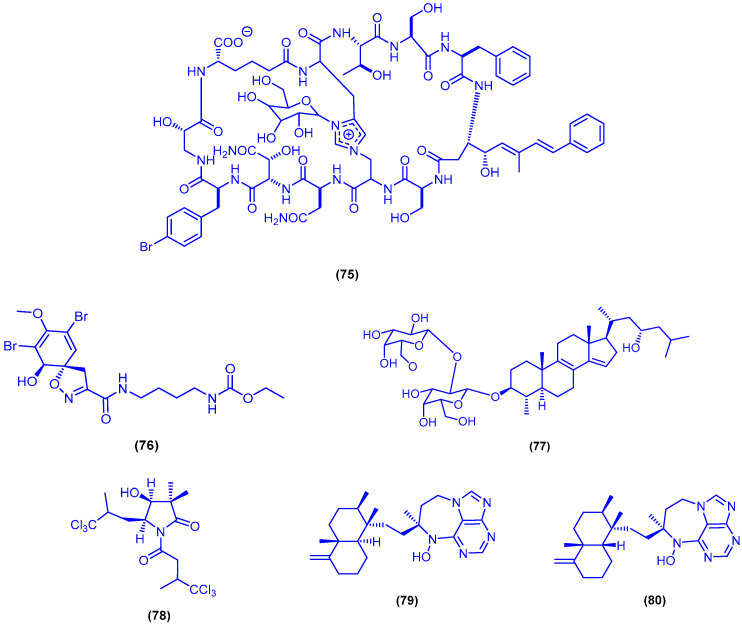
Chemical structures of theonellamide G (**75**), subereamolline A (**76**), eryloside A (**77**), dysidamide (**78**), asmarine A (**79**) and asmarine B (**80**).

Chromatographic fractionation of the organic extract of the sponge *Theonella swinhoei*, collected from Hurghada, led to the isolation of the bicyclic glycopeptide theonellamide G (**75**) (Figure 14). Compound **75** exhibited significant antifungal activity against the wild and the amphotericin B-resistant strains of the fungal pathogen *Candida albicans*, with IC_50_ values of 4.49 and 2 μM, respectively. It showed also cytotoxicity against HCT-16 cells with IC_50_ value of 6 μM [87]. Bioassay-guided fractionation of the extract of the sponge *Suberea mollis*, collected from Hurghada, afforded the brominated alkaloid subereamolline A (**76**) (Figure 14) [76,79]. Compound **76** was a potent inhibitor of the migration and the invasion of MDA-MB-231 cells (a highly metastatic human breast cancer), with IC_50_ value of 1.7 µM [79]. The steroidal glycoside eryloside A (**77**) was purified from the methanol extract of the marine sponge *Erylus lendenfeldi*, which was collected from the north of Hurghada (Figure 14). Compound **77** exhibited cytotoxicity against the yeast strain *Saccharomyces cerevisiae* with IC_50_ value of 3.5 µM, and higher cytotoxicity against its mutant strain (deficient in double strand break repair), with IC_50_ value of 0.8 µM [88]. The polychlorinated pyrrolidinone derivative dysidamide (**78**) was isolated from a sponge *Dysidea* sp. [89] and the sponge *Dysidea herbacea* [90]. In a later study, dysidamide (**78**) was isolated again from the dichloromethane extract of the marine sponge *Lamellodysidea herbacea*, which was collected from the Red Sea during the Ardoukoba expedition (Figure 14). Dysidamide (**78**) led to entire and rapid death of mesencephalic and cortical murine neurones at a dose of 0.8 µg/mL [91]. The two cytotoxic metabolites asmarine A (**79**) and asmarine B (**80**) were identified and isolated from the marine sponge *Raspailia* sp., collected from Dahlak archipelago, Eritrea (Figure 14) [92,93]. Compounds **79** and **80** were found to have cytotoxicity against P-388 murine leukemia cells, A-549 human lung carcinoma cells, HT-29 human colon carcinoma cells and MEL-28 human melanoma cells, with IC_50_ range from 0.12 to 1.8 µM [92].

**Figure 15:**
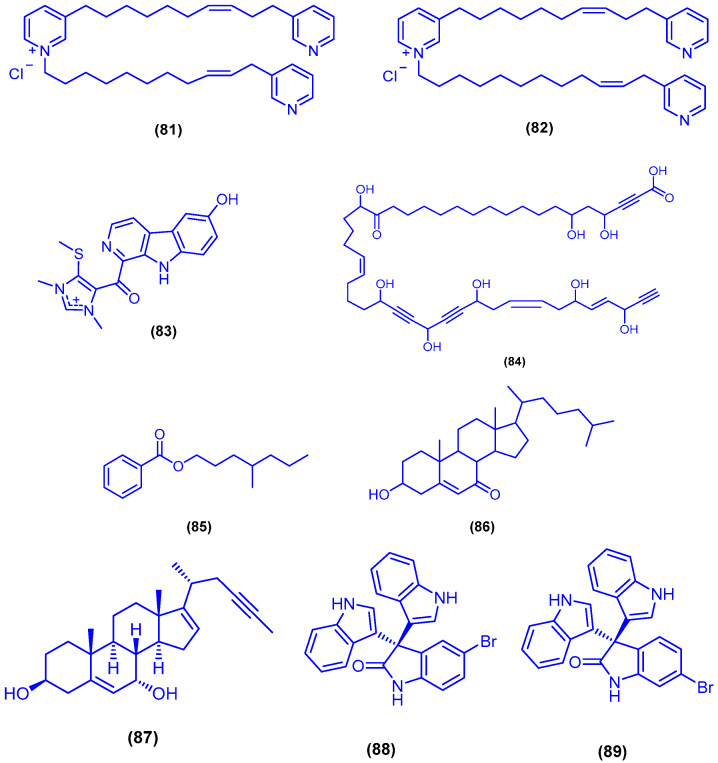
Chemical structures of niphatoxin A (**81**), niphatoxin B (**82**), hyrtiomanzamine (**83**), petrosolic acid (**84**), compounds **85**–**86**, gelliusterol E (**87**), compounds **88** and **89**.

The two tripyridine alkaloids niphatoxin A (**81**) and niphatoxin B (**82**) were isolated from the sponge *Niphates* sp. (Figure 15). Compounds **81** and **82** showed cytotoxic activity against P-388 cells, with IC_50_ value of 0.1 µg/mL [94]. The β-carboline alkaloid hyrtiomanzamine (**83**) was identified and isolated from the marine sponge *Hyrtios erecta* (Figure 15). Hyrtiomanzamine (**83**) showed immunosuppressive activity in the B lymphocytes reaction assay with EC_50_ value of 2 µg/mL, with no cytotoxicity against KB cells [95]. The polyacetylene derivative petrosolic acid (**84**) was isolated from the sponge *Petrosia* sp. (Figure 15). Petrosolic acid (**84**) was tested for its inhibitory activity against various activities of HIV-1 RT (RNA-dependent DNA polymerase (RDDP), DNA-dependent DNA polymerase (DDDP) and RNase H functions), and showed inhibitory activity of RDDP and DDDP with IC_50_ values of 1.2 and 5.9 µM, respectively [96]. The alkyl benzoate compound **85** and the oxysterol 3-β-hydroxycholest-5-en-7-one (**86**) were purified from the methylene chloride fraction of the marine sponge *Hyrtios erectus*, collected from the Saudi coast of Jeddah (Figure 15). Compounds **85** and **86** showed cytotoxic activities against MCF-7 cells, with IC_50_ values of 2.4 and 3.8 μM, respectively. Compound **86** displayed also cytotoxicity against HepG 2 cells with IC_50_ value of 1.3 μM [97]. Bioactivity-guided fractionation of the marine sponge *Callyspongia* aff. *Implexa*, collected from Safaga in Egypt, afforded the sterol compound gelliusterol E (**87**) (Figure 15). Compound **87** was tested against *Chlamydia trachomatis*, which is the leading cause of ocular and genital infections, and displayed antichlamydial activity in a dose-dependent manner with IC_50_ value of 2.3 µM [98]. Two new bioactive brominated oxindole alkaloids **88** and **89** were recently isolated from the sponge *Callyspongia siphonella*, collected from Hurghada. The two metabolites 88 and 89 revealed antibacterial activity against *B. subtilis* with MIC value of 4 µg/mL and *S. aureus* (MIC = 8 and 16 µg/mL, respectively). The two brominated oxindole alkaloids **88** and **89** showed also moderate in vitro antitrypanosomal activity and inhibited the biofilm formation of the Gram-negative bacterium *P. aeruginosa*. Both of **88** and **89** exhibited significant cytotoxicity against three cell lines, including HT-29, OVCAR-3 and MM.1S, with IC_50_ values ranging from 9 to 12.5 µM [59].

### 3.2. Corals

**Figure 16:**
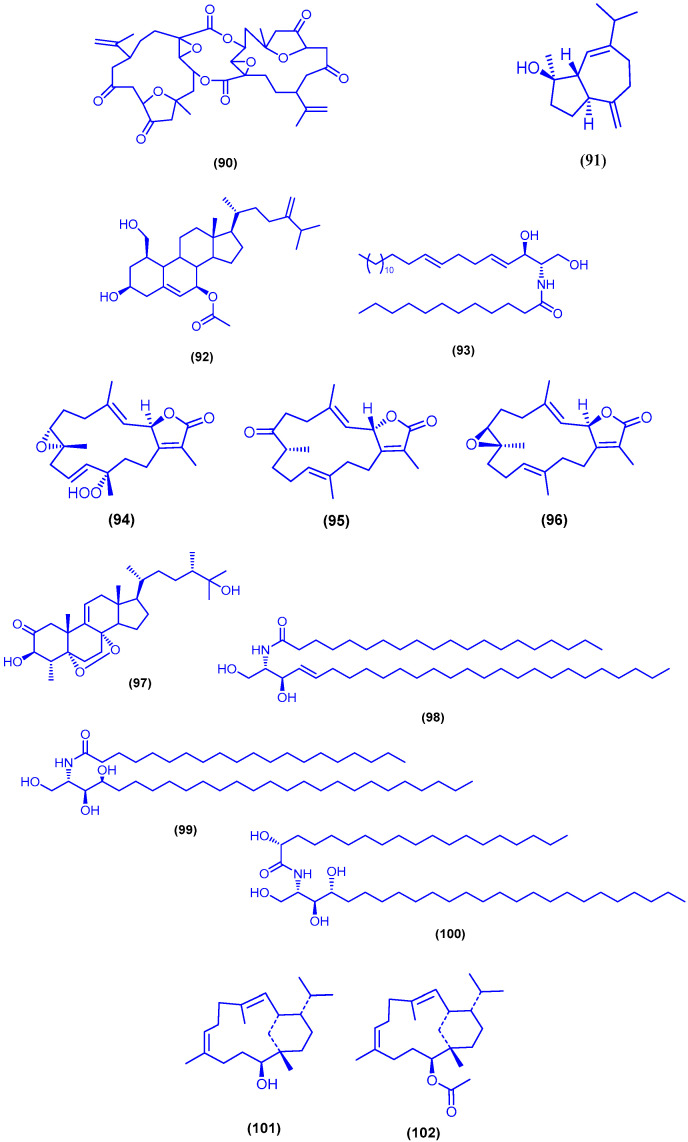
Chemical structure of singardin (**90**), guaianediol (**91**), compound **92** and **93**, 12(*S*)-hydroperoxylsarcoph-10-ene (**94**), 8-*epi*-sarcophinone (**95**), *ent*-sarcophine (**96**), compounds **97**–**100**, sarcotrocheliol (**101**) and sarcotrocheliol acetate (**102**).

The heptacyclic norcembranoid dimer singardin (**90**) and the sesquiterpene guaianediol (**91**) were obtained from the soft coral *Sinularia gardineri* (Pratt) (Alcyoniidae), collected near Hurghada in Egypt (Figure 16). Both compounds exhibited similar cytotoxic activity against murine leukemia (P-388) cells (IC_50_ = 1 μg/mL), human lung carcinoma (A-549) cells (IC_50_ = 2.5 μg/mL), human colon carcinoma (HT-29) cells (IC_50_ = 5 μg/mL) and human melanoma (MEL-28) cells (IC_50_ = 5 μg/mL) [99]. Bioassay-guided fractionation of the soft coral *Litophyton arboretum*, collected from Sharm El-Sheikh, led to the isolation of 7β-acetoxy-24-methylcholesta-5-24(28)-diene-3,19-diol (**92**) and erythro-*N*-dodecanoyl-docosasphinga-(4*E*,8*E*)-dienine (**93**) (Figure 16). Compounds **92** and **93** displayed potent inhibitory activity against HIV-1 PR with IC_50_ values of 4.85 and 4.8 µM, respectively. Compound **92** exhibited also cytotoxicity against HeLa cells at CC_50_ value of 4.3 µM with selectivity index of 8.1, while compound **93** did not show cytotoxic activity [100]. Chemical investigations of the soft coral *Sarcophyton glaucum*, collected from Hurghada, afforded the peroxide diterpene 12(*S*)-hydroperoxylsarcoph-10-ene (**94**), together with 8-*epi*-sarcophinone (**95**) and *ent*-sarcophine (**96**) (Figure 16) [101]. Compounds **95** and **96** were also isolated from the soft coral *Sarcophyton trocheliophorum*, collected from the Hurghada [102]. Compounds **94**–**96** were inhibitors of the phase I enzyme cytochrome P450 1A with IC_50_ values of 2.7, 3.7 and 3.4 nM, respectively [101]. The polyhydroxylated sterol 97 together with the three ceramides 98–100 were isolated from the soft coral *Sinularia candidula*, collected from the Egyptian coast of Safaga (Figure 16). Compounds **97**–**100** exhibited antiviral activity against H5N1 virus and reduced the virus titer, at a concentration of 1 ng/mL, by 55.16%, 48.81%, 10.43% and 15.76%, respectively [103]. Fractionation of the organic extract of the soft coral *Sarcophyton glaucum*, collected from north of Jeddah in Saudi Arabia, yielded the two cembranoids sarcotrocheliol (**101**) and sarcotrocheliol acetate (**102**) (Figure 16). Sarcotrocheliol (**101**) and its acetate derivative 102 showed cytotoxic activity against MCF-7 cells with IC_50_ values of 2.4 and 3.2 µM, respectively [104]. 

### 3.3. Sea Hares

**Figure 17:**
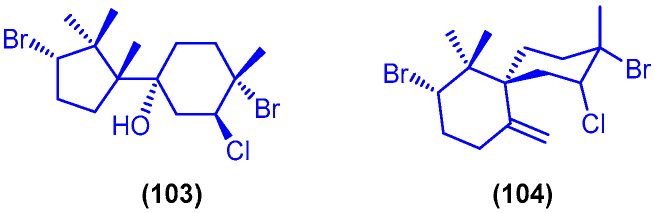
Chemical structures of oculiferane (**103**) and *epi*-obtusane (**104**).

Oculiferane (**103**) and *epi*-obtusane (**104**) are two sesquiterpenes isolated from the acetone extract of the digestive gland of the sea hare *Aplysia oculifera*, which was collected from 40 km south of Safaga, Egypt (Figure 17). Compounds **103** and **104** showed in vitro cytotoxicity against several human cancer cell lines, including prostate carcinoma cells (PC-3), lung carcinoma (A549), human breast adenocarcinoma (MCF-7), hepatocellular carcinoma (HepG2) and colorectal carcinoma (HCT 116) cells, with a range of IC_50_ values ranging from 0.96 to 5.9 µg/mL [105]. Isolation of dolastatin 16 (**4**) (Figure 5) from the sea hare *Dolabella auricularia* and its evaluation as anticancer agent were also reported. Compound **4** showed anticancer activity against several human cancer cell lines at a range of low micromolar concentrations (GI_50_ = 0.0012–0.00096 μg/mL) [106].

### 3.4. Tunicates

**Figure 18:**
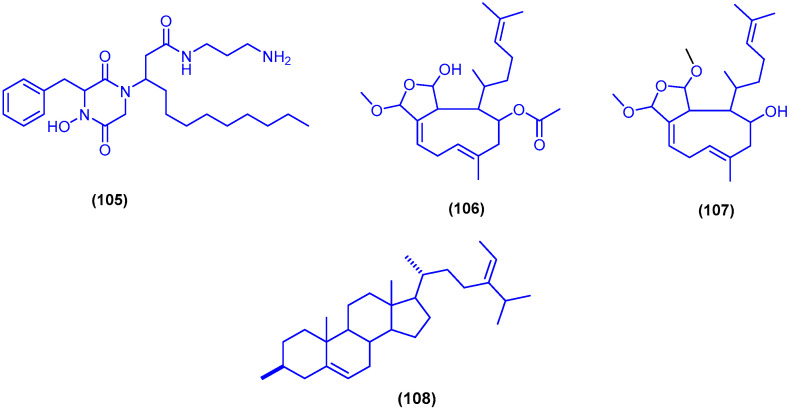
Chemical structures of etzionin (**105**), compound **106** and **107** and fucosterol (**108**).

The diketopiperazine hydroxamate derivative etzionin (**105**) was isolated from an unidentified red tunicate, collected in the Northern part of the Gulf of Eilat in Israel (Figure 18). Etzionin (**105**) exhibited antifungal activity against the pathogenic yeast *Candida albicans* with MIC value of 3 µg/mL, in RPMI-1640 broth [107].

## 4. Marine Natural Products Isolated from Marine Algae

Two cytotoxic xenicane diterpenes, 18,19-epoxyxenic-19-methoxy-18-hydroxy-4-acetoxy-6,9,13-triene (**106**) and 18,19-epoxyxenic-18,19-dimethoxy-4-hydroxy-6,9,13-triene (**107**), were purified from the methanol extract of the brown alga *Padina pavonia* (L.) Gaill., which was obtained from Hurghada, Egypt (Figure 18). The two cytotoxic xenicane diterpenes 106 and 107 showed significant in vitro cytotoxic activity against lung carcinoma (H460) cells at concentrations of 1–5 μg/mL [108]. The steroidal compound fucosterol (**108**) was isolated from the alcoholic extract of the brown alga *Dictyota dichotoma* (Hudson) Lamouroux, which was collected from Hurgada (Figure 18) [109]. Compound **108** was also isolated from the brown alga *Dictyota dichotoma* (Huds) Lamour, collected at Ras Abu-Bakr, 65 km north of Ras Gharib on Suez-Gulf, Egypt [110]. Compound **108** displayed potent cytotoxicity against mouse P 388 leukemia cells with IC_50_ value of 0.6 µg/mL [109,111].

## 5. Sea Grasses

**Figure 19:**
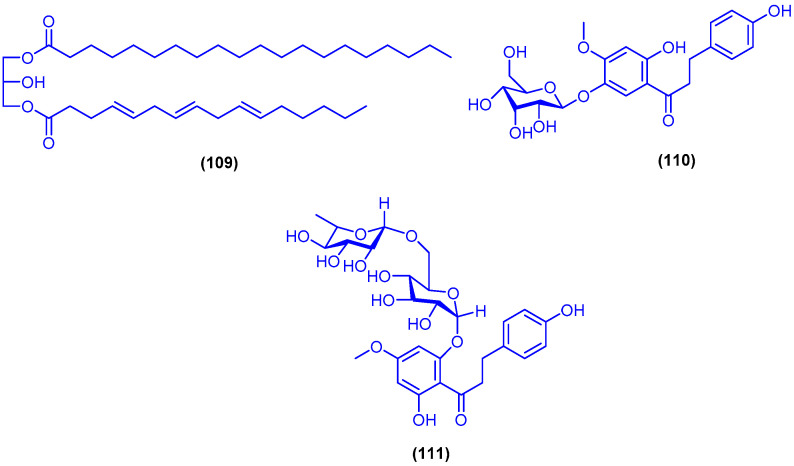
Chemical structures of compound **109**, asebotin (**110**) and thalassodendrone (**111**).

Bioactivity-guided fractionation of the extracts of the sea grass *Thallasodendron ciliatum*, collected from Egypt in Safaga [112] and Magawish near to Hurghada [113] led to the isolation of the diglyceride ester 109 and asebotin (**110**) (Figure 19). Anti-H5N1 virus activity of compounds **109** and **110** was measured using plaque inhibition assay in Madin–Darby canine kidney. Compounds **109** and **110** displayed reduction of virus titer by 67.26% and 53.81% inhibition at concentration of 1 ng/mL, respectively [112]. In a further study, the dihydrochalcone diglycoside thalassodendrone (**111**) together with asebotin (**110**) have been isolated from the ethyl acetate fraction of the sea grass *Thalassodendrin ciliatum* (Forsk.), which was obtained from Magawish city near Hurghada in Egypt (Figure 19). Asebotin (**110**) and thalassodendrone (**111**) exhibited antiviral activity against influenza A virus with IC_50_ values of 2 and 1.96 µg/mL, respectively, and with cytotoxic concentrations (CC_50_) of 3.36 and 3.14 µg/mL, respectively [114].

## 6. Genomic Potential of Red Sea Organisms

Metagenomic approach plays a crucial role in evaluating biodiversity from highly diverse and/or extreme habitats, e.g., marine environments, through direct access to genomes of inhabitant organisms. This has been potentiated through the integration of high-throughput DNA sequencing technologies and advanced-bioinformatic analyses, which highlighted the importance of metagenomics in bioprospecting novel bioactive metabolites from marine sources and their potential applications in biotechnology [115]. Metagenomic studies revealed that Red Sea harbors a unique microbial community with unique genes, enzymes and biosynthetic pathways, compared to other marine environments. This could be explained by the fact that Red Sea is regarded as an unusual ecosystem, characterized by its high salinity, high temperature, high ultra-violet radiation and low nutrients, as well as the existence of vibrant coral reefs and more than 25 hot brine pools [116]. In 2016, a group of researchers provided an overview of the previous metagenomic studies that were performed in the Red Sea [117]. Examination of the microbial community in the sediments of Kebrit Deep brine pool using 16srRNA gene PCR amplification, proved the presence of 16S sequences belonging to bacteria and Archaea unavailable in databases, suggesting the presence of novel microbial species harbored in Red Sea [118]. Further investigation of other brine pools in the Red Sea, gave accurate understanding about the microbial community composition and their unique metabolic pathways associated with the adaptation in such extreme environmental conditions [4,119]. 

The Red Sea coral *Stylophora pistillata* was found to live in symbiotic relationship with *Endozoicomonas* bacteria, that play a major role in the coral defense, health and survival. Studies demonstrated that diseased corals had lost their *Endozoicomonas* and has harbored opportunistic pathogens, including Alternaria and *Achromobacter*. Similarly, the symbiotic relationship between sponges, such as *Theonella swinhoei*, and their microbiomes had essentially contributed in the adaptation of the sponge to drastic environmental conditions and great tolerance to arsenite and arsenate [120]. Studies also revealed that the symbiotic association between the host and microbe can influence the microbial symbionts evolution. For instance, one of the unculturable sponge symbionts, the *cyanobaterium Candidatus Synechococcus spongiarum* associated with the Red Sea sponge *Carteriospongia foliascens*, has lost partial genes encoding proteins responsible for photosynthesis, production of polysaccharides, DNA repair mechanism and environmental stress adaptation, when compared to free-living cyanobacterial strains. The sponge tissues were collected from site RB4 (22°44′56′′ N, 38°59′35′′ E), located in the Rabigh Bay of Saudi Arabia along the Red Sea coast. Metagenomic DNA was sequenced on a 454 FLX platform utilizing Titanium chemistry, which produced a total of 315, 119 reads with a total length of 160.2 Mbp. Genome database were searched against the Kyoto Encyclopedia of Genes and Genomes (KEGG) database by using Basic Local Alignment Search Tool for Protein (BLASTP). Amino acid sequences were also searched against the GenBank NR data-base, and the output xml file was imported into MEGAN for taxonomic affiliation and SEED/Subsystems annotation. For the phylogenomic analysis, 31 proteins encoding phylogenetic markers were predicted from the SH4 draft genome and cyanobacterial genomes in the JGI database using AMPHORA. The sequences of each marker gene were aligned individually using ClustalW. The aligned sequences were concatenated, and a maximum-likelihood phylogenetic tree was constructed using PhyML [121].

The genome of Euryarchaea *Halorhabdus tiamatea*, obtained from the Shaban deep-sea hypersaline anoxic brine pool in the Red Sea, was sequenced by Werner et al. [122] in order to elucidate its niche adaptations. The study demonstrated that among the sequenced archaea, *H. tiamatea* enclosed the highest number of genes encoding glycoside hydrolases that could be potentially applied in industry. Glycosidase activity measurements suggested an adaptation towards recalcitrant algal and plant-derived hemicelluloses. Furthermore, *H. tiamatea* encoded proteins characteristic for thermophiles and light-dependent enzymes (e.g., bacteriorhodopsin), suggesting that *H. tiamatea* evolution was mostly not governed by a dark, cold, anoxic deep-sea habitat. The results supported that Halorhabdus species can occupy a distinct niche as polysaccharide degraders in hypersaline environments. *H. tiamatea* was sequenced on a 454 FLX Ti sequencer. Ribosomal RNA genes were identified via BLAST searches against public nucleotide databases and transfer RNA genes using TRNASCAN-SE v. 1.21. Selected CAZymes, from multi-functional CAZyme families, were subjected to in-depth phylogenetic. For each family, a set of experimentally characterized proteins was selected and aligned with their *H. tiamatea* homologues using MAFFT with iterative refinement and the Blosum62 matrix. Phylogenetic trees were computed from these alignments using PHYML.

Mohamed et al. [123] reported the largest number of Red Sea microbial genomes in a single study, with the aim to understand the microbial adaptation strategies to the extreme environmental conditions in Red Sea and allow the bioprospecting for novel thermo-and/or halo-philic enzymes. They have reported 136 microbial genomes assembled from 45 metagenomes, sampled form multiple depths (10–500 m), gradients in salinity, temperature, nutrients and oxygen, from 8 stations along the Red Sea. This represented great variation in environmental conditions as well as microbial diversity. The 136 retrieved genomes belonged to seven different phyla: *Thaumarchaeota, Euryarchaeota*, Actinobacteria, Cyanobacteria, *Bdellovibrionaeota*, Proteobacteria, and Marinimicrobia. Genomic DNA was extracted then sequenced on a HiSeq 2000 (Illumina, San Diego, CA, USA). Reads were quality checked and trimmed using PRINSEQ v0.20.4 generating read lengths of ~93 bp and a total of ~10 million reads per sample. Trimmed metagenome reads were individually assembled using IDBA-UD v1.1.1. The trimmed reads were mapped back to contigs using BWA v0.7.12 with the bwa-mem algorithm.

Ryu and coworkers [124] analyzed the hologenome data including genome, transcriptome, and metatranscriptome of two marine sponges; *Stylissa carteri* and *Xestospongia testudinaria* in order to investigate the host-symbiont interaction. Both species were collected at Fsar Reef (22.228408 N, 39.028187 E) on the Red Sea coast of Saudi Arabia, at a depth of 13–14 m. The paired-end and mate-pair sequencing was conducted using HiSeq2000 technology (Illumina), 454 and Ion proton sequencing were also performed using standard protocols. The Markov Cluster (MCL) Algorithm was utilized to cluster the Scavenger Receptor Cysteine-Rich (SRCR)-like domains together with the application of Blastp bit score as a similarity metric. The peptide sequences of the SRCR-like domain from each cluster were aligned using MAFFT v7. 123b and a custom Python script was used to compute the amino acid frequency at each aligned position. This is considered as a valuable and unique study because *S. carteri* is the first species in the order Halichondrida to have its genome sequenced whereas *X. testudinaria* is the first HMA sponge to have its genome sequenced. Results showed that *S. carteri*, when compared with *X. testudinaria*, has an expanded repertoire of immunological domains, in particular SRCR-like domains. Furthermore, an over-representation of potential symbiosis-related domains in *X. testudinari* was revealed through the metatranscriptome analyses.

The Red Sea sponge *Theonella swinhoei* was studied in order to investigate the uncultured majority of its symbionts. The sponge was collected from the Gulf of Aqaba, near Eilat. The DNA of *T. swinhoei* microbiome was sheared (200- to 400-bp size) and libraries were generated using the TruSeq DNA standard protocol and pooled for sequencing on one lane of the Illumina HiSeq 2000 platform. This study identified a complete *N*-acyl-homoserine lactone (AHL)-QS system (designated TswIR) and seven partial luxI homologues in the microbiome of *T. swinhoei*. The TswIR system was novel and shown to be associated with an alphaproteobacterium belonging to the order Rhodobacterales (termed Rhodobacterales bacterium TS309). When the gene tswI was expressed in Escherichia coli, it produced three AHLs, two of which were also identified in the studied sponge extract. The presence of some sponge-specific characteristics, such as ankyrin-like domains and tetratricopeptide repeats, the taxonomic affiliation of the 16S rRNA of Rhodobacterales bacterium TS309 to a sponge-coral specific clade, and its enrichment in sponge versus seawater and marine sediment samples, proved a likely symbiotic nature of this bacterium [125].

Recently, another group of researchers [126] focused on the isolation of *Pseudomonas* sp. associated with marine sponge *Hyrtios aff. erectus*, phylogenetic identification and molecular screening of their metabolic pathways polyketide synthases and nonribosomal peptides (PSK and NRPs). The sponge samples were obtained from the Red Sea at Hurghada city. The 16S rRNA gene sequencing of bacterial isolate demonstrated that active metabolic pathways genes in the *Pseudomonas* sp. was PSK II, which could be the reason for the antioxidant and cytotoxic bioactivity of the bacteria. The study, including the antioxidant, cytotoxicity and bioactive metabolic screening pathways, confirmed that this bacterial strain was shown to be an important source of natural antioxidant and cytotoxic metabolites against free radical and cancerous cell line.

## 7. Conclusions

**Figure 20:**
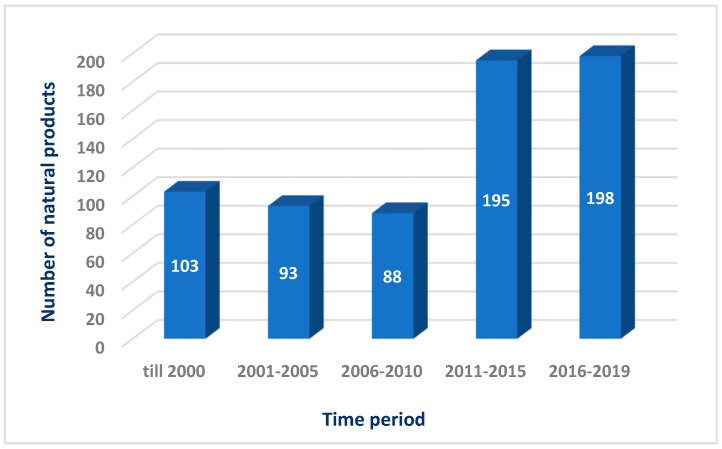
Numbers of natural products isolated from the Red Sea marine organisms.

**Figure 21:**
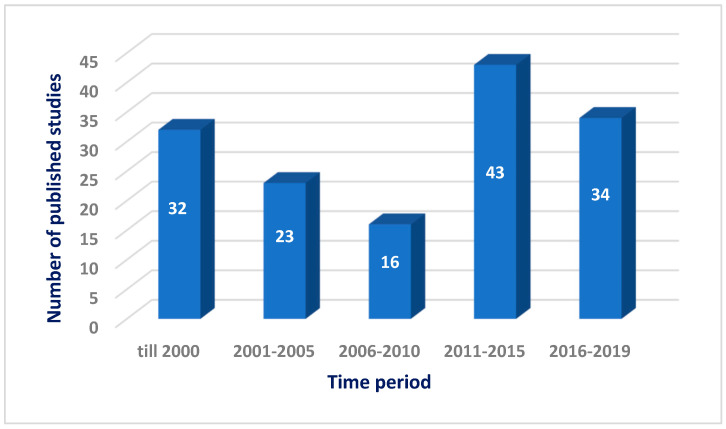
Numbers of published studies on natural products isolated from the Red Sea marine organisms.

**Figure 22:**
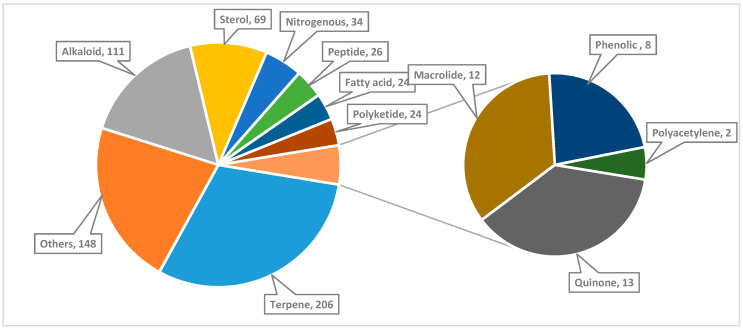
Chemical classes of natural products isolated from the Red Sea marine organisms.

The Red Sea is a rich and diverse ecosystem with 2000 km of coral reef extending along its coastline. It is inhabited by over 1000 invertebrate species, and 200 soft and hard corals. Due to this high biodiversity and limited research, the Red Sea is a promising underexplored habitat for the discovery of new bioactive marine natural products. Out of these, 677 natural products came from organisms isolated from the Red Sea, with almost 60% of these compounds coming from reports over the last decade (2011–2019) (Figure 20 and Figure 21). This significant increase of reported compounds highlights the enormous potential of marine organisms, particularly from the Red Sea. Furthermore, the diversity of these chemical structures is quite remarkable. Majority of these compounds are represented by terpenes but also includes other classes namely alkaloids, sterols, peptides and other nitrogenous compounds, polyketides, fatty acids, macrolides, quinones, polyacetylene and flavonoids (Figure 22). It is interesting to note that from these different classes of natural products, majority of the compounds that were reported to exhibit the highest proportion of biological activities are terpenes followed by alkaloids. The presented marine natural products in this review (compounds **1**–**111**) exhibited a wide range of remarkable and potent biological activities, including for example, antioxidant, anticonvulsant, anticancer and anti-infective activities. However, biologically active marine natural products must be non-cytotoxic on normal cells, to be suitable for using as drugs. In the published studies, some of these biologically active marine natural products were tested for their cytotoxicity and found to be not toxic on normal cells. Therefore, these marine natural products could be promising compounds for developing new drugs after further investigations. Other biologically active marine natural products, which were not tested for their cytotoxicity, demand a testing for their cytotoxicity. Some of the illustrated cytotoxic marine natural products have PAINS characteristics (Pan-assay interference compounds), which could explain their cytotoxic activity. Among the organisms collected from the Red Sea, marine sponges remain to be the most widely sampled (Figure 3). This is not surprising since diverse species of marine invertebrates particularly sponges have been collected from the Red Sea and globally. Gram positive bacteria such as actinomycetes as well as fungi isolated from various marine organisms from the Red Sea have also been shown to be the prolific producers of these bioactive natural products (Figure 3). Furthermore, majority of the reported marine organisms have been collected from Egypt and Saudi Arabia coasts, accounting for 58% and 16% respectively. In contrast, less than 1% were isolated from Dijibouti, Jordan and Yemen (Figure 2). This could be attributed to the high biodiversity of organisms in Egyptian environment in comparison to other locations. Moreover, more research should be directed to explore other environments. 

## Supplementary Materials

The following are available online at https://www.mdpi.com/1660-3397/18/9/457/s1. Table S1, Marine natural products of the Red Sea; Table S2: Locations of collection of the Red Sea marine organisms; Table S3: Taxonomy of marine organisms collected from the Red Sea.
