# Supplementary material for: Natural Products Repertoire of the Red Sea
Source: Mar Drugs. 2020 Sep 4;18(9):457. doi: 10.3390/md18090457 (PMC7551641; doi:10.3390/md18090457)
Supplement: Supplementary file 1 [file marinedrugs-18-00457-s001.pdf]

Natural Products Repertoire of the Red Sea

Ebaa M. El-Hossary [1], Mohammad Abdel-Halim [2], Eslam S. Ibrahim [3,4], Sheila Marie Pimentel-Elardo [5], Justin R. Nodwell [5], Heba Handoussa [6], Miada F. Abdelwahab [7], Ulrike Holzgrabe [8,*], Usama Ramadan Abdelmohsen [7,9,*]

[1] National Centre for Radiation Research & Technology, Egyptian Atomic Energy Authority, Ahmed El-Zomor St. 3, El-Zohoor Dist., Nasr City, 11765 Cairo, Egypt

[2] Department of Pharmaceutical Chemistry, Faculty of Pharmacy and Biotechnology, German University in Cairo, 11835 Cairo, Egypt

[3] Department of Microbiology and Immunology, Faculty of Pharmacy, Cairo University, 11562 Cairo, Egypt

[4] Institute for Molecular Infection Biology, University of Würzburg, Josef-Schneider-Strasse 2/Bau D15, 97080 Würzburg, Germany

[5] Department of Biochemistry, University of Toronto, MaRS Centre West, 661 University Avenue, Toronto, ON, M5G 1M1, Canada

[6] Department of Pharmaceutical Biology, Faculty of Pharmacy and Biotechnology, German University in Cairo, 11835 Cairo, Egypt

[7] Department of Pharmacognosy, Faculty of Pharmacy, Minia University, 61519 Minia, Egypt

[8] Institute for Pharmacy and Food Chemistry, University of Würzburg, Am Hubland, 97074 Würzburg, Germany

[9] Department of Pharmacognosy, Faculty of Pharmacy, Deraya University, Universities Zone, P.O. Box 61111 New Minia City, 61519 Minia, Egypt

---

[*] **Corresponding authors:**

Email addresses: Ulrike Holzgrabe (ulrike.holzgrabe@uni-wuerzburg.de) and Usama Ramadan Abdelmohsen (usama.ramadan@mu.edu.eg)

Table S1: Marine natural products of the Red Sea

No.	Compound	Class	Source (Marine bacteria)	Biological Activity	Ref
**112**	Curacin D	Nitrogenous	Cyanobacterium *Moorea producens*	Not mentioned	[1]
**113**	6-(sec-butyl)-3-isopropylpyrazin-2(1H)-one	Alkaloid	Tunicate-derived actinomycete, *Streptomyces* sp. Did-27	Cytotoxic effects against human colorectal carcinoma HCT-116, human liver cancer HepG2, and human breast cancer MCF-7 (IC_{50}= 30, ≥ 50, and 25 µM, respectively)	[2]
**114**	3-(sec-butyl)-6-isopropylpyrazin- 2(1H)-one	Alkaloid	Tunicate-derived actinomycete, *Streptomyces* sp. Did-27	Not mentioned	
**115**	6-(sec-butyl)-3-isobutylpyrazin-2(1H)-one	Alkaloid	Tunicate-derived actinomycete, *Streptomyces* sp. Did-27	Cytotoxic effects against human colorectal carcinoma HCT-116, human liver cancer HepG2, and human breast cancer MCF-7 (IC_{50} = 30, ≥ 50, and 35 µM, respectively)	
**116**	Deoxymuta aspergillic acid	Alkaloid	Tunicate-derived actinomycete, *Streptomyces* sp. Did-27	Cytotoxic effects against human colorectal carcinoma HCT-116, human liver cancer HepG2, and human breast cancer MCF-7 (IC_{50} = 35, ≥ 50, and 20 µM, respectively)	
**117**	3,6-Di-sec-butyl-2(1H)-pyrazinone	Alkaloid	Tunicate-derived actinomycete, *Streptomyces* sp. Did-27	Cytotoxic effects against human colorectal carcinoma HCT-116, human liver cancer HepG2, and human breast cancer MCF-7 (IC_{50} = 18, ≥ 50, and 10 µM, respectively)	
**118**	Cyclo (6-OH-D-Pro-L-Phe)	Alkaloid	Tunicate-derived actinomycete, *Streptomyces* sp. Did-27	Cytotoxic effects against human colorectal carcinoma HCT-116, human liver cancer HepG2, and human breast cancer MCF-7 (IC_{50} = 30, ≥ 50, and 30 µM, respectively)	
**119**	Bacillusamide B	Alkaloid	Tunicate-derived actinomycete, *Streptomyces* sp. Did-27	Cytotoxic effects against human colorectal carcinoma HCT-116, human liver cancer HepG2, and human breast cancer MCF-7 (IC_{50} = 25, ≥ 50, and 27 µM, respectively)	
**120**	Cyclo (L-Pro-L-Leu)	Alkaloid	Tunicate-derived actinomycete, *Streptomyces* sp. Did-27	Cytotoxic effects against human colorectal carcinoma HCT-116, human liver cancer HepG2, and human breast cancer MCF-7 (IC_{50} =16, ≥ 50, and 30 µM, respectively)	

No.	Compound	Class	Source (Marine bacteria)	Biological Activity	Ref
**121**	Cyclo (L-Pro-L-Ile)	Alkaloid	Tunicate-derived actinomycete, *Streptomyces* sp. Did-27	Cytotoxic effects against human colorectal carcinoma HCT-116, human liver cancer HepG2, and human breast cancer MCF-7 (IC_{50} = 22, ≥ 50, and 27 µM, respectively)	
**122**	2,3-Seco-2,3-dioxo-lyngbyatoxin A	Alkaloid	Cyanobacterium *Moorea producens*	Antiproliferative activity against human HeLa cervix carcinoma (IC_{50} > 50 µM)	[3]
**123**	Majusculamides A	Peptide	Cyanobacterium *Moorea producens*	Antiproliferative activity against human cervix carcinoma HeLa cell line (IC_{50} > 50 µM)	
**124**	Majusculamides B	Peptide	Cyanobacterium *Moorea producens*	Antiproliferative activity against human cervix carcinoma HeLa cell line (IC_{50} > 50 µM)	
**125**	Aqabamycin A	Nitrogenous	*Vibrio* sp., isolated from the surface of the soft coral *Sinularia polydactyla*	Antimicrobial activity against *B. subtilis, Micrococcus luteus, E. coli, Proteus vulgaris* at MIC = 50, 50, 100 and 50 µg/ml, respectively<br>Antifungal activity against *C. albicans, Magnaporthe grisea, Mucor miehe, Nematospora coryli, Paecilomyces variotii, Phytophthora infestans, Saccharomyces cerevisiae, Ustilago nuda* at MIC = > 100, > 100, > 100, 10, 50, > 100, > 100 and > 100 µg/ml, respectively	[4]
**126**	Aqabamycin B	Nitrogenous	*Vibrio* sp., isolated from the surface of the soft coral *Sinularia polydactyla*	Antimicrobial activity against *B. subtilis, Micrococcus luteus, E. coli, Proteus vulgaris* at MIC = 100, 100, 100, 100 µg/ml, respectively<br>Antifungal activity against *C. albicans, Magnaporthe grisea, Mucor miehe, Nematospora coryli, Paecilomyces variotii, Phytophthora infestans, Saccharomyces cerevisiae, Ustilago nuda* at MIC = > 100, > 100, > 100, 100, > 100, > 100, > 100 and> 100 µg/ml, respectively	
**127**	Aqabamycin C	Nitrogenous	*Vibrio* sp., isolated from the surface of the soft coral *Sinularia polydactyla*	Antimicrobial activity against *B. subtilis, Micrococcus luteus, E. coli, Proteus vulgaris* at MIC = 25, 25, 50 and 12.5 µg/ml, respectively<br>Antifungal activity against *C. albicans, Magnaporthe grisea, Mucor miehe, Nematospora coryli, Paecilomyces variotii, Phytophthora infestans, Saccharomyces cerevisiae, Ustilago nuda* at MIC = 100, 50, 50, 50, 50, 100, 100 and 50 µg/ml, respectively	

No.	Compound	Class	Source (Marine bacteria)	Biological Activity	Ref
**128**	Aqabamycin D	Nitrogenous	*Vibrio* sp., isolated from the surface of the soft coral *Sinularia polydactyla*	Antimicrobial activity against *B. subtilis, Micrococcus luteus, E. coli, Proteus vulgaris* at MIC = 50, 100, 100 and 50 µg/ml, respectively<br>Antifungal activity against *C. albicans, Magnaporthe grisea, Mucor miehe, Nematospora coryli, Paecilomyces variotii, Phytophthora infestans, Saccharomyces cerevisiae, Ustilago nuda* at MIC = > 100, > 100, > 100, 100, > 100, > 100, > 100 and > 100 µg/ml, respectively	
**129**	Aqabamycin E	Nitrogenous	*Vibrio* sp., isolated from the surface of the soft coral *Sinularia polydactyla*	Antimicrobial activity against *B. subtilis, Micrococcus luteus, E. coli,* at MIC = 6.25, 6.25 and 12.5 µg/ml, respectively<br>Antifungal activity against *C. albicans, Magnaporthe grisea, Mucor miehe, Nematospora coryli, Paecilomyces variotii, Phytophthora infestans, Saccharomyces cerevisiae, Ustilago nuda* at MIC = > 100, 50, 50, 50, 50, > 100, > 100 and > 100 µg/ml, respectively	
**130**	Aqabamycin F	Nitrogenous	*Vibrio* sp., isolated from the surface of the soft coral *Sinularia polydactyla*	Antimicrobial activity against *B. subtilis, Micrococcus luteus, E. coli, Proteus vulgaris* at MIC = 12.5, 12.5, 12.5 and 25 µg/ml, respectively<br>Antifungal activity against *C. albicans, Magnaporthe grisea, Mucor miehe, Nematospora coryli, Paecilomyces variotii, Phytophthora infestans, Saccharomyces cerevisiae, Ustilago nuda* at MIC = > 100, > 100, > 100, 100, > 100, > 100, > 100 and > 100 µg/ml, respectively	
**131**	Aqabamycin G	Nitrogenous	*Vibrio* sp., isolated from the surface of the soft coral *Sinularia polydactyla*	Antimicrobial activity against *B. subtilis, Micrococcus luteus, E. coli, Proteus vulgaris* at MIC = 25, 25, 50 and 25 µg/ml, respectively<br>Antifungal activity against *C. albicans, Magnaporthe grisea, Mucor miehe, Nematospora coryli, Paecilomyces variotii, Phytophthora infestans, Saccharomyces cerevisiae, Ustilago nuda* at MIC = > 100, > 100, 100, 50, > 100, > 100, > 100 and > 100 µg/ml, respectively	

No.	Compound	Class	Source (Marine bacteria)	Biological Activity	Ref
**132**	3-Nitro-1*H*-indazole	Alkaloid	*Vibrio* sp., isolated from the surface of the soft coral Sinularia polydactyla	Antimicrobial activity against *B. subtilis, Micrococcus luteus, E. coli, Proteus vulgaris* at MIC = > 100, > 100, > 100 and > 100 µg/ml, respectively<br>Antifungal activity against *C. albicans, Magnaporthe grisea, Mucor miehe, Nematospora coryli, Paecilomyces variotii, Phytophthora infestans, Saccharomyces cerevisiae, Ustilago nuda* at MIC = > 100, > 100, > 100, > 100, > 100, > 100, > 100 and > 100 µg/ml, respectively	
**133**	Indazole-3-carbaldehyde	Alkaloid	*Vibrio* sp., isolated from the surface of the soft coral *Sinularia polydactyla*	Antimicrobial activity against *B. subtilis, Micrococcus luteus, E. coli, Proteus vulgaris* at MIC = 100, >100, > 100 and > 100 µg/ml, respectively<br>Antifungal activity against *C. albicans, Magnaporthe grisea, Mucor miehe, Nematospora coryli, Paecilomyces variotii, Phytophthora infestans, Saccharomyces cerevisiae, Ustilago nuda* at MIC = >100, >100, >100, 50, >100, > 100, >100 and >100 µg/ml, respectively	
**134**	Benzoic acid	Others	*Vibrio* sp., isolated from the surface of the soft coral *Sinularia polydactyla*	Not mentioned	
**135**	4-Hydroxycinnamic acid	Others	*Vibrio* sp., isolated from the surface of the soft coral *Sinularia polydactyla*	Not mentioned	
**136**	3-(3-Nitro-4-hydroxyphenyl)-2-propenoic acid	Others	*Vibrio* sp., isolated from the surface of the soft coral *Sinularia polydactyla*	Antimicrobial activity against *B. subtilis, Micrococcus luteus, E. coli, Proteus vulgaris* at MIC = 100, >100, > 100 and >100 µg/ml, respectively<br>Antifungal activity against *C. albicans, Magnaporthe grisea, Mucor miehe, Nematospora coryli, Paecilomyces variotii, Phytophthora infestans, Saccharomyces cerevisiae, Ustilago nuda* at MIC = >100, 25, >100, 50, >100, > 100, >100 and >100 µg/ml, respectively	

No.	Compound	Class	Source (Marine bacteria)	Biological Activity	Ref
**137**	3-Nitro-4-hydroxybenzaldehyde	Others	*Vibrio* sp., isolated from the surface of the soft coral *Sinularia polydactyla*	Antimicrobial activity against *B. subtilis, Micrococcus luteus, E. coli, Proteus vulgaris* at MIC = 25, 25, 50 and 25 µg/ml, respectively<br>Antifungal activity against *C. albicans, Magnaporthe grisea, Mucor miehe, Nematospora coryli, Paecilomyces variotii, Phytophthora infestans, Saccharomyces cerevisiae, Ustilago nuda* at MIC = >100, 5, 50, 50, 100, 50, 100 and 50 µg/ml, respectively	
**138**	Phenyl-2-bis-indolylmethane	Alkaloid	*Vibrio* sp., isolated from the surface of the soft coral *Sinularia polydactyla*	Not mentioned	
**139**	Turbomycin B	Alkaloid	*Vibrio* sp., isolated from the surface of the soft coral *Sinularia polydactyla*	Not mentioned	
**140**	Vibrindole A	Alkaloid	*Vibrio* sp., isolated from the surface of the soft coral *Sinularia polydactyla*	Not mentioned	
**141**	Phenylacetic acid	Others	*Vibrio* sp., isolated from the surface of the soft coral *Sinularia polydactyla*	Not mentioned	
**142**	3-Hydroxybenzoic acid	Others	*Vibrio* sp., isolated from the surface of the soft coral *Sinularia polydactyla*	Not mentioned	
**143**	1,4-Dithiane	Others	*Vibrio* sp., isolated from the surface of the soft coral *Sinularia polydactyla*	Not mentioned	
**144**	Malyngamide 4	Peptide	Cyanobacterium *Moorea producens*	Cytotoxic effects against human breast adenocarcinoma cell MDA-MB-231, human lung carcinoma A549, and human colorectal carcinoma HT29 (IC_{50} = 44, 40, and 50 µM, respectively)	[5]
**145**	Malyngamide A	Peptide	Cyanobacterium *Moorea producens*	Cytotoxic effects against human breast adenocarcinoma cell MDA-MB-231, human lung carcinoma A549, and human colorectal carcinoma HT29 (IC_{50} = 75, 88, and 70 µM, respectively)	
**146**	Malyngamide B	Peptide	Cyanobacterium *Moorea producens*	Cytotoxic effects against human breast adenocarcinoma cell MDA-MB-231, human lung carcinoma A549, and human colorectal carcinoma HT29 (IC_{50} = 52, 45, and 60 µM, respectively)	

No.	Compound	Class	Source (Marine bacteria)	Biological Activity	Ref
**147**	Lyngbic acid	Fatty acid	Cyanobacterium *Moorea producens*	Cytotoxic effects against human breast adenocarcinoma cell MDA-MB-231, human lung carcinoma A549, and human colorectal carcinoma HT29 (IC_{50}= 65, 66, and 63 µM, respectively)	
**148**	Aplysiatoxin	Polyketide	Cyanobacterium *Moorea producens*	Antiproliferative activity against human HeLa cervix carcinoma (IC_{50}= 13.3 µM)	
**149**	Mooreaside A	Others	Cyanobacterium *Moorea producens*	Cytotoxic effects against human colorectal carcinoma HCT-116, human liver cancer HepG2, and human breast cancer MCF-7 (IC_{50}values of > 50,> 50, and 20.5 µM, respectively)	[6]
**150**	3-Acetyl-2'-deoxyuridine	Others	Cyanobacterium *Moorea producens*	Cytotoxic effects against human colorectal carcinoma HCT-116, human liver cancer HepG2, and human breast cancer MCF-7 (IC_{50}values of > 50,> 50, and 18.2 µM, respectively)	
**151**	3-Phenylethyl-2'-deoxyuridine	Others	Cyanobacterium *Moorea producens*	Cytotoxic effects against human colorectal carcinoma HCT-116, human liver cancer HepG2, and human breast cancer MCF-7 (IC_{50}values of > 50,> 50, and 22.8 µM, respectively)	
**152**	Thymidine	Others	Cyanobacterium *Moorea producens*	Not mentioned	
**153**	2,3-Dihydroxypropyl heptacosanoate	Others	Cyanobacterium *Moorea producens*	Not mentioned	
**154**	Vanillin	Others	Endophytic *Streptomyces* sp. Hedaya48 of sponge *Aplysina fistularis*	Not mentioned	[7]
**155**	5,7-Dimethoxy-4-*p*-methoxylphenylcoumarin	Others	Endophytic *Streptomyces* sp. Hedaya48 of sponge *Aplysina fistularis*	Antifungal activity against *Trichophyton rubrum*, *Trichophyton mentagrophytes*, *Microsporum gypseum*, *Epidermophyton floccosum*, *Aspergillus niger*, *Aspergillus fumigatus*, *Fusarium oxysporum*, *Candida albicans*, *Cryptococcus humicolus* at MIC = 7.5, 90, 100, 50, 20, 10, 22, 15, 10 µg/ml, respectively and MFC = 100, 90, 150, 66, 50, 35, 49, 20, 32 µg/ml, respectively	

No.	Compound	Class	Source (Marine bacteria)	Biological Activity	Ref
**156**	Sharkquinone	Quinone	*Streptomyces* sp. EGY1	Cytotoxicity against human gastric AGS cells (IC_{50}= 7.3 μM) Overcomes tumor necrosis factor-related apoptosis-inducing ligand resistance at a concentration of 10 μM in AGS cells	[8]
**157**	SS-228R	Quinone	*Streptomyces* sp. EGY1	Not mentioned	
**158**	Heliomycin	Quinone	The sponge-associated *Streptomyces* sp. SP9	HDAC inhibitory activity (IC_{50}= 29.8 ± 0.04 μg/mL)	[9]
**159**	Tetracenomycin D	Quinone	The sponge-associated *Streptomyces* sp. SP9	HDAC inhibitory activity (IC_{50}= 10.9 ± 0.02 μg/mL)	
**160**	Nocardiotide A	Peptide	The sponge-associated *Nocardiopsis* sp. UR67	Cytotoxic effects towards the murine CT26 colon carcinoma, human HeLa cervix carcinoma, and human MM.1S multiple myeloma cell lines (IC_{50} values of 12, 11, and 8 μM/mL, respectively)	[10]
**161**	Tryptophan	Nitrogenous	The sponge-associated *Nocardiopsis* sp. UR67	Not mentioned	
**162**	kynurenic acid	Nitrogenous	The sponge-associated *Nocardiopsis* sp. UR67	Not mentioned	
**163**	4-Amino-3-methoxy benzoic acid	Nitrogenous	The sponge-associated *Nocardiopsis* sp. UR67	Not mentioned	
**164**	Microluside A	Others	The broth culture of *Micrococcus* sp. EG45 cultivated from the sponge *Spheciospongia vagabunda*	Antibacterial activity against *Enterococcus faecalis* JH212 (MIC= 10 μM) and *Staphylococcus aureus* NCTC 8325 (MIC= 13 μM)	[11]
**165**	Dolastatin 12	Peptide	*Leptolyngbya cyanobacterium*	Cytotoxicity against neuro-2a cells, IC_{50} > 1 μM??	[12]
**166**	Ibu-epidemethoxylyngbyastatin 3	Peptide	*Leptolyngbya cyanobacterium*	Cytotoxicity against neuro-2a cells, IC_{50} > 10 μM??	
**167**	Apratoxin A	Peptide	Cyanobacterium *Moorea producens*	Cytotoxic compound	[13]
**168**	Apratoxin B	Peptide	Cyanobacterium *Moorea producens*	Cytotoxic compound	
**169**	Apratoxin C	Peptide	Cyanobacterium *Moorea producens*	Cytotoxic compound	
**170**	Lyngbyabellin B	Macrolide	Cyanobacterium *Moorea producens*	Not mentioned	

No.	Compound	Class	Source (Marine bacteria)	Biological Activity	Ref
**171**	Hectochlorin	Macrolide	Cyanobacterium *Moorea producens*	Not mentioned	
**172**	Fridamycin I	Others	The elicited sponge-derived bacterium *Actinokineospora spheciospongiae* sp. nov.	Not mentioned	[14]
**173**	Actinosporin G	Others	The elicited sponge-derived bacterium *Actinokineospora spheciospongiae* sp. nov.	Not mentioned	
**174**	Butylcycloheptylprodigiosin	Others	The actinomycete RA2 (isolated from the sponge *Spheciospongia mastoidea*)	Gastroprotective effect against HCl/ethanol-induced gastric lesion in rats	[15]
**175**	undecylprodigiosin	Others	The actinomycete RA2 (isolated from the sponge *Spheciospongia mastoidea*)	Gastroprotective effect against HCl/ethanol-induced gastric lesion in rats	
**No.**	**Compound**	**Class**	**Source (Marine fungi)**	**Biological Activity**	**Ref**
**176**	Pandangolide 1a	Polyketide	The fungus *Cladosporium* sp., associated with the sponge *Niphates rowi*	Not mentioned	[16]
**177**	Pandangolide 1	Polyketide	The fungus *Cladosporium* sp., associated with the sponge *Niphates rowi*	Not mentioned	
**178**	*Iso*-cladospolide B	Polyketide	The fungus *Cladosporium* sp., associated with the sponge *Niphates rowi*	Not mentioned	
**179**	Fumitremorgin C	Alkaloid	Sediment-derived fungus MR2012 *Aspergillus fumigatus*	Not mentioned	[17]
**180**	Tryprostatin B	Alkaloid	Sediment-derived fungus MR2012 *Aspergillus fumigatus*	Not mentioned	
**181**	Compound 6	Alkaloid	Sediment-derived fungus MR2012 *Aspergillus fumigatus*	Antibacterial against *Staphylococcus aureus* and *Bacillus subtilis*, with average MIC of 12.6 µg/mL and 11.9 µg/mL, respectively.	
**182**	Compound 7	Alkaloid	Sediment-derived fungus MR2012 *Aspergillus fumigatus*	Antibacterial against *Staphylococcus aureus* and *Bacillus subtilis*, with average MIC of 15.1 µg/mL and 17.2 µg/mL, respectively.	
**183**	Compound 8	Alkaloid	Sediment-derived fungus MR2012 *Aspergillus fumigatus*	Antibacterial against *Staphylococcus aureus* and *Bacillus subtilis*, with average MIC of 10.2 µg/mL and 8.7 µg/mL, respectively.	
**184**	Compound 9	Alkaloid	Sediment-derived fungus MR2012 *Aspergillus fumigatus*	Antibacterial against *Staphylococcus aureus* and *Bacillus subtilis*, with average MIC of 18.8 µg/mL and 15.7 µg/mL, respectively.	

No.	Compound	Class	Source (Marine fungi)	Biological Activity	Ref
**185**	Compound 10	Alkaloid	Sediment-derived fungus MR2012 *Aspergillus fumigatus*	Antibacterial against *Staphylococcus aureus* and *Bacillus subtilis*, with average MIC of 16.3 µg/mL and 18.2 µg/mL, respectively.	
**186**	Compound 11	Alkaloid	Sediment-derived fungus MR2012 *Aspergillus fumigatus*	Antibacterial against *Staphylococcus aureus* and *Bacillus subtilis*, with average MIC of 17.1 µg/mL and 15.8 µg/mL, respectively.	
**187**	Compound 12	Alkaloid	Sediment-derived fungus MR2012 *Aspergillus fumigatus*	Antibacterial against *Staphylococcus aureus* and *Bacillus subtilis*, with average MIC of 15.6 µg/mL and 16.0 µg/mL, respectively.	
**188**	12-Dimethoxypinselin	Others	The fungus *Scopulariopsis* sp. obtained from the hard coral *Stylophora* sp.	Not mentioned	[18]
**189**	12-*O*-acetyl-AGI-B4	Others	The fungus *Scopulariopsis* sp. obtained from the hard coral *Stylophora* sp.	Not mentioned	
**190**	Huperxanthone C	Others	The fungus *Scopulariopsis* sp. obtained from the hard coral *Stylophora* sp.	Not mentioned	
**191**	Pinselin	Others	The fungus *Scopulariopsis* sp. obtained from the hard coral *Stylophora* sp.	Not mentioned	
**192**	Sydowinin B	Others	The fungus *Scopulariopsis* sp. obtained from the hard coral *Stylophora* sp.	Not mentioned	
**193**	13-*O*-acetylsydowinin B	Others	The fungus *Scopulariopsis* sp. obtained from the hard coral *Stylophora* sp.	Not mentioned	
**194**	2,11-Dihydroxy-1-methoxy-carbonyl-9-carboxylxanthone	Others	The fungus *Scopulariopsis* sp. obtained from the hard coral *Stylophora* sp.	Not mentioned	
**195**	Sydowinin A	Others	The fungus *Scopulariopsis* sp. obtained from the hard coral *Stylophora* sp.	Not mentioned	
**196**	8-(Methoxycarbonyl)-1-hydroxy-9-oxo-9 *H*-xanthene-3-carboxylic acid	Others	The fungus *Scopulariopsis* sp. obtained from the hard coral *Stylophora* sp.	Not mentioned	
**197**	Methyl-3,8-dihydroxy-6-methyl-9-oxo-9*H*-xanthene-1-carboxylate	Others	The fungus *Scopulariopsis* sp. obtained from the hard coral *Stylophora* sp.	Not mentioned	
**198**	Sydowic acid	Terpene	The fungus *Scopulariopsis* sp. obtained from the hard coral *Stylophora* sp.	Not mentioned	
**199**	Sydonic acid	Terpene	The fungus *Scopulariopsis* sp. obtained from the hard coral *Stylophora* sp.	Not mentioned	

No.	Compound	Class	Source (Marine fungi)	Biological Activity	Ref
**200**	11-Hydroxysydonic acid	Terpene	The fungus *Scopulariopsis* sp. obtained from the hard coral *Stylophora* sp.	Not mentioned	
**201**	11,12-Dihydroxysydonic acid	Terpene	The fungus *Scopulariopsis* sp. obtained from the hard coral *Stylophora* sp.	Not mentioned	
**202**	1-Hydroxyboivinianic acid	Terpene	The fungus *Scopulariopsis* sp. obtained from the hard coral *Stylophora* sp.	Not mentioned	
**203**	Violaceol I	Others	The fungus *Scopulariopsis* sp. obtained from the hard coral *Stylophora* sp.	Cytotoxic to the murine lymphoma cell line (L5178Y) with IC_{50} value of 9.5 µM	
**204**	Violaceol II	Others	The fungus *Scopulariopsis* sp. obtained from the hard coral *Stylophora* sp.	Cytotoxic to the murine lymphoma cell line (L5178Y) with IC_{50} value of 9.2 µM	
**205**	Diorcinol	Others	The fungus *Scopulariopsis* sp. obtained from the hard coral *Stylophora* sp.	Not mentioned	
**206**	Rikuzenol	Others	The fungus *Scopulariopsis* sp. obtained from the hard coral *Stylophora* sp.	Not mentioned	
**207**	Scopulamide	Alkaloid	The fungus *Scopulariopsis* sp. obtained from the hard coral *Stylophora* sp.	Not mentioned	
**208**	Lumichrome	Alkaloid	The fungus *Scopulariopsis* sp. obtained from the hard coral *Stylophora* sp.	Not mentioned	
**209**	WIN 64821	Alkaloid	The fungus *Scopulariopsis* sp. obtained from the hard coral *Stylophora* sp.	Not mentioned	
**210**	Scopularide B	Peptide	The fungus *Scopulariopsis* sp. obtained from the hard coral *Stylophora* sp.	Not mentioned	
**211**	Scopupyrone	Others	The fungus *Scopulariopsis* sp. obtained from the hard coral *Stylophora* sp.	Not mentioned	
**212**	Pyrenochaetic acid A	Others	The fungus *Scopulariopsis* sp. obtained from the hard coral *Stylophora* sp.	Not mentioned	
**213**	7-Hydroxy-2,5-dimethylchromon	Others	The fungus *Scopulariopsis* sp. obtained from the hard coral *Stylophora* sp.	Not mentioned	
**214**	Ergosterol	Sterol	The fungus *Scopulariopsis* sp. obtained from the hard coral *Stylophora* sp.	Not mentioned	
**215**	Deuteromycol A	Others	Fungal strain MF 003 (Deuteromycete)	Not mentioned	[19]
**216**	Deuteromycol B	Others	Fungal strain MF 003 (Deuteromycete)	Not mentioned	

No.	Compound	Class	Source (Marine fungi)	Biological Activity	Ref
**217**	Cyclo-L-Ala-L-Leu	Alkaloid	The endophytic fungus *Fusarium equiseti* (isolated from the brown alga *Padina pavonica*)	Antimicrobial activity against *B. megaterium* and *C. albicans*, inhibition zones 12 and 13 mm, respectively<br>Inhibition of Hepatitis C Virus (HCV) NS3-NS4A protease (IC_{50}= 58.33 µM)	[20]
**218**	Cyclo(L-Pro-L-Val)	Alkaloid	The endophytic fungus *Fusarium equiseti* (isolated from the brown alga *Padina pavonica*)	Antimicrobial activity against *B. megaterium*, *B. subtilis* and *C. albicans*, inhibition zones 11, 18 and 9 mm, respectively<br>Inhibition of Hepatitis C Virus (HCV) NS3-NS4A protease (IC_{50}= 23.29 µM)	
**219**	Uracil	Others	The endophytic fungus *Fusarium equiseti* (isolated from the brown alga *Padina pavonica*)	Not mentioned	
**220**	Thymine	Others	The endophytic fungus *Fusarium equiseti* (isolated from the brown alga *Padina pavonica*)	Inhibition of Hepatitis C Virus (HCV) NS3-NS4A protease (IC_{50}= 51.82 µM)	
**221**	Cyclo-(Phenylalanyl-Pro-Leu-Pro)	Alkaloid	The endophytic fungus *Fusarium equiseti* (isolated from the brown alga *Padina pavonica*)	Antimicrobial activity against *S. aureus*, *B. megaterium* and *C. albicans*, inhibition zones 9, 10 and 12 mm, respectively<br>Inhibition of Hepatitis C Virus (HCV) NS3-NS4A protease (IC_{50}= 29.45 µM)	
**222**	17-Demethyl-2,11-dideoxy-rhizoxin	Others	The endophytic fungus *Fusarium equiseti* (isolated from the brown alga *Padina pavonica*)	Antimicrobial activity against *S. aureus*, *B. Megaterium* and *C. albicans*, inhibition zones 12, 8 and 19 mm, respectively<br>Inhibition of Hepatitis C Virus (HCV) NS3-NS4A protease (IC_{50}= 34.42 µM)	
**223**	ergosterol peroxide	Sterol	The endophytic fungus *Fusarium equiseti* (isolated from the brown alga *Padina pavonica*)	Not mentioned	
**224**	Ergostra-5,7-dien-3β-ol	Sterol	The endophytic fungus *Fusarium equiseti* (isolated from the brown alga *Padina pavonica*)	Inhibition of Hepatitis C Virus (HCV) NS3-NS4A protease (IC_{50}= 77.14 µM)	
**225**	3-O-β-Glucosylsitosterol	Sterol	The endophytic fungus *Fusarium equiseti* (isolated from the brown alga *Padina pavonica*)	Antimicrobial activity against *S. aureus*, *B. megaterium*, *B. subtilis* and *C. albicans*, inhibition zones 9, 14, 11 and 10 mm, respectively<br>Inhibition of Hepatitis C Virus (HCV) NS3-NS4A protease (IC_{50}= 76.56 µM)	
**226**	Bis(2-ethylhexyl)phthalate	Others	The endophytic fungus *Fusarium equiseti* (isolated from the brown alga *Padina pavonica*)	Not mentioned	
**227**	5-Chloro-3,6-dihydroxy-2-methyl-1,4-benzoquinone	Quinone	The endophytic fungus *Fusarium equiseti* (isolated from the brown alga *Padina pavonica*)	Inhibition of Hepatitis C Virus (HCV) NS3-NS4A protease (IC_{50}= 35.15 µM)	

No.	Compound	Class	Source (Marine fungi)	Biological Activity	Ref
**228**	Griseoxanthone C	Others	The endophytic fungus *Fusarium equiseti* (isolated from the brown alga *Padina pavonica*)	Antimicrobial activity against *B. Megaterium* and *B. subtilis*, inhibition zones 10 and 13 mm, respectively<br>Inhibition of Hepatitis C Virus (HCV) NS3-NS4A protease (IC_{50}= 19.88 μM)	
**229**	Chrysophanol	Quinone	The endophytic fungus *Fusarium equiseti* (isolated from the brown alga *Padina pavonica*)	Antimicrobial activity against *S. aureus*, *B. Megaterium* and *C. albicans*, inhibition zones 15, 10 and 11 mm, respectively	
**230**	ω-Hydroxyemodin	Quinone	The endophytic fungus *Fusarium equiseti* (isolated from the brown alga *Padina pavonica*)	Antimicrobial activity against *S. aureus*, *B. megaterium*, *B. subtilis* and *C. albicans*, inhibition zones 12, 17, 9 and 18 mm, respectively<br>Inhibition of Hepatitis C Virus (HCV) NS3-NS4A protease (IC_{50}= 10.71 μM)	
**231**	Cyclo(L-Tyr-L-Pro)	Peptide	The endophytic fungus *Fusarium equiseti* (isolated from the brown alga *Padina pavonica*)	Antimicrobial activity against *S. aureus*, *B. megaterium*, *B. subtilis* and *C. albicans*, inhibition zones 11, 8 and 13 mm, respectively<br>Inhibition of Hepatitis C Virus (HCV) NS3-NS4A protease (IC_{50}= 18.20 μM)	
**232**	Perlolyrine	Alkaloid	The endophytic fungus *Fusarium equiseti* (isolated from the brown alga *Padina pavonica*)	Antimicrobial activity against *S. aureus*, *B. megaterium*, *B. subtilis* and *C. albicans*, inhibition zones 14, 19, 8 and 10 mm, respectively<br>Inhibition of Hepatitis C Virus (HCV) NS3-NS4A protease (IC_{50}= 37.89 μM)	
**233**	Cordycepin	Others	The endophytic fungus *Fusarium equiseti* (isolated from the brown alga *Padina pavonica*)	Antimicrobial activity against *S. aureus*, *B. megaterium*, *B. subtilis* and *C. albicans*, inhibition zones 16, 11, 9 and 14 mm, respectively<br>Inhibition of Hepatitis C Virus (HCV) NS3-NS4A protease (IC_{50}= 22.35 μM)	
**234**	Ara-A	Others	The endophytic fungus *Fusarium equiseti* (isolated from the brown alga *Padina pavonica*)	Antimicrobial activity against *S. aureus*, *B. megaterium*, *B. subtilis* and *C. albicans*, inhibition zones 10, 12 and 12 mm, respectively<br>Inhibition of Hepatitis C Virus (HCV) NS3-NS4A protease (IC_{50}= 24.53 μM)	
**235**	Ethyl-O-β-glucoside	Others	The endophytic fungus *Fusarium equiseti* (isolated from the brown alga *Padina pavonica*)	Not mentioned	
**236**	Communiol D	Others	The endophytic fungus *Fusarium equiseti* (isolated from the brown alga *Padina pavonica*)	Not mentioned	

No.	Compound	Class	Source (Marine fungi)	Biological Activity	Ref
**237**	Peniciphenalenin D	Others	The fungus *Chrysosporium lobatum* TM-237-S5 (isolated from the sponge *Acanthella cavernosa*)	Not mentioned	[21]
**238**	Isoconiolactone	Others	The fungus *Chrysosporium lobatum* TM-237-S5 (isolated from the sponge *Acanthella cavernosa*)	Not mentioned	
**239**	Coniolactone	Others	The fungus *Chrysosporium lobatum* TM-237-S5 (isolated from the sponge *Acanthella cavernosa*)	Not mentioned	
**240**	(-)-Peniciphenalenin F	Others	The fungus *Chrysosporium lobatum* TM-237-S5 (isolated from the sponge *Acanthella cavernosa*)	Not mentioned	
**241**	(+)-8-Hydroxyscleroderolide	Others	The fungus *Chrysosporium lobatum* TM-237-S5 (isolated from the sponge *Acanthella cavernosa*)	Not mentioned	
**242**	(-)-7,8-Dihydro-3,6-dihydroxy-1,7,7,8-tetramethyl-5H-furo-[2',3':5,6]naphtho[1,8-bc]furan-5-one	Others	The fungus *Chrysosporium lobatum* TM-237-S5 (isolated from the sponge *Acanthella cavernosa*)	Not mentioned	
**243**	(+)-Scleroderolide	Others	The fungus *Chrysosporium lobatum* TM-237-S5 (isolated from the sponge *Acanthella cavernosa*)	Not mentioned	
**244**	(+)-8-Hydroxysclerodin	Others	The fungus *Chrysosporium lobatum* TM-237-S5 (isolated from the sponge *Acanthella cavernosa*)	Not mentioned	
**245**	Coniosclerodin	Others	The fungus *Chrysosporium lobatum* TM-237-S5 (isolated from the sponge *Acanthella cavernosa*)	Not mentioned	
**246**	(+)-Sclerodin	Others	The fungus *Chrysosporium lobatum* TM-237-S5 (isolated from the sponge *Acanthella cavernosa*)	Not mentioned	
**247**	Pseurotin A	Others	The fungus *Aspergillus fumigatus*	Antiseizure activity, Maximum Tolerated Concentrations (MTCs) in 7-dpf Zebrafish Larvae = 100 µg/mL	[22]
**248**	Pseurotin A_2	Others	The fungus *Aspergillus fumigatus*	Antiseizure activity, Maximum Tolerated Concentrations (MTCs) in 7-dpf Zebrafish Larvae = 12.5 µg/mL	
**249**	Pseurotin F1	Others	The fungus *Aspergillus fumigatus*	Antiseizure activity, Maximum Tolerated Concentrations (MTCs) in 7-dpf Zebrafish Larvae = 50 µg/mL	
**250**	11-*O*-methylpseurotin A	Others	The fungus *Aspergillus fumigatus*	Antiseizure activity, Maximum Tolerated Concentrations (MTCs) in 7-dpf Zebrafish Larvae = 100 µg/mL	
**251**	Pseurotin D	Others	The fungus *Aspergillus fumigatus*	Antiseizure activity, Maximum Tolerated Concentrations (MTCs) in 7-dpf Zebrafish Larvae = 100 µg/mL	

No.	Compound	Class	Source (Marine fungi)	Biological Activity	Ref
**252**	Azaspirofuran A	Others	The fungus *Aspergillus fumigatus*	Antiseizure activity, Maximum Tolerated Concentrations (MTCs) in 7-dpf Zebrafish Larvae = 12.5 µg/mL	
**253**	Azaspirofuran B	Others	The fungus *Aspergillus fumigatus*	Antiseizure activity, Maximum Tolerated Concentrations (MTCs) in 7-dpf Zebrafish Larvae = 12.5 µg/mL	
**254**	Haenamindole	Alkaloid	The Red Sea endophytic fungus *Penicillium chrysogenum*	HCV protease activity with an IC_{50} value of 76.3µM. The cytotoxicity profiling in a panel of up to 12 cell lines indicated significant cytotoxicity with pronounced selectivity for colon-38 cancer cells compared to the human normal cells.	[23]
**No.**	**Compound**	**Class**	**Source (Marine sponges)**	**Biological Activity**	**Ref**
**255**	Peyssonol A	Terpene	The sponge *Hyatella intestinalis*	Not mentioned	[24]
**256**	Peyssonol B	Terpene	The sponge *Hyatella intestinalis*	Not mentioned	
**257**	Hyatellaquinone	Terpene	The sponge *Hyatella intestinalis*	Not mentioned	
**258**	12-*O*-acetyl-16-*O*-methylhyrtiolide	Terpene	The Sponge *Hyrtios erectus*	Anti-*H. pylori* (MIC= 263.71 µM)<br>Antitubercular (MIC= 16.47 µM)<br>Cytotoxicity against MCF-7 (IC_{50}= 55.6 µM), HCT-116 (IC_{50}= 17.8 µM) and HepG2 (IC_{50}= 20.1 µM)	[25]
**259**	12-*O*-deacetyl-12,19-di-*epi*-scalarin	Terpene	The Sponge *Hyrtios erectus*	Anti-*H. pylori* (MIC= 81.38 µM)<br>Antitubercular (MIC= 20.33 µM)	
**260**	24-Methoxypetrosaspongia C	Terpene	The Sponge *Hyrtios erectus*	Anti-*H. pylori* (MIC= 16.03 µM)<br>Antitubercular (MIC= 8.02 µM)<br>Cytotoxicity against MCF-7 (IC_{50}= 54.2 µM), HCT-116 (IC_{50}= 26.5 µM) and HepG2 (IC_{50}= 26.6 µM)	[25]
				Cytotoxicity of against breast cancer MCF-7, hepatocellular carcinoma HepG2, colorectal cancer HCT-116 cell lines with IC_{50}=55.4, 25.4, 26.5 µM, respectively	[26]

No.	Compound	Class	Source (Marine sponges)	Biological Activity	Ref
**261**	12β-acetoxy,16β-methoxy,20α-hydroxy-17-scalaren-19,20-olide	Terpene	The Sponge *Hyrtios erectus*	Anti-*H. pylori* (MIC= 32.97 µM) Antitubercular (MIC= 8.24 µM) Cytotoxicity against MCF-7 (IC_{50}= 37.3 µM), HCT-116 (IC_{50}= 22.8 µM) and HepG2 (IC_{50}= 34.9 µM)	[25]
				Antiproliferative activity against MCF-7 cell line (IC_{50}= 40.3 µM), HCT-116 cell line (IC_{50}= 22.5 µM) and HepG2 cell line (IC_{50}= 42.5 µM).	[27]
**262**	12-Deacetyl-12-epi-scalaradial	Terpene	The Sponge *Hyrtios erectus*	Anti-*H. pylori* (MIC= 80.95 µM) Antitubercular (MIC= 10.12 µM) Cytotoxicity against MCF-7 (IC_{50}= 32.7 µM), HCT-116 (IC_{50}= 34.5 µM) and HepG2 (IC_{50}= 23.5 µM)	[25]
				Cytotoxicity of against breast cancer MCF-7, hepatocellular carcinoma HepG2, colorectal cancer HCT-116 cell lines with IC_{50}= 36, 23.4, 27.1 µM, respectively	[26]
**263**	12-acetoxy,16-*epi*-hyrtiolide	Terpene	The Sponge *Hyrtios erectus*	Anti-*H. pylori* (MIC= 33.97 µM) Antitubercular (MIC= 16.97 µM) Cytotoxicity against MCF-7 (IC_{50}= 34.9 µM), HCT-116 (IC_{50}= 48.6 µM) and HepG2 (IC_{50}= 27.3 µM)	[25]
				Antiproliferative activity against MCF-7 cell line (IC_{50}= 32.6 µM), HCT-116 cell line (IC_{50}= 57.5 µM) and HepG2 cell line (IC_{50}= 21.8 µM).	[27]
**264**	Chitin	Others	Non-verongiid demosponges *Acarnus wolffgangi*, *Echinoclathria gibbosa* and *Mycale euplectellioides*	Not mentioned	[28,29]
			The demosponge *Pseudoceratina arabica*	Not mentioned	[30]
**265**	Ceratinine F	Alkaloid	The Verongid sponge *Pseudoceratina arabica*	Antimigratory activity against the highly metastatic human breast cancer cell line MDA-MB-231 (Migration= 66.3%, at 10 µM)	[31]
**266**	Ceratinine G	Alkaloid	The Verongid sponge *Pseudoceratina arabica*	Antimigratory activity against the highly metastatic human breast cancer cell line MDA-MB-231 (Migration= 76.0%, at 10 µM)	

No.	Compound	Class	Source (Marine sponges)	Biological Activity	Ref
**267**	Ceratinamide A	Alkaloid	The Verongid sponge *Pseudoceratina arabica*	Antimigratory activity against the highly metastatic human breast cancer cell line MDA-MB-231 (Migration= 124.5%, at 10 µM)	
**268**	19-Hydroxyceratinamide A	Alkaloid	The Verongid sponge *Pseudoceratina arabica*	Antimigratory activity against the highly metastatic human breast cancer cell line MDA-MB-231 (Migration= 64.2%, at 10 µM)	
**269**	Scalarolide acetate	Terpene	The Sponge *Hyrtios erectus*	Anti-*H. pylori* (MIC= 146.02 µM) Antitubercular (MIC= 9.13 µM) Cytotoxicity against MCF-7 (IC_{50}= 22 µM), HCT-116 (IC_{50}= 15.2 µM) and HepG2 (IC_{50}= 15.3 µM)	[25]
			The sponge *Hyrtios erectus*	Antiproliferative activity against MCF-7 cell line (IC_{50}= 20.9 µM), HCT-116 cell line (IC_{50}= 15.4 µM) and HepG2 cell line (IC_{50}= 15.5 µM).	[27]
**270**	Xestosterol	Sterol	The sponge *Xestospongia testudinaria*	Cytotoxic activity at 50 µg/mL against HeLa cells (35.78% inhibition), HepG-2 (46.25% inhibition) and Daoy (34.07% inhibition)	[32]
**271**	Xestosterol palmitate	Sterol	The sponge *Xestospongia testudinaria*	Cytotoxic activity at 50 µg/mL against HeLa cells (7.98% inhibition) and HepG-2 (14.72% inhibition)	
**272**	Xestosterol ester of 18’-bromooctadeca-7’E,9’E-diene-7’,15’-diynoic acid	Sterol	The sponge *Xestospongia testudinaria*	Not mentioned	
**273**	Xestosterol ester of l6’-bromo-(7’E,11’E,l5’E)-hexadeca-7’,11’,l5’-triene-5’,13’-diynoic acid	Sterol	The sponge *Xestospongia testudinaria*	Not mentioned	
**274**	(5E,11E,15E,19E)-20-Bromoeicosa-5,11,15,19-tetraene-9,17-diynoic acid	Fatty acid	The sponge *Xestospongia testudinaria*	Cytotoxic activity at 50 µg/mL against HeLa cells (4.17% inhibition) and HepG-2 (2.09% inhibition)	
**275**	18,18-Dibromo- (9E)-octadeca-9,17-diene-5,7-diynoic acid	Fatty acid	The sponge *Xestospongia testudinaria*	Cytotoxic activity at 50 µg/mL against HeLa cells (87.98% inhibition), HepG-2 (89.33% inhibition) and Daoy (87.02% inhibition)	
**276**	18-Bromooctadeca-(9E,17E)-diene-7,15-diynoic acid	Fatty acid	The sponge *Xestospongia testudinaria*	Cytotoxic activity at 50 µg/mL against HeLa cells (67% inhibition), HepG-2 (18.4% inhibition) and Daoy (77.56% inhibition)	

No.	Compound	Class	Source (Marine sponges)	Biological Activity	Ref
**277**	18-Bromooctadeca-(9E,13E,17E)-triene-7,15-diynoic acid	Fatty acid	The sponge *Xestospongia testudinaria*	Not mentioned	
**278**	l6-Bromo (7E,11E,l5E)hexadeca-7,11,l5-triene-5,13-diynoic acid	Fatty acid	The sponge *Xestospongia testudinaria*	Cytotoxic activity at 50 µg/mL against HeLa cells (58.61% inhibition), HepG-2 (45.23% inhibition) and Daoy (71.58% inhibition)	
**279**	2-Methylmaleimide-5-oxime	Fatty acid	The sponge *Xestospongia testudinaria*	Not mentioned	
**280**	Maleimide-5-oxime	Fatty acid	The sponge *Xestospongia testudinaria*	Not mentioned	
**281**	Tetillapyrone	Fatty acid	The sponge *Xestospongia testudinaria*	Not mentioned	
**282**	Nortetillapyrone	Fatty acid	The sponge *Xestospongia testudinaria*	Not mentioned	
**283**	Debromohymenialdisine	Alkaloid	The sponge *Stylissa carteri*	Antiviral activity, 30%–40% inhibition of HIV-1 at 3.1 µM	[33]
**284**	Hymenialdisine	Alkaloid	The sponge *Stylissa carteri*	Antiviral activity, 30%–40% inhibition of HIV-1 at 13 µM	
**285**	Oroidin	Alkaloid	The sponge *Stylissa carteri*	Antiviral activity, 50% inhibition of HIV-1 at 50 µM<br>Inhibited the activity of the HIV-1 Reverse Transcriptase up to 90% at 25 µM	
**286**	5-(4-hydroxybenzylidene)-imidazolidine-2,4-dione	Alkaloid	The sponge *Hemimycale arabica*	Antiproliferative activity against HeLa cell line (IC_{50}= 28.3 µg/mL)<br>Antimicrobial activity against *E. coli* (inhibition Zone 18 mm, at 100 µg/disc)<br>Antifungal activity against *C. albicans* (inhibition Zone 22 mm, at 100 µg/disc)	[34]
**287**	Hemimycalin A	Alkaloid	The sponge *Hemimycale arabica*	Antimicrobial activity against *E. coli* (inhibition Zone 10 mm, at 100 µg/disc)<br>Antifungal activity against *C. albicans* (inhibition Zone 14 mm, at 100 µg/disc)	
**288**	Hemimycalin B	Alkaloid	The sponge *Hemimycale arabica*	Antimicrobial activity against *E. coli* (inhibition Zone 20 mm, at 100 µg/disc)<br>Antifungal activity against *C. albicans* (inhibition Zone 20 mm, at 100 µg/disc)	

No.	Compound	Class	Source (Marine sponges)	Biological Activity	Ref
**289**	Subereamolline C	Alkaloid	The verongid sponge *Suberea* Species	Not mentioned	[35]
**290**	Subereamolline D	Alkaloid	The verongid sponge *Suberea* Species	Not mentioned	
**291**	Aeroplysinin 1	Others	The verongid sponge *Suberea* Species	Not mentioned	
**292**	Homoaerothionin	Alkaloid	The verongid sponge *Suberea* Species	Not mentioned	[35]
			The sponge *Suberea mollis*	Antimigratory activity against the highly metastatic MDA-MB-231 human breast cancer cell line at 10 and 30 μM	[36]
			The sponge *Suberea mollis*	Antimicrobial activity against *Staphylococcus aureus* with inhibition zone of 3 mm.	[37]
**293**	Aerothionin	Alkaloid	The verongid sponge *Suberea* Species	Antiproliferative activity against HeLa cells (IC_{50}= 29 μM)	[35]
			The sponge *Suberea mollis*	Antimigratory activity against the highly metastatic MDA-MB-231 human breast cancer cell line at 10 and 30 μM	[36]
			The sponge *Suberea mollis*	Antimicrobial activity against Staphylococcus aureus and Klebsiella pneumonia with inhibition zones of 5 mm and 3 mm.	[37,38]
**294**	Hyrtioerectine D	Alkaloid	The sponge, *Hyrtios* species	Inhibition zones; 17 mm against *Candida albicans* (10 mg/mL), 20 mm against *Staphylococcus aureus* (10 μg/disc)<br>Free radical scavenging activity assay using DPPH (45% inhibition)<br>Cancer cell line inhibition; MDA-MB-231 (GI_{50}= 25 μM), A549 (GI_{50}= 30 μM), HT-29 (GI_{50}= 28 μM)	[39]
**295**	Hyrtioerectine E	Alkaloid	The sponge, *Hyrtios* species	Inhibition zones; 9 mm against *Candida albicans* (10 mg/mL), 10 mm against *Staphylococcus aureus* (10 μg/disc)<br>Free radical scavenging activity assay using DPPH (31% inhibition)<br>Cancer cell line inhibition; MDA-MB-231 (GI_{50}= 90 μM), A549 (GI_{50}= 100 μM), HT-29 (GI_{50}= 85 μM)	

No.	Compound	Class	Source (Marine sponges)	Biological Activity	Ref
**296**	Hyrtioerectine F	Alkaloid	The sponge, *Hyrtios* species	Inhibition zones; 14 mm against *Candida albicans* (10 mg/mL), 16 mm against *Staphylococcus aureus* (10 µg/disc)<br>Free radical scavenging activity assay using DPPH (42% inhibition)<br>Cancer cell line inhibition; MDA-MB-231 (GI_{50}= 42 µM), A549 (GI_{50}= 35 µM), HT-29 (GI_{50}= 45 µM)	
**297**	Ceratinine A	Alkaloid	The sponge *Pseudoceratina arabica*	Antimigratory activity against the highly metastatic MDA-MB-231 human breast cancer cell line at 10 and 30 µM	[36]
**298**	Ceratinine B	Alkaloid	The sponge *Pseudoceratina arabica*	Antimigratory activity against the highly metastatic MDA-MB-231 human breast cancer cell line at 10 and 30 µM	
**299**	Ceratinine C	Alkaloid	The sponge *Pseudoceratina arabica*	Not mentioned	
**300**	Ceratinine D	Alkaloid	The sponge *Pseudoceratina arabica*	Antimigratory activity against the highly metastatic MDA-MB-231 human breast cancer cell line at 10 and 30 µM	
**301**	Ceratinine E	Alkaloid	The sponge *Pseudoceratina arabica*	Not mentioned	
**302**	Hydroxymoloka'iamine	Alkaloid	The sponge *Pseudoceratina arabica*	Antimigratory activity against the highly metastatic MDA-MB-231 human breast cancer cell line at 30 µM	[36]
				Not mentioned	[40]
**303**	Dysidamide B	Nitrogenous	The sponge *Lamellodysidea herbacea*	Not mentioned	[41]
			The sponge *Dysidea herbacea*	Not mentioned	[42]
**304**	Dysidamide C	Nitrogenous	The sponge *Lamellodysidea herbacea*	Not mentioned	[41]
			The sponge *Dysidea herbacea*	Not mentioned	[42]
**305**	Dysidamide D	Nitrogenous	The sponge *Lamellodysidea herbacea*	Not mentioned	[41]

No.	Compound	Class	Source (Marine sponges)	Biological Activity	Ref
**306**	Dysidamide E	Nitrogenous	The sponge *Lamellodysidea herbacea*	Not mentioned	
**307**	Dysidamide F	Nitrogenous	The sponge *Lamellodysidea herbacea*	Not mentioned	
**308**	Dysidamide G	Nitrogenous	The sponge *Lamellodysidea herbacea*	Not mentioned	
**309**	Dysidamide H	Nitrogenous	The sponge *Lamellodysidea herbacea*	Not mentioned	
**310**	Unnamed	Others	The sponge *Lamellodysidea herbacea*	Not mentioned	
**311**	Unnamed	Others	The sponge *Lamellodysidea herbacea*	Not mentioned	
**312**	Naamidine A	Alkaloid	The sponge *Leucetta* cf *chagosensis*	Antifungal activity against *C. neoformans* (MIC= 12.5 μg/mL)	**[43]**
**313**	Naamidine B	Alkaloid	The sponge *Leucetta* cf *chagosensis*	Antifungal activity against *C. neoformans* (MIC= 6.25 μg/mL)	
**314**	Naamidine D	Alkaloid	The sponge *Leucetta* cf *chagosensis*	Not mentioned	
**315**	Naamidine G	Alkaloid	The sponge *Leucetta* cf *chagosensis*	Antifungal activity against *C. neoformans* (MIC= 12.5 μg/mL)	
**316**	Naamine D	Alkaloid	The sponge *Leucetta* cf *chagosensis*	Antifungal activity against *C. neoformans* (MIC= 6.25 μg/mL)	
**317**	Dihydroyardenone	Others	The sponge *Ptilocaulis spiculifer*	Not mentioned	**[44]**
**318**	Abudinol B	Others	The sponge *Ptilocaulis spiculifer*	Not mentioned	
**319**	Muzitone	Others	The sponge *Ptilocaulis spiculifer*	Not mentioned	
**320**	Nakorone	Others	The sponge *Ptilocaulis spiculifer*	Not mentioned	
**321**	Durgamone	Others	The sponge *Ptilocaulis spiculifer*	Not mentioned	
**322**	Sodwanone N	Others	The sponge *Axinella weltneri*	Not mentioned	
**323**	Sodwanone O	Others	The sponge *Axinella weltneri*	Not mentioned	
**324**	Sodwanone P	Others	The sponge *Axinella weltneri*	Not mentioned	

No.	Compound	Class	Source (Marine sponges)	Biological Activity	Ref
**325**	Sodwanone Q	Others	The sponge *Axinella weltneri*	Not mentioned	
**326**	Sodwanone R	Others	The sponge *Axinella weltneri*	Not mentioned	
**327**	Hanishenol A	Others	The Axinellid Sponge *Acanthella carteri*	Not mentioned	[45]
**328**	Hanishenol B	Others	The Axinellid Sponge *Acanthella carteri*	Not mentioned	
**329**	Erylusamine TA	Others	A Sponge identified as *Erylus cf. lendenfeidi*	Not mentioned	[46]
**330**	Erylusine	Others	A Sponge identified as *Erylus cf. lendenfeidi*	Not mentioned	
**331**	Erylusidine	Others	A Sponge identified as *Erylus cf. lendenfeidi*	Not mentioned	
**332**	Petrosynol	Others	The sponge *Petrosia* sp.	Inhibition of HIV-1 reverse transcriptase associated RDDP (IC_{50}= 15.8 µM) and DDDP (IC_{50}= 36 µM) functions	[47]
**333**	Toxicol C	Quinone	The sponge *Toxiclona toxius*	Not mentioned	[48]
**334**	Smenotronic acid	Terpene	The sponge *Smenospongia* sp.	Not mentioned	[49]
**335**	Yardenone	Terpene	The sponge *Ptilocaulis spiculifer*	Not mentioned	[50]
**336**	Abudinol	Terpene	The sponge *Ptilocaulis spiculifer*	Not mentioned	
**337**	Aaptosine	Alkaloid	The sponge *Aaptos aaptos*	Not mentioned	[51]
**338**	Salmahyrtisol A 3-acetyl	Terpene	The sponge *Hyrtios erecta*	Cytotoxicity of $IC_{50} \geq 1$ µg/mL against the three types of cells [murine leukemia (P-388), human lung carcinoma (A-549;), and human colon carcinoma (HT-29)]	[52]
**339**	Hyrtiosal	Terpene	The sponge *Hyrtios erecta*	Not mentioned	
**340**	Sesterstatin 1	Terpene	The sponge *Hyrtios erecta*	Cytotoxicity of $IC_{50} \geq 1$ µg/mL against the three types of cells [murine leukemia (P-388), human lung carcinoma (A-549;), and human colon carcinoma (HT-29)]	

No.	Compound	Class	Source (Marine sponges)	Biological Activity	Ref
**341**	Salmahyrtisol B	Terpene	The sponge *Hyrtios erecta*	Cytotoxicity of $IC_{50} \geq 1$ µg/mL against the three types of cells [murine leukemia (P-388), human lung carcinoma (A-549;), and human colon carcinoma (HT-29)]	
**342**	Salmahyrtisol C	Terpene	The sponge *Hyrtios erecta*	Not mentioned	
**343**	Scalarolide	Terpene	The sponge *Hyrtios erectus*	Not mentioned	[27]
			The sponge *Hyrtios erecta*	Not mentioned	[52]
**344**	1,4-Dideoxyhexose	Others	The sponge *Dysidea herbacea*	Not mentioned	[42]
**345**	α-D-xylopyranose	Others	The sponge *Dysidea herbacea*	Not mentioned	
**346**	Furodysinin lactone	Others	The sponge *Dysidea herbacea*	Not mentioned	
**347**	24-Methylene-5α-cholest-7-ene-3β,5,6-β-triol	Sterol	The sponge *Dysidea herbacea*	Not mentioned	
**348**	Unnamed	Sterol	The sponge *Dysidea herbacea*	Not mentioned	
**349**	Unnamed	Sterol	The sponge *Dysidea herbacea*	Not mentioned	
**350**	Unnamed	Sterol	The sponge *Dysidea herbacea*	Not mentioned	
**351**	Aikupikoxide A	Terpene	The sponge *Diacarnus erythraenus*	Cytotoxicity against: murine leukemia (P-388), human lung carcinoma (A-549) and human colon carcinoma (HT-29). IC_{50} >1 µg/mL	[53]
**352**	Aikupikoxide B	Terpene	The sponge *Diacarnus erythraenus*	Cytotoxicity against: murine leukemia (P-388), human lung carcinoma (A-549) and human colon carcinoma (HT-29). IC_{50} >1 µg/mL	
**353**	Aikupikoxide C	Terpene	The sponge *Diacarnus erythraenus*	Cytotoxicity against: murine leukemia (P-388), human lung carcinoma (A-549) and human colon carcinoma (HT-29). IC_{50} >1 µg/mL	
**354**	Aikupikoxide D	Terpene	The sponge *Diacarnus erythraenus*	Cytotoxicity against: murine leukemia (P-388), human lung carcinoma (A-549) and human colon carcinoma (HT-29). IC_{50} >1 µg/mL	
**355**	*O*-Methyl guaianediol	Terpene	The sponge *Diacarnus erythraenus*	Not mentioned	

No.	Compound	Class	Source (Marine sponges)	Biological Activity	Ref
**356**	Nuapapuin A methyl ester	Terpene	The sponge *Diacarnus erythraenus*	Not mentioned	[53]
			The sponge *Diacarnus erythraeanus*	Not mentioned	[54]
**357**	Tasnemoxide A	Terpene	The sponge *Diacarnus erythraenus*	Moderate cytotoxicity to murine leukemia (P-388;), human lung carcinoma (A-549) and human colon carcinoma (HT-29); IC_{50} >1 μg/mL	[55]
**358**	Tasnemoxide B	Terpene	The sponge *Diacarnus erythraenus*	Moderate cytotoxicity to murine leukemia (P-388;), human lung carcinoma (A-549) and human colon carcinoma (HT-29); IC_{50} >1 μg/mL	
**359**	Tasnemoxide C	Terpene	The sponge *Diacarnus erythraenus*	Moderate cytotoxicity to murine leukemia (P-388;), human lung carcinoma (A-549) and human colon carcinoma (HT-29); IC_{50} >1 μg/mL	
**360**	Dragmacidoside	Others	The ponge *Dragmacidon coccinea*	Not mentioned	[56]
**361**	Adenosine	Others	The ponge *Dragmacidon coccinea*	Not mentioned	
**362**	Inosine	Others	The ponge *Dragmacidon coccinea*	Not mentioned	
**363**	Deoxycytidine	Others	The ponge *Dragmacidon coccinea*	Not mentioned	
**364**	Methyl-a-D-glucopyranoside	Others	The ponge *Dragmacidon coccinea*	Not mentioned	
**365**	Clionasterol	Sterol	The ponge *Dragmacidon coccinea*	Not mentioned	
**366**	Stigmastero	Sterol	The ponge *Dragmacidon coccinea*	Not mentioned	
**367**	Campesterol	Sterol	The ponge *Dragmacidon coccinea*	Not mentioned	
**368**	Brassicasterol	Sterol	The ponge *Dragmacidon coccinea*	Not mentioned	
**369**	Callyptide A	Peptide	The sponge *Callyspongia* species	Not mentioned	[57]
**370**	bis-[2-ethyl]-hexyl-phthylester	Others	The sponge *Niphates*	Not mentioned	[58]
**371**	Triglyceride fatty acid ester	Fatty acid	The sponge *Niphates*	Not mentioned	

No.	Compound	Class	Source (Marine sponges)	Biological Activity	Ref
**372**	Di-isobutyl phthalate	Others	The sponge *Smenospongia*	Not mentioned	
**373**	Di-n-butyl phthalate	Others	The sponge *Smenospongia*	Not mentioned	
**374**	Linoleic acid	Fatty acid	The sponge *Smenospongia*	Not mentioned	
**375**	Hexacosa-(6Z,10Z)-dienoic acid methyl ester	Fatty acid	The sponge *Mycale euplectellioides*	Cytotoxicity of against non-small cell lung cancer A549, glioblastoma U373, prostate cancer PC-3 cell lines with IC_{50}= >100 µM	[59]
**376**	hexacosa-(6Z,10Z)-dienoic acid	Fatty acid	The sponge *Mycale euplectellioides*	Cytotoxicity of against non-small cell lung cancer A549, glioblastoma U373, prostate cancer PC-3 cell lines with IC_{50}= > 100 µM	
**377**	(icosa-(8Z,11Z)-dienoic acid methyl ester	Fatty acid	The sponge *Mycale euplectellioides*	Not mentioned	
**378**	Callimplexen A	Others	The sponge *Callyspongia aff. implexa*	Not mentioned	[60]
**379**	Gelliusterol A	Sterol	The sponge *Callyspongia aff. implexa*	Not mentioned	
**380**	β-sitosterol	Sterol	The sponges *Smenospongia, Mycale euplectellioides and* sponge *Callyspongia aff. implexa*	Not mentioned	[58-60]
**381**	Hurghamide A	Nitrogenous	The sponge *Hippospongia* sp	Not mentioned	[61]
**382**	Hurghamide B	Nitrogenous	The sponge *Hippospongia* sp	Not mentioned	
**383**	Hurghamide C	Nitrogenous	The sponge *Hippospongia* sp	Not mentioned	
**384**	Hurghamide D	Nitrogenous	The sponge *Hippospongia* sp	Not mentioned	
**385**	Sesterstatin 3	Terpene	The sponge *Hyrtios erectus*	Cytotoxicity of against breast cancer MCF-7, hepatocellular carcinoma HepG2, colorectal cancer HCT-116 cell lines with IC_{50}=> 100 µM	[26]
			The Sponge *Hyrtios erectus*	Anti-*H. pylori* (MIC= 77.73 µM)<br>Antitubercular (MIC= 19.42 µM)	[25]
**386**	Neviotine C	Terpene	The sponge *Siphonochalina siphonella*	Cytotoxicity of against human prostate PC-3, human lung tumor A549, breast cancer MCF-7 cell lines with IC_{50}= 53.6, 87.2, 45.5 µM, respectively	[62]

No.	Compound	Class	Source (Marine sponges)	Biological Activity	Ref
**387**	MEC-1-4	Others	The sponge *Mycale euplectellioides*	Not mentioned	[63]
**388**	MEC-1-7	Others	The sponge *Mycale euplectellioides*	Not mentioned	
**389**	MEC-1-8	Others	The sponge *Mycale euplectellioides*	Not mentioned	
**390**	Hyrtiosenolide A	Terpene	The sponge *Hyrtios* Species	Weak antibacterial activity against *Escherichia coli*	[64]
**391**	Hyrtiosterol	Sterol	The sponge *Hyrtios* Species	Not mentioned	
**392**	(+)-Xestospongin B	Alkaloid	The sponge *Xestospongia exigua*	Not mentioned	[65]
**393**	(+)-Araguspongine A	Alkaloid	The sponge *Xestospongia exigua*	Not mentioned	
**394**	(+)-Araguspongine D	Alkaloid	The sponge *Xestospongia exigua*	Not mentioned	
**395**	(-)-Araguspongine E	Alkaloid	The sponge *Xestospongia exigua*	Not mentioned	
**396**	(+)-Araguspongine K	Alkaloid	The sponge *Xestospongia exigua*	Not mentioned	
**397**	(+)-Araguspongine L	Alkaloid	The sponge *Xestospongia exigua*	Not mentioned	
**398**	Hyrtioerectine A	Alkaloid	The sponge *Hyrtios erectus*	Cytotoxicity against HeLa cells (IC_{50}= 10 μg/mL)	[66]
**399**	16-*Epi*-scalarolbutenolide	Terpene	The sponge *Hyrtios erecta*	40% inhibition of *Mycobacterium tuberculosis* ($H_{37}Rv$) at a concentration of 6.25 μg/mL	[67]
**400**	25-Dehydroxy-12-*epi*-deacetylscalarin	Terpene	The sponge *Hyrtios erecta*	16% inhibition of *Mycobacterium tuberculosis* ($H_{37}Rv$) at a concentration of 6.25 μg/mL	
**401**	3-Acetylsesterstatin 1	Terpene	The sponge *Hyrtios erecta*	13% inhibition of *Mycobacterium tuberculosis* ($H_{37}Rv$) at a concentration of 6.25 μg/mL	
**402**	21-Acetoxydeoxyscalarin	Terpene	The sponge *Hyrtios erecta*	Not mentioned	
**403**	Hyrtiosulawesine	Alkaloid	The sponge *Hyrtios erectus*	Antiphospholipase A_2 activity with an IC_{50} value of 14 μM	[68]
**404**	Hyrtiazepine	Alkaloid	The sponge *Hyrtios erectus*	Not mentioned	
**405**	5-Hydroxy-1*H*-indole-3-carboxylic acid methyl ester	Alkaloid	The sponge *Hyrtios erectus*	Not mentioned	

No.	Compound	Class	Source (Marine sponges)	Biological Activity	Ref
**406**	5-Hydroxyindole-3-carbaldehyde	Alkaloid	The sponge *Hyrtios erectus*	Not mentioned	[69]
**407**	Hyrtiosin A	Alkaloid	The sponge *Hyrtios erectus*	Not mentioned	
**408**	Hyrtiosin B	Alkaloid	The sponge *Hyrtios erectus*	Not mentioned	
**409**	Peroxyacarnoic acid methyl ester A	Polyketide	The sponge *Acarnus* cf. *bergquistae*	Not mentioned	
**410**	Peroxyacarnoic acid methyl ester B	Polyketide	The sponge *Acarnus* cf. *bergquistae*	Not mentioned	
**411**	Sphingosines	Nitrogenous	The sponge *Grayella cyatophora*	Not mentioned	[70]
**412**	Halichondramine	Alkaloid	The sponge *Halichondria sp.*	Not mentioned	[71]
**413**	Asmarine C	Others	The sponge *Raspailia* sp.	Not mentioned	[72]
**414**	Asmarine D	Others	The sponge *Raspailia* sp.	Not mentioned	
**415**	Asmarine E	Others	The sponge *Raspailia* sp.	Not mentioned	
**416**	Asmarine F	Others	The sponge *Raspailia* sp.	Not mentioned	
**417**	Methyl 3-oxo-cholan-24-oate	Sterol	The sponge *Raspailia* sp.	Not mentioned	
**418**	Clathsterol	Sterol	The sponge *Clathria* Species	Inhibition of HIV1 reverse transcriptase at 10 µM	[73]
**419**	Haliclonyne	Others	The sponge *Haliclona* Species	Not mentioned	[74]
**420**	Ceratinamine	Nitrogenous	The sponge *Pseudoceratina arabica*	Not mentioned	[40]
**421**	5-Bromo-2,3-dihydroxy-6-methoxybenzaldehyde	Others	The sponge *Pseudoceratina arabica*	Weak effect against *Klebsiella pneumoniae* (inhibition zone of 3 mm)	
**422**	Ceratinophenol A	Others	The Verongid sponge *Pseudoceratina arabica*	Not mentioned	[31]
			The sponge *Pseudoceratina arabica*	Moderate activity against *S. aureus* and *Pseudomonas aeruginosa* (inhibition zone of 7 and 4 mm, respectively), weak effect against *Klebsiella pneumoniae* (inhibition zone of 3 mm) and weak antifungal effect.	[40]

No.	Compound	Class	Source (Marine sponges)	Biological Activity	Ref
**423**	Siphonellinol C	Terpene	The sponge *Callyspongia* (*Siphonochalina*) *siphonella*	Weak ability to reverse P-Glycoprotein-mediated MDR to colchicines	[75]
**424**	Sipholenol I	Terpene	The sponge *Callyspongia* (*Siphonochalina*) *siphonella*	Not mentioned	
**425**	Sipholenol J	Terpene	The sponge *Callyspongia* (*Siphonochalina*) *siphonella*	Activity to reverse P-Glycoprotein-mediated MDR to colchicines	[76]
**426**	Sipholenol K	Terpene	The sponge *Callyspongia* (*Siphonochalina*) *siphonella*	Activity to reverse P-Glycoprotein-mediated MDR to colchicines	
**427**	Sipholenol M	Terpene	The sponge *Callyspongia* (*Siphonochalina*) *siphonella*	Activity to reverse P-Glycoprotein-mediated MDR to colchicines	
**428**	Siphonellinol E	Terpene	The sponge *Callyspongia* (*Siphonochalina*) *siphonella*	Activity to reverse P-Glycoprotein-mediated MDR to colchicines	
**429**	Shaagrockol B	Quinone	The sponge *Toxiclona toxius*	Antifungal activity with IC_{50} value of 6 µg/ml	[77]
			The sponge *Toxiclona toxius*	Moderate inhibitor of both DNA polymerizing functions of HIV-1 RT but failed to inhibit the RT-associated ribonuclease H activity<br>DDDP activity (IC_{50} =6.7 µM)<br>RDDP activity (IC_{50} =8.5 µM)	[78]
**430**	Latrunculeic acid	Polyketide	The sponge *Negombata magnifica*	Not mentioned	[79]
**431**	15-Methoxylatrunculin B	Polyketide	The sponge *Negombata magnifica*	Not mentioned	
**432**	Latrunculin C	Polyketide	The sponge *Negombata magnifica*	Not mentioned	
**433**	Latrunculin T	Macrolide	The sponge *Negombata magnifica*	It showed antifungal activity against *Candida albicans*	[80]
**434**	Subereamolline B	Alkaloid	The sponge *Suberea mollis*	Not mentioned	[37]
**435**	11,19-Dideoxyfistularin-3	Others	The sponge *Suberea mollis*	Not mentioned	
**436**	Aeroplysinin 2	Others	The verongid sponge *Suberea* Species	Antimigratory activity (MDA-MB-231), IC_{50}= 18 µM	[35]
			The sponge *Suberea mollis*	Antimicrobial activity against *Staphylococcus aureus* with inhibition zone of 5 mm.	[37]
**437**	Subereamine A	Alkaloid	The sponge *Suberea mollis*	Not mentioned	[38]

No.	Compound	Class	Source (Marine sponges)	Biological Activity	Ref
**438**	Subereamine B	Alkaloid	The sponge *Suberea mollis*	Not mentioned	
**439**	Subereaphenol D	Others	The sponge *Suberea mollis*	Antimicrobial activity against *Staphylococcus aureus*, *Escherichia coli* and *Candida albicans* with inhibition zones of 10 mm ,18 mm and 20 mm, respectively.<br>Antioxidant activity.<br>Cytotoxic activity against HeLa cells with IC_{50} of 19 µM.	
**440**	Dichloroverongiaquinol	Others	The sponge *Suberea mollis*	Antimicrobial activity against *Escherichia coli* with inhibition zone of 15 mm.<br>Cytotoxic activity against HeLa cells with IC_{50} of 13 µM.	
**441**	Purealdin L	Others	The sponge *Suberea mollis*	Not mentioned	
**442**	Methyl-2-epinuapapuanoate	Terpene	The sponge *Diacarnus erythraeanus*	Not mentioned	[54]
**443**	Sigmosceptrellin B methyl ester	Terpene	The sponge *Diacarnus erythraeanus*	Not mentioned	
**444**	Hurghaperoxide	Terpene	The sponge *Diacarnus erythraeanus*	Not mentioned	[54]
			Undescribed sponge	Not mentioned	[81]
**445**	(+) Dibromophakelline	Alkaloid	The sponge *Stylissa carteri* (syn. *Axinella carteri*)	Cytotoxic activity with inhibition of growth 57.0% (at concentration 10 µg/mL)	[82]
**446**	*Z*-3-Bromohymenialdisine	Alkaloid	The sponge *Stylissa carteri* (syn. *Axinella carteri*)	Inhibitor of protein kinases (VEGF-R2KT1, AURORA-A, AURORA-B, CDK4/CycD1, FAK, SRC, COT, PLK1, SAK and PDGFR-beta) at concentration of 1µg/mL<br>Cytotoxic activity with inhibition of growth 60.5% (at concentration 10 µg/mL)	
**447**	(±) Ageliferin	Alkaloid	The sponge *Stylissa carteri* (syn. *Axinella carteri*)	Not mentioned	
**448**	3,4-Dibromo-1*H* -pyrrole-2-carbamide	Alkaloid	The sponge *Stylissa carteri* (syn. *Axinella carteri*)	Inhibitor of protein kinase (VEGF-R2KT1) at concentration of 1µg/mL<br>Cytotoxic activity with inhibition of growth 38.4% (at concentration 10 µg/mL)	

No.	Compound	Class	Source (Marine sponges)	Biological Activity	Ref
**449**	(−) Clathramide C	Alkaloid	The sponge *Stylissa carteri* (syn. *Axinella carteri*)	Inhibitor of protein kinase (AURORA-A) at concentration of 1µg/mL<br>Cytotoxic activity with inhibition of growth 25.3% (at concentration 10 µg/mL)	
**450**	Agelongine	Alkaloid	The sponge *Stylissa carteri* (syn. *Axinella carteri*)	Inhibitor of protein kinase (AKT1) at concentration of 1µg/mL	
**451**	(+) Manzacidin A	Alkaloid	The sponge *Stylissa carteri* (syn. *Axinella carteri*)	Inhibitor of protein kinase (AKT1) at concentration of 1µg/mL	
**452**	(−) 3-Bromomanzacidin D	Alkaloid	The sponge *Stylissa carteri* (syn. *Axinella carteri*)	Not mentioned	
**453**	*Z*-Spongiacidin D	Alkaloid	The sponge *Stylissa carteri* (syn. *Axinella carteri*)	Inhibitor of protein kinases (VEGF-R2KT1, ARK5, AURORA-A, B-RAF-VE, CDK2/CycA, CDK4/CycD1, FAK, IGF1-R, SRC, COT, PLK-1, SAK and PDGFR-beta) at concentration of 1µg/mL<br>Cytotoxic activity with inhibition of growth 36.7% (at concentration 10 µg/mL)	
**454**	*Z*-Hymenialdisine	Alkaloid	The sponge *Stylissa carteri* (syn. *Axinella carteri*)	Inhibitor of protein kinases (VEGF-R2KT1, AKT1, ARK5, CDK2-CycA, CDK4/CycD1, FAK, COT, PLK1, SAK and PDGFR-beta) at concentration of 1µg/mL<br>Cytotoxic activity with inhibition of growth 37.0% (at concentration 10 µg/mL)	
**455**	2-Debromostevensine	Alkaloid	The sponge *Stylissa carteri* (syn. *Axinella carteri*)	Not mentioned	
**456**	2-Bromoaldisine	Alkaloid	The sponge *Stylissa carteri* (syn. *Axinella carteri*)	Not mentioned	
**457**	4-Bromo-1*H* -pyrrole-2-carbamide	Alkaloid	The sponge *Stylissa carteri* (syn. *Axinella carteri*)	Not mentioned	
**458**	*E*-Debromohymenialdisine	Alkaloid	The sponge *Stylissa carteri* (syn. *Axinella carteri*)	Inhibitor of protein kinase (VEGF-R2KT1) at concentration of 1µg/mL	
**459**	Aldisine	Alkaloid	The sponge *Stylissa carteri* (syn. *Axinella carteri*)	Not mentioned	
**460**	Sipholenol N	Terpene	The sponge *Siphonochalina siphonella*	Not mentioned	[83]
**461**	Sipholenol O	Terpene	The sponge *Siphonochalina siphonella*	Not mentioned	

No.	Compound	Class	Source (Marine sponges)	Biological Activity	Ref
**462**	Neviotine D	Terpene	The sponge *Siphonochalina siphonella*	Inhibition of RANKL induced osteoclastogenesis in RAW264 macrophages with IC_{50} value of 12.8 μM	
**463**	Neviotine A	Terpene	The sponge *Siphonochalina siphonella*	Cytotoxicity of against human prostate PC-3, human lung tumor A549, breast cancer MCF-7 cell lines with IC_{50}= 71.2, 76.3, 46.3 μM, respectively	[62]
			The sponge *Siphonochalina siphonella*	Inhibition of RANKL induced osteoclastogenesis in RAW264 macrophages with IC_{50} value of 32.8 μM	[83]
**464**	Sipholenone D	Terpene	The sponge *Siphonochalina siphonella*	Not mentioned	[84]
**465**	Sipholenol F	Terpene	The sponge *Siphonochalina siphonella*	Not mentioned	
**466**	Sipholenol H	Terpene	The sponge *Siphonochalina siphonella*	Not mentioned	
**467**	Siphonellinol B	Terpene	The sponge *Siphonochalina siphonella*	Not mentioned	
**468**	Neviotine B	Terpene	The sponge *Siphonochalina siphonella*	Not mentioned	
**469**	Dahabinone A	Terpene	The sponge *Siphonochalina siphonella*	Not mentioned	
**470**	Psammaplysin Z	Nitrogenous	The verongid sponge *Aplysinella* Species	Cytotoxic activity against MDA-MB-231, HeLa and HCT 116 cell lines (IC_{50}= 19.4, 22.2 and 8.2 μM, respectively)	[85]
**471**	19-Hydroxypsammaplysin Z	Nitrogenous	The verongid sponge *Aplysinella* Species	Cytotoxic activity against MDA-MB-231, HeLa and HCT 116 cell lines (IC_{50}= 13.2, 17.6 and 7 μM, respectively)	
**472**	Callysterol	Sterol	The sponge *Callyspongia siphonella*	Not mentioned	[86]
**473**	Cholestenone	Sterol	The sponge *Callyspongia siphonella*	Not mentioned	
**474**	5α-cholestanone	Sterol	The sponge *Callyspongia siphonella*	Not mentioned	
**475**	Stigmasterone	Sterol	The sponge *Callyspongia siphonella*	Not mentioned	
**476**	Stigmasta-4,22-dien-3,6-dione	Sterol	The sponge *Callyspongia siphonella*	Not mentioned	

No.	Compound	Class	Source (Marine sponges)	Biological Activity	Ref
**477**	Petroselenic acid	Others	The sponge *Callyspongia siphonella*	Not mentioned	
**478**	Callyspongidipeptide A	Peptide	The sponge *Callyspongia siphonella*	Not mentioned	
**479**	Callysponginol sulfate A	Others	The sponge *Callyspongia siphonella*	Not mentioned	
**480**	*N*-acetyl isatin	Nitrogenous	The sponge *Callyspongia siphonella*	Not mentioned	
**481**	Trisindoline	Nitrogenous	The sponge *Callyspongia siphonella*	Not mentioned	
**482**	1,2,3,4-tetrahydro-1- methyl-β-carboline-3-carboxylic acid	Nitrogenous	The sponge *Callyspongia siphonella*	Not mentioned	
**483**	Callystatin A	Polyketide	The sponge *Callyspongia siphonella*	Not mentioned	
**484**	Hydroxydihydrobovolide	Others	The sponge *Callyspongia siphonella*	Not mentioned	
**485**	Callyspongidic acid	Others	The sponge *Callyspongia siphonella*	Not mentioned	
**486**	Callyspongendiol	Others	The sponge *Callyspongia siphonella*	Not mentioned	
**487**	15,16-epoxy-22-hydroxysipholen-one A	Terpene	The sponge *Callyspongia siphonella*	Not mentioned	
**488**	Sipholenone C	Terpene	The sponge *Callyspongia siphonella*	Not mentioned	
**489**	Sipholenoside B	Terpene	The sponge *Siphonochalina siphonella*	Not mentioned	**[84]**
			The sponge *Callyspongia siphonella*	Not mentioned	**[86]**
**490**	Sipholenoside A	Terpene	The sponge *Siphonochalina siphonella*	Not mentioned	**[84]**
			The sponge *Callyspongia siphonella*	Not mentioned	**[86]**

No.	Compound	Class	Source (Marine sponges)	Biological Activity	Ref
**491**	Sipholenone A	Terpene	The sponge *Siphonochalina siphonella*	Cytotoxicity of against human prostate PC-3, human lung tumor A549, breast cancer MCF-7 cell lines with IC_{50}= 53.9, 24.8, 36.2 μM, respectively	[62]
			The sponge *Callyspongia* (*Siphonochalina*) *siphonella*	Weak ability to reverse P-Glycoprotein-mediated MDR to colchicines	[75]
			The sponge *Callyspongia* (*Siphonochalina*) *siphonella*	Activity to reverse P-Glycoprotein-mediated MDR to colchicines	[76]
			The sponge *Siphonochalina siphonella*	Not mentioned	[83]
			The sponge *Callyspongia siphonella*	Not mentioned	[86]
**492**	Sipholenol G	Terpene	The sponge *Callyspongia* (*Siphonochalina*) *siphonella*	Not mentioned	[76]
			The sponge *Siphonochalina siphonella*	Not mentioned	[84]
			The sponge *Callyspongia siphonella*	Not mentioned	[86]
**No.**	**Compound**	**Class**	**Source (Coral)**	**Biological Activity**	**Ref**
**493**	Hurgadacin	Sterol	The soft coral *Sinularia polydactyla*	Not mentioned	[87]
**494**	24-Methylenecholestane-3b,5a,6b-triol	Sterol	The soft coral *Sinularia polydactyla*	Not mentioned	
**495**	24-Methylenecholestane-1a,3b,5a,6b,11a-pentol	Sterol	The soft coral *Sinularia polydactyla*	Not mentioned	
**496**	Lactiflorenol	Terpene	The soft coral *Sinularia polydactyla*	Not mentioned	
**497**	Trinorcarotenolide acetate peridinin	Others	The soft coral *Sinularia polydactyla*	Not mentioned	
**498**	8,11-Epoxy-4,12-epoxy-2,6-cembradiene	Terpene	The soft coral *Sarcophyton glaucum*	Antitumor activity against mouse melanoma $B_{16}F_{10}$ cells (100% inhibition of viability at 500 μM concentration for 48 h) and cytotoxicity against monkey kidney CV-1 cells.	[88]
**499**	8,12-Epoxy-2,6-cembradiene-4,11-diol	Terpene	The soft coral *Sarcophyton glaucum*	Antitumor activity against mouse melanoma $B_{16}F_{10}$ cells (100% inhibition of viability at 500 μM concentration for 48 h), with no cytotoxicity against monkey kidney CV-1 cells.	

No.	Compound	Class	Source (Coral)	Biological Activity	Ref
**500**	Sarcophytolide	Terpene	The soft coral *Sarcophyton glaucum*	Antitumor activity against mouse melanoma $B_{16}F_{10}$ cells (100% inhibition of viability at 500 µM concentration for 48 h) and cytotoxicity against monkey kidney CV-1 cells.	
**501**	Cembra-2,7,11-trien-4,13-diol	Terpene	The soft coral *Sarcophyton glaucum*	Antitumor activity against mouse melanoma $B_{16}F_{10}$ cells (100% inhibition of viability at 500 µM concentration for 48 h) and cytotoxicity against monkey kidney CV-1 cells.	
**502**	7α,8β-dihydroxydeepoxysarcophine	Terpene	The soft coral *Sarcophyton glaucum*	Antitumor activity against mouse melanoma $B_{16}F_{10}$ cells (100% inhibition of viability at 500 µM concentration for 48 h), with no cytotoxicity against monkey kidney CV-1 cells.	**[88]**
			The soft coral *Sarcophyton auritum*	Anticancer activity against breast cell line MCF-7 (IC_{50}= 11 µg/mL) and liver HepG2 cancer cell line (IC_{50}= 18.4 µg/mL)	**[89]**
**503**	2-*Epi*-sarcophine	Terpene	The soft coral *Sarcophyton auritum*	Anticancer activity against breast cell line MCF-7 (IC_{50}= 20.6 µg/mL) and liver HepG2 cancer cell line (IC_{50}= 19.7 µg/mL)	**[89]**
**504**	2,6-Cembradiene-4,8,11,12-tetrol	Terpene	The soft coral *Sarcophyton auritum*	Anticancer activity against breast cell line MCF-7 (IC_{50}= 21.1 µg/mL) and liver HepG2 cancer cell line (IC_{50}= 20 µg/mL)	
**505**	Sinularcasbane M	Terpene	The soft coral *Sinularia polydactyla*	Not mentioned	**[90]**
**506**	Sinularcasbane N	Terpene	The soft coral *Sinularia polydactyla*	Not mentioned	
**507**	Sinularcasbane O	Terpene	The soft coral *Sinularia polydactyla*	Not mentioned	
**508**	Scabrolide F	Terpene	The soft coral *Sinularia polydactyla*	Not mentioned	
**509**	Ineleganolide	Terpene	The soft coral *Sinularia polydactyla*	Not mentioned	

No.	Compound	Class	Source (Coral)	Biological Activity	Ref
**510**	Sarcophytolide C	Terpene	The soft coral *Sarcophyton glaucum*	Anticancer activity against human hepatocellular liver carcinoma HepG2, breast MCF-7 cell line (IC_{50}= 20 and 29 µg/ml respectively)	[91]
**511**	Aromadendrene	Terpene	The soft coral *Sarcophyton glaucum*	Anticancer activity against HepG2 Human hepatocellular liver carcinoma, PC-3 Prostate cancer with (IC_{50}= 20 and 9.3 µg/ml, respectively)	
**512**	Zahramycins A	Sterol	The soft coral *Sarcophyton trocheliophorum*	Not mentioned	[92]
**513**	Zahramycins B	Sterol	The soft coral *Sarcophyton trocheliophorum*	Not mentioned	
**514**	6-Oxo-germacra-4 (15),8,11-triene	Terpene	The soft coral *Sarcophyton glaucum*	Anticancer activity against HCT116 human colon cancer HCT116 cell line with (IC_{50}= 29.4 µg/ml)	[93]
**515**	Palustrol	Terpene	The soft coral *Sarcophyton glaucum*	Not mentioned	
**516**	Sarcophinediol	Terpene	The soft coral *Sarcophyton glaucum*	Anticancer activity against human hepatocellular liver carcinoma HepG2, human colon cancer HCT116 cell line with (IC_{50}= 18.8 and 19.4 µg/ml respectively)	
**517**	Cembrene	Terpene	The soft coral *Sarcophyton glaucum*	Not mentioned	
**518**	Deoxosarcophine	Terpene	The soft coral *Sarcophyton glaucum*	Not mentioned	[91]
			The soft coral *Sarcophyton glaucum*	Anticancer activity against breast MCF-7 cell line, human colon cancer HCT116 cell line with (IC_{50}= 9.9 and 25.8 µg/ml, respectively)	[93]
**519**	Sarcophytolol	Terpene	The soft coral *Sarcophyton glaucum*	Anticancer activity against human hepatocellular liver carcinoma HepG2, Prostate cancer PC-3 with (IC_{50}= 20 and 31.5 µg/ml, respectively)	[91]
			The soft coral *Sarcophyton glaucum*	Not mentioned	[93]
**520**	Sarcophytolide B	Terpene	The soft coral *Sarcophyton glaucum*	Anticancer activity against breast cell line MCF-7 (IC_{50}= 25 µg/ml)	[91]
			The soft coral *Sarcophyton glaucum*	Anticancer activity against human hepatocellular liver carcinoma HepG2 with (IC_{50}= 19.9 µg/ml)	[93]
**521**	3-Carboxy-1-methyl pyridinium (trigonelline)	Alkaloid	The soft corals *Sarcophyton glaucum* and *Lobophyton crissum*	Not mentioned	[94]

No.	Compound	Class	Source (Coral)	Biological Activity	Ref
**522**	Trochelioid A	Terpene	The soft coral *Sarcophyton trocheliophorum*	Not mentioned	[95]
**523**	Trochelioid B	Terpene	The soft coral *Sarcophyton trocheliophorum*	Not mentioned	
**524**	One, 16-oxosarcophytonin E	Terpene	The soft coral *Sarcophyton trocheliophorum*	Not mentioned	
**525**	3-(5-hydroxy-3-hepten-6-yn-1-yl)-5-methyl-2(5H)-furanone	Terpene	The soft coral *Sarcophyton trocheliophorum*	Not mentioned	[96]
**526**	Palysterol A	Sterol	The zoanthid *Palythoa tuberculosa*	Anticancer activity against human breast cancer MCF-7 and human colon carcinoma HT-29 with (IC_{50}= 170 and 178 µM) in, respectively, and (IC_{50} > 200 µM) in cervical cancer cell HeLa and KMST-6 human fibroblast cell lines	[97]
**527**	Palysterol B	Sterol	The zoanthid *Palythoa tuberculosa*	Not mentioned	
**528**	Palysterol C	Sterol	The zoanthid *Palythoa tuberculosa*	Not mentioned	
**529**	Palysterol D	Sterol	The zoanthid *Palythoa tuberculosa*	Not mentioned	
**530**	Palysterol E	Sterol	The zoanthid *Palythoa tuberculosa*	Not mentioned	
**531**	Palysterol F	Sterol	The zoanthid *Palythoa tuberculosa*	Anticancer activity against MCF-7 breast cell line, HT-29 colon adenocarcinoma cell line, HeLa cervical cancer cell and KMST-6 human fibroblast cell lines, with IC_{50}= 82, 122, 126 and 128 µM respectively.	
**532**	Xenialactol D	Terpene	The soft corals *Xenia macrospiculata*, *Xenia obscuronata* and *Xenia lilielae*	Not mentioned	[98]
**533**	Xeniolide E	Terpene	The soft coral *Xenia obscuronata*	Not mentioned	
**534**	14(15)-Epoxyxeniaphyllene	Terpene	The soft coral *Xenia lilielae*	Not mentioned	
**535**	4, 14-Diepoxyxeniaphyllene	Terpene	The soft coral *Xenia lilielae*	Not mentioned	
**536**	4, 5-Epoxyxeniaphyllan-14, 15-diol	Terpene	The soft corals *Xenia macrospiculata* and *Xenia obscuronata*	Not mentioned	
**537**	4, 14- Diepoxy-xeniaphyllenol-A	Terpene	The soft coral *Xenia lilielae*	Not mentioned	

No.	Compound	Class	Source (Coral)	Biological Activity	Ref
**538**	Xeniaphyllenol B	Terpene	The soft coral *Xenia macrospiculata*	Not mentioned	
**539**	Xeniaphyllenol C	Terpene	The soft coral *Xenia macrospiculata*	Not mentioned	
**540**	Xeniaphyllantriol	Terpene	The soft coral *Xenia obscuronata*	Not mentioned	
**541**	Pachycladin B	Terpene	The soft coral *Cladiella pachyclados*	Moderate anti-migratory activity against human prostate cancer PC-3 cell lines at a 50 µM dose.	[99]
**542**	Pachycladin C	Terpene	The soft coral *Cladiella pachyclados*	Moderate anti-migratory activity against human prostate cancer PC-3 cell lines at a 50 µM dose.	
**543**	Pachycladin E	Terpene	The soft coral *Cladiella pachyclados*	Not mentioned	
**544**	Pachycladin A	Terpene	The soft coral *Cladiella pachyclados*	Anti-migratory and anti-invasive activities against human prostate cancer PC-3 cell lines at a 50 µM dose.	
**545**	Pachycladin D	Terpene	The soft coral *Cladiella pachyclados*	Anti-migratory activity against human prostate cancer PC-3 cell lines at a 50 µM dose.	
**546**	Sclerophytin A	Terpene	The soft coral *Cladiella pachyclados*	Anti-migratory and anti-invasive activities against human prostate cancer PC-3 cell lines at a 50 µM dose.	
**547**	Cladiellisin	Terpene	The soft coral *Cladiella pachyclados*	Not mentioned	
**548**	3-Acetylcladiellisin	Terpene	The soft coral *Cladiella pachyclados*	Anti-migratory activity against human prostate cancer PC-3 cell lines at a 50 µM dose	
**549**	3, 6-Diacetylcladiellisin	Terpene	The soft coral *Cladiella pachyclados*	Anti-migratory activity against human prostate cancer PC-3 cell lines at a 50 µM dose	
**550**	(+)-Polyanthelin A	Terpene	The soft coral *Cladiella pachyclados*	Anti-migratory and anti-invasive activities against human prostate cancer PC-3 cell lines at a 50 µM dose.	
**551**	Klysimplexin G	Terpene	The soft coral *Cladiella pachyclados*	Anti-migratory and anti-invasive activities against human prostate cancer PC-3 cell lines at a **10** µM dose.	
**552**	Klysimplexin E	Terpene	The soft coral *Cladiella pachyclados*	Not mentioned	
**553**	Sclerophytin F methyl ether	Terpene	The soft coral *Cladiella pachyclados*	Anti-migratory and anti-invasive activities against human prostate cancer PC-3 cell lines at a 50 µM dose.	

No.	Compound	Class	Source (Coral)	Biological Activity	Ref
**554**	(6*Z*)-cladiellin (cladiella-6*Z*,11(17)-dien-3-ol)	Terpene	The soft coral *Cladiella pachyclados*	Moderate anti-migratory activity against human prostate cancer PC-3 cell lines at a 50 µM dose	
**555**	Sclerophytin B	Terpene	The soft coral *Cladiella pachyclados*	Moderate anti-migratory activity against human prostate cancer PC-3 cell lines at a 50 µM dose	
**556**	Patagonicol	Terpene	The soft coral *Cladiella pachyclados*	Not mentioned	
**557**	Sarcophytol M (or serratol)	Terpene	The soft coral *Litophyton arboreum*	Moderate cytotoxic against HeLa cells (IC_{50} = 8.1 µg/mL)<br>100% inhibitory activity against HIV-1 PR at 100 µg/mL.	[100]
**558**	Alismol	Terpene	The soft coral *Litophyton arboreum*	Weak cytotoxicity (>10 µg/mL) against HeLa, Vero and U937 cells.<br>96.2% inhibitory activity against HIV-1 PR at 100 µg/mL.	
**559**	24-Methylcholesta-5,24(28)-diene-3β-ol	Sterol	The soft coral *Litophyton arboreum*	Weak cytotoxicity (>10 µg/mL) against HeLa, Vero and U937 cells.	
**560**	10-*O*-methyl alismoxide	Terpene	The soft coral *Litophyton arboreum*	Weak cytotoxicity (>10 µg/mL) against HeLa, Vero and U937 cells.	
**561**	Alismoxide	Terpene	The soft coral *Litophyton arboreum*	Weak cytotoxicity (>10 µg/mL) against HeLa, Vero and U937 cells.	
**562**	(*S*)-Chimyl alcohol	Sterol	The soft coral *Litophyton arboreum*	Weak cytotoxicity (>10 µg/mL) against HeLa, Vero and U937 cells.<br>100% inhibitory activity against HIV-1 PR at 100 µg/mL.	
**563**	24-methylcholesta-5,24 (28)-diene-3β,7β,19-triol	Sterol	The soft coral *Litophyton arboreum*	Cytotoxic with moderate selective activity in HeLa cells (IC_{50} = 3.4 µg/mL)<br>Moderate cytotoxic and selective activity in U937 cells.	
**564**	11(*S*)-Hydroperoxylsarcoph-12(20)-ene	Terpene	The soft coral *Sarcophyton glaucum*	Not mentioned	[101]
**565**	(2*S**,3*S**,4*E*,8 *E*)-2*N*-[tetradecanoyl]-4(*E*),8(*E*)-icosadiene-1,3-diol	Nitrogenous	Black coral *Antipathes dichotoma*	Antibacterial activity against *Bacillus subtilis* and *Pseudomonas aeruginosa* at concentration level of 1 mg/mL with inhibition zone 17.9 mm and 18.2 mm, respectively.	[102]
**566**	(22*E*)-methylcholesta-5,22-diene-1α,3β,7α -triol.	Sterol	Black coral *Antipathes dichotoma*	Antibacterial activity against *Bacillus subtilis* at concentration level of 1 mg/mL with inhibition zone 12.7 mm.	

No.	Compound	Class	Source (Coral)	Biological Activity	Ref
**567**	3β,7α -dihydroxy-cholest-5-ene	Sterol	Black coral *Antipathes dichotoma*	Not mentioned	
**568**	(22*E*,24*S*),5α ,8α-epidioxy-24-methylcholesta-6,22-dien-3β-ol	Sterol	Black coral *Antipathes dichotoma*	Antibacterial activity against *Bacillus subtilis* at concentration level of 1 mg/mL with inhibition zone 17.9 mm.	
**569**	(22*E*,24*S*),5α ,8 α -epidioxy-24-methylcholesta-6,9(11),22-trien-3β-ol	Sterol	Black coral *Antipathes dichotoma*	Antibacterial activity against *Bacillus subtilis* at concentration level of 1 mg/mL with inhibition zone 17.6 mm.	
**570**	Compound 1	Fatty acid	The soft coral *Sarcophyton trocheliophorum*	Toxic to *Artemisia salina* shrimp at a minimal lethal dose of 8.3 µg/mL. Antibacterial against *Staphylococcus aureus* and *Bacillus subtilis*, with inhibition zone of 11.5 mm and 13.0 mm, respectively, at 10 µg per test disk.	[103]
**571**	Compound 2	Fatty acid	The soft coral *Sarcophyton trocheliophorum*	Toxic to *Artemisia salina* shrimp at a minimal lethal dose of 61.5 µg/mL. Antibacterial against *Staphylococcus aureus* and *Bacillus subtilis*, with inhibition zone of 13.2 mm and 14.9 mm, respectively, at 10 µg per test disk.	
**572**	Compound 3	Fatty acid	The soft coral *Sarcophyton trocheliophorum*	Toxic to *Artemisia salina* shrimp at a minimal lethal dose of 0.8 µg/mL. Antibacterial against *Staphylococcus aureus* and *Bacillus subtilis*, with inhibition zone of 8.5 mm and 7.6 mm, respectively, at 10 µg per test disk.	
**573**	Compound 4	Fatty acid	The soft coral *Sarcophyton trocheliophorum*	Toxic to *Artemisia salina* shrimp at a minimal lethal dose of 3.2 µg/mL. Antibacterial against *Staphylococcus aureus* and *Bacillus subtilis*, with inhibition zone of 10.3mm and 13.9 mm, respectively, at 10 µg per test disk.	
**574**	Compound 5	Fatty acid	The soft coral *Lithophyton arboreum*	Toxic to *Artemisia salina* shrimp at a minimal lethal dose of 15.3 µg/mL. Antibacterial against *Staphylococcus aureus* and *Bacillus subtilis*, with inhibition zone of 7.8 mm and 5.6 mm, respectively, at 10 µg per test disk.	

No.	Compound	Class	Source (Coral)	Biological Activity	Ref
**575**	Compound 6	Fatty acid	The soft coral *Lithophyton arboreum*	Toxic to *Artemisia salina* shrimp at a minimal lethal dose of 21.4 µg/mL. Antibacterial against *Staphylococcus aureus* and *Bacillus subtilis*, with inhibition zone of 18.6 mm and 14.7 mm, respectively, at 10 µg per test disk.	
**576**	Eicosatetraenoic acid (Arachidonic acid)	Fatty acid	The soft corals *Sarcophyton trocheliophorum* and *Lithophyton arboreum*	Not mentioned	
**577**	Eicosapentaenoic acid	Fatty acid	The soft corals *Sarcophyton trocheliophorum* and *Lithophyton arboreum*	Not mentioned	
**578**	Docosahexaenoic acid	Fatty acid	The soft corals *Sarcophyton trocheliophorum* and *Lithophyton arboreum*	Not mentioned	
**579**	(5*Z*,13*E*,15*S*)-15-Hydroxy-9-oxo-prosta-5,8(12),13-trien-1-oic acid	Others	The soft coral *Sarcophyton trocheliophorum*	Not mentioned	
**580**	Methyl (5*Z*,13*E*,15*S*)-15-Hydroxy-9-oxo-prosta-5,8(12),13-trien-1-oate	Others	The soft coral *Lithophyton arboreum*	Not mentioned	
**581**	ent-Bicyclogermacrene	Terpene	The gray morph of the soft coral *Parerythropodium fulvum fulvum*	Not mentioned	**[104]**
**582**	(-)-l(10)-Aristolene	Terpene	The gray morph of the soft coral *Parerythropodium fulvum fulvum*	Not mentioned	
**583**	Fulfulvene	Terpene	The yellow morph of the soft coral *Parerythropodium fulvum fulvum*	Not mentioned	
**584**	3-*O*-Acetylbicyclogermacrene	Terpene	The yellow morph of the soft coral *Parerythropodium fulvum fulvum*	Not mentioned	
**585**	Lemnacarnol	Terpene	The gray and yellow morphs of the soft coral *Parerythropodium fulvum fulvum*	Not mentioned	
**586**	2-Oxolemnacamol	Terpene	The gray and yellow morphs of the soft coral *Parerythropodium fulvum fulvum*	Not mentioned	
**587**	2-*O*-Acetyllemnacarnol	Terpene	The gray morph of the soft coral *Parerythropodium fulvum fulvum*	Not mentioned	
**588**	7-epi-Lemnacarnol	Terpene	The gray morph of the soft coral *Parerythropodium fulvum fulvum*	Not mentioned	
**589**	6α-Acetyl-4β,5β-dimethyl-l(10)-α-epoxy-2α-hydroxy-7-oxodecalin	Terpene	The gray and yellow morphs of the soft coral *Parerythropodium fulvum fulvum*	Not mentioned	

No.	Compound	Class	Source (Coral)	Biological Activity	Ref
**590**	6α-Acetyl-4β,5β-dimethyl-l(10)-α-epoxy-2-*O*-acetyl-7-oxodecalin	Terpene	The yellow morph of the soft coral *Parerythropodium fulvum fulvum*	Not mentioned	
**591**	5-Hydroxy-8-methoxy-calamanene	Terpene	The gray morph of the soft coral *Parerythropodium fulvum fulvum*	Not mentioned	
**592**	5-Hydroxy-8-methoxy-calamanen-15-al	Terpene	The gray morph of the soft coral *Parerythropodium fulvum fulvum*	Not mentioned	
**593**	4-Acetoxy-5,10-dihydroxyneolemna-2,8-diene	Terpene	The gray morph of the soft coral *Parerythropodium fulvum fulvum*	Not mentioned	
**594**	4-Acetoxy-5,10-di-O-acetylneolemna-2,8-diene	Terpene	The gray morph of the soft coral *Parerythropodium fulvum fulvum*	Not mentioned	
**595**	9-Oxo-9,11-secogorgost-5-ene-3ß,11-diol	Others	The gray and yellow morphs of the soft coral *Parerythropodium fulvum fulvum*	Not mentioned	
**596**	l l-Epiacetoxysinulariolide	Terpene	The soft coral *Sinularia notanda*	Not mentioned	**[105]**
**597**	11-Dehydrosinulariolide	Terpene	The soft coral *Sinularia notanda*	Not mentioned	
**598**	5-*epi*-sinuleptolide	Terpene	The soft coral *Sinularia gardineri*	Not mentioned	**[106]**
**599**	Sinuleptolide	Terpene	The soft coral *Sinularia gardineri*	Not mentioned	
**600**	7-Acetyl-8-*epi*-sinumaximol G	Terpene	The soft coral *Sarcophyton* sp.	*In vitro* antiproliferative activity against MCF-7 cells (IC_{50}= 23.84 µg/mL)	**[107]**
**601**	8-*epi*-sinumaximol G	Terpene	The soft coral *Sarcophyton* sp.	*In vitro* antiproliferative activity against MCF-7 cells (IC_{50}= 26.22 µg/mL)	
**602**	12-Acetyl-7, 12-*epi*-sinumaximol G	Terpene	The soft coral *Sarcophyton* sp.	*In vitro* antiproliferative activity against MCF-7 cells (IC_{50}= 26.81 µg/mL)	
**603**	12-Hydroxysarcoph-10-ene	Terpene	The soft coral *Sarcophyton* sp.	*In vitro* antiproliferative activity against MCF-7 cells (IC_{50}= 25.28 µg/mL)	
**604**	8-Hydroxy-*epi*-sarcophinone	Terpene	The soft coral *Sarcophyton* sp.	*In vitro* antiproliferative activity against MCF-7 cells (IC_{50}= 27.2 µg/mL)	
**605**	Sinumaximol G	Terpene	The soft coral *Sarcophyton* sp.	*In vitro* antiproliferative activity against MCF-7 cells (IC_{50}= 24.97 µg/mL)	
**606**	12-*O*-acetyl-nardosinan-6-en-1-one	Terpene	Red Sea octocoral *Rhytisma fulvum fulvum*	Cytotoxic activity against NCI-H1299, HepG2 and MCF-7 cells	**[108]**
**607**	6β-acetyl-1(10)-α-13-nornardosin-7-one	Terpene	Red Sea octocoral *Rhytisma fulvum fulvum*	Cytotoxic activity against NCI-H1299, HepG2 and MCF-7 cells	

No.	Compound	Class	Source (Coral)	Biological Activity	Ref
**608**	6,7-seco-13-nornardosinane	Terpene	Red Sea octocoral *Rhytisma fulvum fulvum*	Cytotoxic activity against NCI-H1299, HepG2 and MCF-7 cells	
**609**	6α-acetyl-1(10)-α-13-nornardosin-7-one	Terpene	Red Sea octocoral *Rhytisma fulvum fulvum*	Cytotoxic activity against NCI-H1299, HepG2 and MCF-7 cells	
**610**	12-Acetoxy-l(10)-aristolene	Terpene	Red Sea octocoral *Rhytisma fulvum fulvum*	Cytotoxic activity against NCI-H1299, HepG2 and MCF-7 cells	
**611**	4-Acetoxy-2,8-neolemnadien-5-one	Terpene	Red Sea octocoral *Rhytisma fulvum fulvum*	Cytotoxic activity against NCI-H1299, HepG2 and MCF-7 cells<br>Antibacterial activity against *B. cereus*, *S. aureus* and *Pseudomonas sp.*<br>Antifungal activity against *Aspergillus niger* and *Fusarium oxysporum*	
**612**	Nephthenol	Terpene	Red Sea octocoral *Rhytisma fulvum fulvum*	Cytotoxic activity against NCI-H1299, HepG2 and MCF-7 cells<br>Antibacterial activity against *B. cereus*, *S. aureus* and *Pseudomonas sp.*	
**613**	24-Methylcholesterol	Sterol	Red Sea octocoral *Rhytisma fulvum fulvum*	Cytotoxic activity against NCI-H1299, HepG2 and MCF-7 cells	
**614**	23,24-methylenecholesterol	Sterol	Red Sea octocoral *Rhytisma fulvum fulvum*	Antifungal activity against *Fusarium oxysporum*	
**615**	Sarcophine	Terpene	The soft coral *Sarcophyton glaucum*	Antitumor activity against mouse melanoma $B_{16}F_{10}$ cells (100% inhibition of viability at 500 µM concentration for 48 h), with no cytotoxicity against monkey kidney CV-1 cells.<br>Moderate antifungal activity against *Cryptococcus neoformans* with an IC_{50} value of 20 µg/mL	[88]
			The soft coral *Sarcophyton auritum*	Anticancer activity against breast cell line MCF-7 (IC_{50}= 23 µg/mL) and liver HepG2 cancer cell line (IC_{50}= 22.4 µg/mL)	[89]
			The soft coral *Sarcophyton glaucum*	Not mentioned	[91]
			The soft coral *Sarcophyton glaucum*	Not mentioned	[93]
			The soft coral *Sarcophyton trocheliophorum*	Not mentioned	[95]
			The soft coral *Sarcophyton glaucum*	Not mentioned	[101]
			The soft coral *Sarcophyton* sp.	*In vitro* antiproliferative activity against MCF-7 cells (IC_{50}= 22.39 µg/mL)	[107]

No.	Compound	Class	Source (Marine hydroid)	Biological Activity	Ref
**616**	Lytophilippine A	Macrolide	The hydroid *Lytocarpus philippinus*	Antibacterial activity against *E. coli* (inhibition zone= 26.3 mm, 10 mg was applied on 50.8 mm paper disk)<br>Crown gall tumor inhibition, inoculated with *Agrobacterium tumefaciens* (Inhibition= 28%)<br>Brine shrimp lethality, against *Artemia salina* (minimum lethal dose= 3.2 µg/mL)	**[109]**
**617**	Lytophilippine B	Macrolide	The hydroid *Lytocarpus philippinus*	Antibacterial activity against *E. coli* (inhibition zone= 20.4 mm, 10 mg was applied on 50.8 mm paper disk)<br>Crown gall tumor inhibition, inoculated with *Agrobacterium tumefaciens* (Inhibition= 68%)<br>Brine shrimp lethality, against *Artemia salina* (minimum lethal dose= 6.4 µg/mL)	
**618**	Lytophilippine C	Macrolide	The hydroid *Lytocarpus philippinus*	Antibacterial activity against *E. coli* (inhibition zone= 19.5 mm, 10 mg was applied on 50.8 mm paper disk)<br>Crown gall tumor inhibition, inoculated with *Agrobacterium tumefaciens* (Inhibition= 65%)<br>Brine shrimp lethality, against *Artemia salina* (minimum lethal dose= 4.8 µg/mL)	
**No.**	**Compound**	**Class**	**Source (Marine nudibranchs)**	**Biological Activity**	**Ref**
**619**	Hurghadin	Others	The nudibranch *Hexabranchus sanguineus*	Not mentioned	**[110]**
**No.**	**Compound**	**Class**	**Source (Seaweeds)**	**Biological Activity**	**Ref**
**620**	7-Oxo-cholest-5(6)-en-3-ol	Sterol	Seaweed *Jania rubens*	Not mentioned	**[111]**
**621**	Cholesterol	Sterol	Seaweed *Jania rubens*	Not mentioned	

No.	Compound	Class	Source (Marine ascidian)	Biological Activity	Ref
**622**	Didemnaketal D	Others	Marine ascidian species belonging to the genus *Didemnum*	moderate antibacterial activity against *S. aureus* (11 mm inhibition zone, at 1000 µg/mL)	[112]
**623**	Didemnaketal E	Others	Marine ascidian species belonging to the genus *Didemnum*	moderate antibacterial activity against *B. subtilis* (11 mm inhibition zone, at 1000 µg/mL)	
**No.**	**Compound**	**Class**	**Source (Seagrass)**	**Biological Activity**	**Ref**
**624**	Rutin	Phenolic	Seagrass, *Thalassodendron ciliatum*	Anticancer activity against human colorectal carcinoma HCT-116 and human liver cancer HEPG cell lines with (IC_{50}= 20 and 32.76 µM respectively)	[113,114]
**625**	3-Hydroxyasebotin	Phenolic	Seagrass, *Thalassodendron ciliatum*	Anticancer activity against human colorectal carcinoma HCT-116 with (IC_{50}= 9.77 µM)	
**626**	Quercetin-3-O-β-D-xylopyranoside	Phenolic	Seagrass, *Thalassodendron ciliatum*	Anticancer activity against human colorectal carcinoma HCT-116 and human liver cancer HEPG cell lines with (IC_{50}= 11.17 and 7.25 µM respectively	
**627**	Catechin	Phenolic	Seagrass, *Thalassodendron ciliatum*	Anticancer activity against human colorectal carcinoma HCT-116 with (IC_{50}= 49.95 µM)	
**628**	*Trans*-Caffeic acid	Phenolic	Seagrass, *Thalassodendron ciliatum*	Anticancer activity against human colorectal carcinoma HCT-116 with (IC_{50}= 23.03 and 17.48 µM respectively)	
**629**	Quercetin 3,7-diglucoside	Phenolic	Seagrass, *Thalassodendron ciliatum*	Not mentioned	[115]
**630**	Protocatechuic acid	Others	Seagrass, *Thalassodendron ciliatum*	Not mentioned	
**631**	Ferulic acid	Others	Seagrass, *Thalassodendron ciliatum*	Not mentioned	
**632**	*p*-Hydroxybenzoic acid	Others	Seagrass, *Thalassodendron ciliatum*	Not mentioned	

No.	Compound	Class	Source (Marine algae)	Biological Activity	Ref
**633**	(12*Z*)-*cis*-maneonene D	Polyketide	The red alga *Laurencia obtusa*	Stimulates apoptosis of peripheral blood neutrophils after incubation for 24 h	[116]
**634**	(12*E*)-*cis*-maneonene E	Polyketide	The red alga *Laurencia obtusa*	Stimulates apoptosis of peripheral blood neutrophils after incubation for 24, 48 and 72 h	
**635**	(12*Z*)-*trans*-maneonene C	Polyketide	The red alga *Laurencia obtuse*	Not mentioned	
**636**	*Cis*-maneonene A	Polyketide	The red alga *Laurencia obtusa*	Not mentioned	
**637**	Colpol	Others	The alga *Colpomenia sinuosa*	*In vitro* cytotoxicity towards P388, A549, HT-29, and CV-1 tumor cells, with IC_{50} values of 10 µg/ml against the four cell lines	[117]
**638**	Saringosterone	Sterol	The brown alga *Sargassum asperifolium*	Not mentioned	[118]
**639**	Saringosterol	Sterol	The brown alga *Sargassum asperifolium*	Not mentioned	
**640**	Dictyone	Terpene	The brown alga *Sargassum asperifolium*	Not mentioned	[118]
			The brown alga, *Dictyota dichotoma*	Not mentioned	[119]
**641**	Dictyone acetate	Terpene	The brown alga *Sargassum asperifolium*	Not mentioned	[118]
			The brown alga, *Dictyota dichotoma*	Not mentioned	[119]
**642**	6β-Hydroxycholest-4-en-3-one	Sterol	The red alga *Jania adhaerens*	Not mentioned	[120]
**643**	6β-Hydroxycholest-4,22-dien-3-one	Sterol	The red alga *Jania adhaerens*	Not mentioned	
**644**	16β-Hydroxy-5α-cholestan-3,6-dione	Sterol	The red alga *Jania adhaerens*	Not mentioned	
**645**	6β,16β-Dihydroxycholest-4-en-3-one	Sterol	The red alga *Jania adhaerens*	Protective antigenotoxic activity in human peripheral blood cells	
**646**	11-Hydroxypachydictyol A (dictyol E)	Terpene	The brown alga, *Dictyota dichotoma*	Not mentioned	[119]
**647**	4-Epoxy13-hydroxy pachydictyol A	Terpene	The brown alga, *Dictyota dichotoma*	Not mentioned	

No.	Compound	Class	Source (Marine algae)	Biological Activity	Ref
**648**	Pachydictyol A	Terpene	The brown alga *Dictyota dichotoma*	Not mentioned	[119]
				Cytotoxic activity against twelve human tumor cell lines (BXF, bladder; CEXF, cervix; CX,F colorectal; GXF, gastric; LXF, lung; MAXF, breast; MEXF, melanoma xenograft; OVXF, ovarian cancer xenograft; PRXF, prostate; PXF, pleuramesotheliom; RXF, renal; and UXF, uterus body) with a mean IC_{50} value of >23.6 µM/mL.	[121]
**649**	12-Hydroxy isolaurene	Terpene	The red alga *Laurencia obtusa*	Not mentioned	[122]
**650**	8,11-Dihydro-12-hydroxy isolaurene	Terpene	The red alga *Laurencia obtusa*	Not mentioned	
**651**	Isolauraldehyde	Terpene	The red alga *Laurencia obtusa*	Antimicrobial activity against *Bacillus subtilis* and *Staphylococcus aureus* at µg/ml (MIC 35 and 27 µg/mL, respectively)<br>Antifungal activity against *Candida albicans* (MIC of 70 µg/mL	
**652**	α-Chamigrene	Terpene	The red alga *Laurencia obtusa*	Not mentioned	
**653**	Cholest-4-en-3-one	Sterol	The red alga *Laurencia obtusa*	Not mentioned	
**654**	*cis*-Pachydictyol B	Terpene	The brown alga *Dictyota dichotoma*	Antimicrobial activity against *Mucor miehei, Candida albicans* and *Pythium ultimum* with inhibition zones of 20 mm, 11 mm and 12 mm at 10 µg/paper disc<br>Cytotoxic activity against twelve human tumor cell lines (BXF, bladder; CEXF, cervix; CX,F colorectal; GXF, gastric; LXF, lung; MAXF, breast; MEXF, melanoma xenograft; OVXF, ovarian cancer xenograft; PRXF, prostate; PXF, pleuramesotheliom; RXF, renal; and UXF, uterus body) with a mean IC_{50} value of >30.0 µM/mL.	[121]
**655**	*trans*-Pachydictyol B	Terpene	The brown alga *Dictyota dichotoma*	Not mentioned	

No.	Compound	Class	Source (Marine algae)	Biological Activity	Ref
**656**	Pachydictyol C	Terpene	The brown alga *Dictyota dichotoma*	Cytotoxic activity against twelve human tumor cell lines (BXF, bladder; CEXF, cervix; CX,F colorectal; GXF, gastric; LXF, lung; MAXF, breast; MEXF, melanoma xenograft; OVXF, ovarian cancer xenograft; PRXF, prostate; PXF, pleuramesotheliom; RXF, renal; and UXF, uterus body) with a mean IC_{50} value of >30.0 µM/mL.	
**657**	Dictyol E	Terpene	The brown alga *Dictyota dichotoma*	Cytotoxic activity against twelve human tumor cell lines (BXF, bladder; CEXF, cervix; CX,F colorectal; GXF, gastric; LXF, lung; MAXF, breast; MEXF, melanoma xenograft; OVXF, ovarian cancer xenograft; PRXF, prostate; PXF, pleuramesotheliom; RXF, renal; and UXF, uterus body) with a mean IC_{50} value of >30.0 µM/mL.	
**658**	*cis*-Africanan-1α-ol	Others	The brown alga *Dictyota dichotoma*	Cytotoxic activity against twelve human tumor cell lines (BXF, bladder; CEXF, cervix; CX,F colorectal; GXF, gastric; LXF, lung; MAXF, breast; MEXF, melanoma xenograft; OVXF, 747ovarian cancer xenograft; PRXF, prostate; PXF, pleuramesotheliom; RXF, renal; and UXF, uterus body) with a mean IC_{50} value of >10.0 µM/mL.	
**659**	Tetrahydrothiophen-1,1-dioxide	Others	The brown alga *Dictyota dichotoma*	Not mentioned	
**660**	Poly-β-hydroxybutyric acid	Others	The brown alga *Dictyota dichotoma*	Not mentioned	
**661**	Salicylic acid	Others	The microalga *Picochlorum* sp. SBL2	Not mentioned	[123]
**662**	Coumaric acid	Others	The microalga *Picochlorum* sp. SBL2	Not mentioned	
**663**	Gallic acid	Others	The microalga *Picochlorum* sp. SBL2	Not mentioned	
**664**	Neoxanthin	Others	The microalgae *Picochlorum* sp. SBL2, *Nannochloris* sp. (SBL1 and SBL4) and *Desmochloris* SBL3.	Not mentioned	

No.	Compound	Class	Source (Marine algae)	Biological Activity	Ref
**665**	Violaxanthin	Others	The microalgae *Picochlorum* sp. SBL2, *Nannochloris* sp. (SBL1 and SBL4) and *Desmochloris* SBL3.	Not mentioned	
**666**	Zeaxanthin	Others	The microalgae *Picochlorum* sp. SBL2, *Nannochloris* sp. (SBL1 and SBL4) and *Desmochloris* SBL3.	Not mentioned	
**667**	Lutein	Others	The microalgae *Picochlorum* sp. SBL2, *Nannochloris* sp. (SBL1 and SBL4) and *Desmochloris* SBL3.	Not mentioned	
**668**	Β-Carotene	Others	The microalgae *Picochlorum* sp. SBL2, *Nannochloris* sp. (SBL1 and SBL4) and *Desmochloris* SBL3.	Not mentioned	
**669**	Canthaxanthin	Others	The microalga *Picochlorum* sp. SBL2.	Not mentioned	
**670**	Hurgadenyne	Others	The red alga *Laurencia obtusa*	Not mentioned	[124]
**671**	Hurgadol	Terpene	The red alga *Laurencia obtusa*	Not mentioned	
**672**	*β*-Snyderol acetate	Terpene	The red alga *Laurencia obtusa*	Not mentioned	
**673**	Unnamed	Polyketide	The red alga *Laurencia obtusa*	Inhibition of TNF-α (Panel A), IL-6 (Panel B) and TGF-β (Panel C) release in Carrageenan-stimulated PBMCs (at 10 µM)	[125]
**674**	Unnamed	Polyketide	The red alga *Laurencia obtusa*	Inhibition of TNF-α (Panel A), IL-6 (Panel B) and TGF-β (Panel C) release in Carrageenan-stimulated PBMCs (at 10 µM)	
**675**	Unnamed	Polyketide	The red alga *Laurencia obtusa*	Inhibition of TNF-α (Panel A), IL-6 (Panel B) and TGF-β (Panel C) release in Carrageenan-stimulated PBMCs (at 10 µM)	

No.	Compound	Class	Source (Echinoderms)	Biological Activity	Ref
**676**	Holothurin A	Terpene	The sea cucumber (Holothurian) *Pearsonothuria graeffei*	Not mentioned	[126]
**677**	Echinoside A	Terpene	The sea cucumber (Holothurian) *Pearsonothuria graeffei*	Not mentioned	

Table S2: Locations of collection of the Red Sea marine organisms

No.	Marine organism	Location of collection	Country
**1**	*Acanthella carteri (= Acantheila aurantiaca)*	Hanish islands	Yemen
**2**	*Acarnus cf. bergquistae*	Dahlak Island	Eritrea
**3**	*Acarnus wolffgangi*	Hurghada (eastern side of the Small Giftun Island)	Egypt
**4**	*Actinomycete* RA2 (associated to *Spheciospongia mastoidea*)	Ras Muhammad	Egypt
**5**	*Antipathes dichotoma*	Hakel area	Saudi Arabia
**6**	*Aplysia oculifera*	Safaga	Egypt
**7**	*Aplysina fistularis*	Sharm El-Sheikh	Egypt
**8**	*Aplysinella* sp.	Jazan	Saudi Arabia
**9**	*Aspergillus fumigatus*	Hurghada	Egypt
**10**	*Callyspongia aff. implexa*	Safaga	Egypt
**11**	*Callyspongia fistularis*	Hurghada	Egypt
**12**	*Callyspongia siphonella*	Hurghada	Egypt
**13**	*Callyspongia* sp.	Hurghada	Egypt
		Obhur	Saudi Arabia
		Hurghada at El-Gouna and Shaa'b south Giffton island	Egypt
**14**	*Chrysosporium lobatum,* (Isolated from the Sponge *Acanthella cavernosa*)	Eilat	Israel
**15**	*Cladiella pachyclados*	Hurghada	Egypt
**16**	*Cladosporium* sp. (Associated *to Niphates rowi*)	Aqaba	Jordan

No.	Marine organism	Location of collection	Country
**17**	*Clathria* sp.	Dahlak archipelago	Eritrea
**18**	*Colpomenia sinuosa*	Gulf of Eilat	Israel
**19**	*Desmochloris* sp.	Al-Lith	Saudi Arabia
**20**	*Diacarnus erythraeanus*	Hurghada	Egypt
		Hurghada (El Qusier, 120 km south)	Egypt
**21**	*Dictyota dichotoma*	Ras Gharib on Suez-Gulf	Egypt
**22**	*Didemnum* sp.	Near Obhur	Saudi Arabia
		Sharm El-Sheikh	Egypt
**23**	*Dragmacidon coccinea*	Hurghada	Egypt
**24**	*Dysidea cinereal*	Eilat	Israel
**25**	*Dysidea herbacea*	Massawa	Eritrea
**26**	*Dysidea* sp.	Massawa	Eritrea
**27**	*Erylus lendenfeldi*	Hurghada	Egypt
**28**	*Erylusb* sp.	Dahlak archipelago	Eritrea
**29**	*Fusarium equiseti* (from the algae *Padina pavonica*)	Hurghada	Egypt
**30**	*Grayella cyatophora*	Near Djibouti	Djibouti
**31**	*Halichondria* sp.	Dahlak archipelago	Eritrea
**32**	*Haliclona sp.*	Gulf of Eilat	Israel
**33**	*Hemimycale arabica*	Jazan (Ghurab, north side)	Saudi Arabia

No.	Marine organism	Location of collection	Country
**34**	*Hexabranchus sanguineus*	Hurghada	Egypt
**35**	*Hippospongia sp.*	Hurghada	Egypt
**36**	*Hyrtios erectus*	Hurghada (El Quseir, 120 km south)	Egypt
		Hurghada	Egypt
		Sharm El-Sheikh	Egypt
		Jeddah	Saudi Arabia
		Safaga	Egypt
**37**	*Hyrtios sp.*	Hurghada	Egypt
**38**	*Jania adhaerens*	Al-Shoaiba coast	Saudi Arabia
**39**	*Jania rubens*	Sharm El-Sheikh	Egypt
**40**	*Lamellodysidea herbacea*	Ardoukoba	Djibouti
**41**	*Laurencia obtusa*	Jeddah	Saudi Arabia
		Hurghada	Egypt
		Salman Gulf, north of Jeddah	Saudi Arabia
**42**	*Laurenica spectabilis*	Ras-Gharib	Egypt
**43**	*Leptolyngbya* sp.	SS Thistlegorm shipwreck near Ras Muhammad	Egypt
**44**	*Leucetta cf chagosensis*	Hurghada	Egypt
**45**	*Litophyton arboretum*	Gulf of Eilat	Israel
**46**	*Litophyton arboreum*	Sharm El-Sheikh	Egypt

No.	Marine organism	Location of collection	Country
**47**	*Lobophyton crissum*	Sharm El-Sheikh	Egypt
**48**	*Lytocarpus philippinus*	Gulf of Eilat	Israel
**49**	MF003 *(fungus)*	El Gouna	Egypt
**50**	*Moorea producens*	Gulf of Aqaba near Sharm el-Sheikh	Egypt
		Near Obhur	Saudi Arabia
		Jeddah	Saudi Arabia
**51**	MR2012 *(fungus)*	Hurghada	Egypt
**52**	*Mycale euplectellioides*	Hurghada	Egypt
		Sharm El-Sheikh	Egypt
**53**	*Nannochloris* sp.	Al-Lith	Saudi Arabia
**54**	*Negombata corticate*	Safaga	Egypt
**55**	*Negombata magnifica*	Eilat	Israel
		Hurghada	Egypt
**56**	*Niphates* sp.	Hurghada at El-Gouna and Shaa'b south Giffton island	Egypt
		Eilat	Israel
**57**	*Nocardiopsis sp.* UR67 strain (associated with the marine sponge *Callyspongi*a sp)	Ras Muhammad	Egypt
**58**	*Okeania* sp.	Algetah Alkabira reef near Jeddah	Saudi Arabia
**59**	*Padina pavoniav*	Hurghada	Egypt
**60**	*Palythoa tuberculosa*	Hurghada	Egypt

No.	Marine organism	Location of collection	Country
**61**	*Parerythropodium fulvum fulvum*	Gulf of Eilat	Israel
**62**	*Pearsonothuria graeffei*	Gulf of Aqaba	Egypt
**63**	*Pfilocaulis spiculifer*	Dahlak archipelago	Eritrea
**64**	*Phyllospongia lamellose*	Hurghada	Egypt
**65**	*Picochlorum* sp.	Al-Lith	Saudi Arabia
**66**	*Pseudoceratina arabica*	Sharm El-Sheikh	Egypt
		Hurghada	Egypt
		Anas Reef off Obhur	Saudi Arabia
		Hurghada	Egypt
**67**	*Ptilocaulis spiculifer*	Dahlak archipelago	Eritrea
**68**	*Raspailia* sp.	Dahlak archipelago	Eritrea
**69**	*Rhytisma fulvum fulvum*	Hurghada	Egypt
**70**	*Sarcophyton auritum*	Safaga	Egypt
**71**	*Sarcophyton glaucum*	Hurghada	Egypt
		Jeddah	Saudi Arabia
		Sharm El-Sheikh	Egypt
**72**	*Sarcophyton* sp.	Hurghada	Egypt
**73**	*Sarcophyton trocheliophorum*	Hurghada	Egypt
		Gulf of Eilat	Israel

No.	Marine organism	Location of collection	Country
**74**	*Sargassum asperifolium*	Hurghada	Egypt
**75**	*Sargassum subrepandum*	Sharm El-Sheikh	Egypt
**76**	*Scopulariopsis* sp.	Ain El-Sokhna area	Egypt
**77**	*Sinularia candidula*	Safaga	Egypt
**78**	*Sinularia gardineri*	Hurghada	Egypt
**79**	*Sinularia leptoclados*	Sharm El-Sheikh	Egypt
**80**	*Sinularia notanda*	Gulf of Eilat	Israel
**81**	*Sinularia polydactyla*	Hurghada	Egypt
**82**	*Siphonochalina siphonella*	Sharm Obhur, Jeddah	Saudi Arabia
		Hurghada	Egypt
		Gulf of Eilat	Israel
		Dahlak archipelago	Eritrea
**83**	*Smenospongia* sp.	Hurghada at El-Gouna and Shaa'b south Giffton island	Egypt
**84**	*Spheciospongia vagabunda*	Ras Muhammad	Egypt
**85**	*Streptomyces* sp.	Sharm El-Sheikh	Egypt
**86**	*Streptomyces* sp. SP9 (associated to *Pseudoceratina arabica*)	Ras Muhammad	Egypt
**87**	*Stylissa carteri*	Hurghada	Egypt
**88**	*Stylissa* sp.	Hurghada at El-Gouna and Shaa'b south Giffton island	Egypt
**89**	*Suberea mollis*	Hurghada	Egypt

No.	Marine organism	Location of collection	Country
**90**	*Suberea* sp.	Yanbu	Saudi Arabia
**91**	*Thalassodendron ciliatum*	Magawish city near Hurghada	Egypt
		Safaga	Egypt
**92**	*Theonella swinhoei*	Hurghada	Egypt
**93**	*Toxiclona toxius*	Shaag Rock, in the Gulf of Suez,	Egypt
**94**	*Ulva lactuca*	Sharm El-Sheikh	Egypt
**95**	*Vibrio* sp.	Aqaba	Jordan
**96**	*Xenia lilielae*	Gulf of Eilat	Israel
**97**	*Xenia macrospiculata*	Gulf of Eilat	Israel
**98**	*Xenia obscuronafa*	Gulf of Suez	Egypt
**99**	*Xestospongia exigua*	Bayadha, 4 miles north of Jeddah	Saudi Arabia
**100**	*Xestospongia testudinaria*	Jazan (Ghurab Reef)	Saudi Arabia

Table S3: Taxonomy of marine organisms collected from the Red Sea

No.	Marine organism	Lineage	Type
**1**	*Aaptos aaptos*	Eukaryota; Opisthokonta; Metazoa; Porifera; Demospongiae; Heteroscleromorpha; Suberitida; Suberitidae; Aaptos	Sponge
**2**	*Acarnus* cf. *bergquistae*	Eukaryota; Opisthokonta; Metazoa; Porifera; Demospongiae; Heteroscleromorpha; Poecilosclerida; Acarnidae	Sponge
**3**	*Acarnus wolffgangi*	Eukaryota; Opisthokonta; Metazoa; Porifera; Demospongiae; Heteroscleromorpha; Poecilosclerida; Acarnidae	Sponge
**4**	*Actinokineospora* sp. strain EG49	Bacteria; Terrabacteria group; Actinobacteria; Actinobacteria; Pseudonocardiales; Pseudonocardiaceae	High GC gram positive bacterium
**5**	*Actinokineospora spheciospongiae*	Bacteria; Terrabacteria group; Actinobacteria; Actinobacteria; Pseudonocardiales; Pseudonocardiaceae	High GC gram positive bacterium
**6**	*Actinokineospora spheciospongiae* sp. nov	Bacteria; Terrabacteria group; Actinobacteria; Actinobacteria; Pseudonocardiales; Pseudonocardiaceae	High GC gram positive bacterium
**7**	*Actinomycete RA2*	Bacteria; Terrabacteria group; Actinobacteria; Actinobacteria; Actinomycetales; unclassified Actinomycetales; unclassified Actinomycetales	High GC gram positive bacterium
**8**	*Antipathes dichotoma*	Eukaryota; Opisthokonta; Metazoa; Eumetazoa; Cnidaria; Anthozoa; Hexacorallia; Antipatharia; Antipathidae; Antipathes	Black coral
**9**	*Aplysia oculifera*	Eukaryota; Opisthokonta; Metazoa; Eumetazoa; Bilateria; Protostomia; Lophotrochozoa; Mollusca; Gastropoda; Heterobranchia; Euthyneura; Euopisthobranchia; Aplysiida; Aplysioidea; Aplysiidae; Aplysia	Gastropod
**10**	*Aplysinella* sp.	Eukaryota; Opisthokonta; Metazoa; Porifera; Demospongiae; Verongimorpha; Verongiida; Aplysinellidae	Sponge
**11**	*Aspergillus fumigatus*	Eukaryota; Opisthokonta; Fungi; Dikarya; Ascomycota; saccharomyceta; Pezizomycotina; leotiomyceta; Eurotiomycetes; Eurotiomycetidae; Eurotiales; Aspergillaceae; Aspergillus	Fungus
**12**	*Axinella weltneri*	Eukaryota; Opisthokonta; Metazoa; Porifera; Demospongiae; Heteroscleromorpha; Axinellida; Axinellidae	Sponge
**13**	*Callyspongia aff. implexa*	Eukaryota; Opisthokonta; Metazoa; Porifera; Demospongiae; Heteroscleromorpha; Haplosclerida; Callyspongiidae	Sponge
**14**	*Callyspongia fistularis*	Eukaryota; Opisthokonta; Metazoa; Porifera; Demospongiae; Heteroscleromorpha; Haplosclerida; Callyspongiidae	Sponge

No.	Marine organism	Lineage	Type
**15**	*Callyspongia siphonella (Siphonochalina siphonella)*	Eukaryota; Opisthokonta; Metazoa; Porifera; Demospongiae; Heteroscleromorpha; Haplosclerida; Callyspongiidae	Sponge
**16**	*Callyspongia* sp.	Eukaryota; Opisthokonta; Metazoa; Porifera; Demospongiae; Heteroscleromorpha; Haplosclerida; Callyspongiidae	Sponge
**17**	*Callyspongia* species	Eukaryota; Opisthokonta; Metazoa; Porifera; Demospongiae; Heteroscleromorpha; Haplosclerida; Callyspongiidae	Sponge
**18**	*Chrysosporium lobatum* TM-237-S5	Eukaryota; Opisthokonta; Fungi; Dikarya; Ascomycota; saccharomyceta; Pezizomycotina; leotiomyceta; Eurotiomycetes; Eurotiomycetidae; Onygenales; Onygenales incertae sedis; Chrysosporium	Fungus
**19**	*Cladiella pachyclados*	Eukaryota; Opisthokonta; Metazoa; Eumetazoa; Cnidaria; Anthozoa; Octocorallia; Alcyonacea; Alcyoniina; Alcyoniidae; Cladiella	Soft coral
**20**	*Cladosporium* sp.	Eukaryota; Opisthokonta; Fungi; Dikarya; Ascomycota; saccharomyceta; Pezizomycotina; leotiomyceta; dothideomyceta; Dothideomycetes; Dothideomycetidae; Capnodiales; Cladosporiaceae	Fungus
**21**	*Clathria* sp.	Eukaryota; Opisthokonta; Metazoa; Porifera; Demospongiae; Heteroscleromorpha; Poecilosclerida; Microcionidae; Clathria	Sponge
**22**	*Colpomenia sinuosa*	Eukaryota; Stramenopiles; PX clade; Phaeophyceae; Ectocarpales; Scytosiphonaceae; Colpomenia	Brown alga
**23**	*Crella cyathophora (Grayella cyatophora)*	Eukaryota; Opisthokonta; Metazoa; Porifera; Demospongiae; Heteroscleromorpha; Poecilosclerida; Crellidae; Crella	Sponge
**24**	*Deuteromycete* sp. MF 003	Undefined	Fungus
**25**	*Diacarnus erythraeanus*	Eukaryota; Opisthokonta; Metazoa; Porifera; Demospongiae; Heteroscleromorpha; Poecilosclerida; Podospongiidae; Diacarnus	Sponge
**26**	*Dictyota dichotoma*	Eukaryota; Stramenopiles; PX clade; Phaeophyceae; Dictyotales; Dictyotaceae; Dictyota	Brown alga
**27**	*Didemnum* sp.	Eukaryota; Opisthokonta; Metazoa; Eumetazoa; Bilateria; Deuterostomia; Chordata; Tunicata; Ascidiacea; Enterogona; Aplousobranchia; Didemnidae	Tunicate
**28**	*Dolabella auricularia*	Eukaryota; Opisthokonta; Metazoa; Eumetazoa; Bilateria; Protostomia; Spiralia; Lophotrochozoa; Mollusca; Gastropoda; Heterobranchia; Euthyneura; Euopisthobranchia; Aplysiida; Aplysioidea; Ap lysiidae; Dolabella	Gastropod
**29**	*Dragmacidon coccineum (Dragmacidon coccinea)*	Eukaryota; Opisthokonta; Metazoa; Porifera; Demospongiae; Heteroscleromorpha; Axinellida; Axinellidae; Dragmacidon	Sponge

No.	Marine organism	Lineage	Type
**30**	*Dysidea* sp.	Eukaryota; Opisthokonta; Metazoa; Porifera; Demospongiae; Keratosa; Dictyoceratida; Dysideidae; Dysidea	Sponge
**31**	*Echinoclathria gibbosa*	Eukaryota; Opisthokonta; Metazoa; Porifera; Demospongiae; Heteroscleromorpha; Poecilosclerida; Microcionidae	Sponge
**32**	*Erylus cf. lendenfeidi*	Eukaryota; Opisthokonta; Metazoa; Porifera; Demospongiae; Heteroscleromorpha; Tetractinellida; Astrophorina; Geodiidae; Erylus	Sponge
**33**	*Fusarium equiseti*	Eukaryota; Opisthokonta; Fungi; Dikarya; Ascomycota; saccharomyceta; Pezizomycotina; leotiomy ceta; sordariomyceta; Sordariomycetes; Hypocreomycetidae; Hypocreales; Nectriaceae; Fusarium; F usarium incarnatum-equiseti species complex	Fungus
**34**	*Halichondria* sp.	Eukaryota; Opisthokonta; Metazoa; Porifera; Demospongiae; Heteroscleromorpha; Suberitida; Halichondriidae; Halichondria	Sponge
**35**	*Haliclona* sp.	Eukaryota; Opisthokonta; Metazoa; Porifera; Demospongiae; Heteroscleromorpha; Haplosclerida; Chalinidae; Haliclona	Sponge
**36**	*Haliclona toxia (Toxiclona toxius)*	Eukaryota; Opisthokonta; Metazoa; Porifera; Demospongiae; Heteroscleromorpha; Haplosclerida; Chalinidae; Haliclona; Gellius	Sponge
**37**	*Hemimycale arabica*	Eukaryota; Opisthokonta; Metazoa; Porifera; Demospongiae; Heteroscleromorpha; Poecilosclerida; Hymedesmiidae; Hemimycale	Sponge
**38**	*Hexabranchus sanguineus*	Eukaryota; Opisthokonta; Metazoa; Eumetazoa; Bilateria; Protostomia; Lophotrochozoa; Mollusca; Gastropoda; Heterobranchia; Euthyneura; Nudipleura; Nudibranchia; Doridina; Eudoridoidea; Hexabranchidae; Hexabranchus	Gastropod
**39**	*Hippospongia* sp.	Eukaryota; Opisthokonta; Metazoa; Porifera; Demospongiae; Keratosa; Dictyoceratida; Spongiidae; Hippospongia	Sponge
**40**	*Hyattella intestinalis*	Eukaryota; Opisthokonta; Metazoa; Porifera; Demospongiae; Keratosa; Dictyoceratida; Spongiidae; Hyattella	Sponge
**41**	*Hyrtios erectus*	Eukaryota; Opisthokonta; Metazoa; Porifera; Demospongiae; Keratosa; Dictyoceratida; Thorectidae; Hyrtios	Sponge
**42**	*Hyrtios* sp.	Eukaryota; Opisthokonta; Metazoa; Porifera; Demospongiae; Keratosa; Dictyoceratida; Thorectidae; Hyrtios	Sponge
**43**	*Jania adhaerens*	Eukaryota; Rhodophyta; Florideophyceae; Corallinophycidae; Corallinales; Corallinaceae; Corallinoideae; Jania	Red alga
**44**	*Lamellodysidea herbacea (Dysidea herbacea)*	Eukaryota; Opisthokonta; Metazoa; Porifera; Demospongiae; Keratosa; Dictyoceratida; Dysideidae; Lamellodysidea	Sponge

No.	Marine organism	Lineage	Type
**45**	*Laurencia obtusa*	Eukaryota; Rhodophyta; Florideophyceae; Rhodymeniophycidae; Ceramiales; Rhodomelaceae; Laurencieae; Laurencia	Red alga
**46**	*Leptolyngbya* sp.	Bacteria; Terrabacteria group; Cyanobacteria/Melainabacteria group; Cyanobacteria; Synechococcales; Leptolyngbyaceae; Leptolyngbya	Cyanobacterium
**47**	*Leucetta* cf *chagosensis*	Eukaryota; Opisthokonta; Metazoa; Porifera; Calcarea; Calcinea; Clathrinida; Leucettidae; Leucetta	Sponge
**48**	*Litophyton arboreum*	Eukaryota; Opisthokonta; Metazoa; Eumetazoa; Cnidaria; Anthozoa; Octocorallia; Alcyonacea; Alcyoniina; Nephtheidae; Litophyton	Soft coral
**49**	*Macrorhynchia philippina (Lytocarpus philippinus)*	Eukaryota; Opisthokonta; Metazoa; Eumetazoa; Cnidaria; Hydrozoa; Hydroidolina; Leptothecata; Aglaopheniidae; Macrorhynchia	Hydroid
**50**	*Micrococcus* sp. EG45	Bacteria; Terrabacteria group; Actinobacteria; Actinobacteria; Micrococcales; Micrococcaceae; Micrococcus	High GC gram positive bacterium
**51**	*Moorea producens*	Bacteria; Terrabacteria group; Cyanobacteria/Melainabacteria group; Cyanobacteria; Oscillatoriophycideae; Oscillatoriales; Oscillatoriaceae; Moorea	Cyanobacterium
**52**	*Mycale euplectellioides*	Eukaryota; Opisthokonta; Metazoa; Porifera; Demospongiae; Heteroscleromorpha; Poecilosclerida; Mycalidae; Mycale	Sponge
**53**	*Negombata corticata*	Eukaryota; Opisthokonta; Metazoa; Porifera; Demospongiae; Heteroscleromorpha; Poecilosclerida; Latrunculiidae; Negombata	Sponge
**54**	*Negombata magnifica*	Eukaryota; Opisthokonta; Metazoa; Porifera; Demospongiae; Heteroscleromorpha; Poecilosclerida; Latrunculiidae; Negombata	Sponge
**55**	*Niphates* sp.	Eukaryota; Opisthokonta; Metazoa; Porifera; Demospongiae; Heteroscleromorpha; Haplosclerida; Niphatidae; Niphates	Sponge
**56**	*Nocardia* sp. ALAA 2000	Bacteria; Terrabacteria group; Actinobacteria; Actinobacteria; Corynebacteriales; Nocardiaceae; Nocardia	High GC gram positive bacterium
**57**	*Nocardiopsis* sp. UR67	Bacteria; Terrabacteria group; Actinobacteria; Actinobacteria; Streptosporangiales; Nocardiopsaceae; Nocardiopsis	High GC gram positive bacterium
**58**	*Okeania* sp.	Bacteria; Terrabacteria group; Cyanobacteria/Melainabacteria group; Cyanobacteria; Oscillatoriophycideae; Oscillatoriales; Oscillatoriaceae; Okeania	cyanobacteria
**59**	*Ovabunda macrospiculata (Xenia macrospiculata)*	Eukaryota; Opisthokonta; Metazoa; Eumetazoa; Cnidaria; Anthozoa; Octocorallia; Alcyonacea; Alcyoniina; Xeniidae	Soft coral

No.	Marine organism	Lineage	Type
**60**	*Ovabunda macrospiculata (Xenia macrospiculata)* and *Ovabunda obscuronata (Xenia obscuronata)*	Eukaryota; Opisthokonta; Metazoa; Eumetazoa; Cnidaria; Anthozoa; Octocorallia; Alcyonacea; Alcyoniina; Xeniidae	Soft coral
**61**	*Ovabunda macrospiculata (Xenia macrospiculata), Ovabunda obscuronata (Xenia obscuronata),* and *Xenia lillieae*	Eukaryota; Opisthokonta; Metazoa; Eumetazoa; Cnidaria; Anthozoa; Octocorallia; Alcyonacea; Alcyoniina; Xeniidae	Soft coral
**62**	*Ovabunda obscuronata (Xenia obscuronata)*	Eukaryota; Opisthokonta; Metazoa; Eumetazoa; Cnidaria; Anthozoa; Octocorallia; Alcyonacea; Alcyoniina; Xeniidae	Soft coral
**63**	*Padina pavonica*	Eukaryota; Stramenopiles; PX clade; Phaeophyceae; Dictyotales; Dictyotaceae; Padina	Brown alga
**64**	*Palythoa tuberculosa*	Eukaryota; Opisthokonta; Metazoa; Eumetazoa; Cnidaria; Anthozoa; Hexacorallia; Zoantharia; Sphenopidae; Palythoa	Zoanthid
**65**	*Pearsonothuria graeffei*	Eukaryota; Opisthokonta; Metazoa; Eumetazoa; Bilateria; Deuterostomia; Echinodermata; Eleutherozoa; Echinozoa; Holothuroidea; Aspidochirotacea; Aspidochirotida; Holothuriidae; Pearsonothuria	Sea cucumber
**66**	*Penicillium chrysogenum*	Eukaryota; Opisthokonta; Fungi; Dikarya; Ascomycota; saccharomyceta; Pezizomycotina; leotiomy ceta; Eurotiomycetes; Eurotiomycetidae; Eurotiales; Aspergillaceae; Penicillium	Fungus
**67**	*Petrosia* sp.	Eukaryota; Opisthokonta; Metazoa; Porifera; Demospongiae; Heteroscleromorpha; Haplosclerida; Petrosiidae; Petrosia	Sponge
**68**	*Phyllospongia lamellosa*	Eukaryota; Opisthokonta; Metazoa; Porifera; Demospongiae; Keratosa; Dictyoceratida; Thorectidae; Phyllospongia	Sponge
**69**	*Picochlorum* sp.	Eukaryota; Viridiplantae; Chlorophyta; Trebouxiophyceae; Trebouxiophyceae incertae sedis; Picochlorum	Green alga
**70**	*Picochlorum* sp. SBL2	Eukaryota; Viridiplantae; Chlorophyta; Trebouxiophyceae; Trebouxiophyceae incertae sedis; Picochlorum	Green alga
**71**	*Picochlorum* sp. SBL2, *Nannochloris* sp. (SBL1 and SBL4), and *Desmochloris* SBL3	Eukaryota; Viridiplantae; Chlorophyta; Trebouxiophyceae; Trebouxiophyceae incertae sedis; Picochlorum, and Eukaryota; Viridiplantae; Chlorophyta; Trebouxiophyceae; Chlorellales; Chlorellaceae; Nannochloris; unclassified Nannochloris, and Eukaryota; Viridiplantae; Chlorophyta; Ulvophyceae; OUU clade; Ulvales; Ulvales incertae sedis	Green alga
**72**	*Pseudoceratina arabica*	Eukaryota; Opisthokonta; Metazoa; Porifera; Demospongiae; Verongimorpha; Verongiida; Pseudoceratinidae; Pseudoceratina	Sponge

No.	Marine organism	Lineage	Type
**73**	*Ptilocaulis spiculifer*	Eukaryota; Opisthokonta; Metazoa; Porifera; Demospongiae; Heteroscleromorpha; Axinellida; Axinellidae; Ptilocaulis	Sponge
**74**	*Raspailia* sp.	Eukaryota; Opisthokonta; Metazoa; Porifera; Demospongiae; Heteroscleromorpha; Axinellida; Raspailiidae; Raspailiinae; Raspailia	Sponge
**75**	*Rhytisma fulvum fulvum (Parerythropodium fulvum fulvum)*	Eukaryota; Opisthokonta; Metazoa; Eumetazoa; Cnidaria; Anthozoa; Octocorallia; Alcyonacea; Alcyoniina; Alcyoniidae; Rhytisma; Rhytisma fulvum	Soft coral
**76**	*Sarcophyton auritum*	Eukaryota; Opisthokonta; Metazoa; Eumetazoa; Cnidaria; Anthozoa; Octocorallia; Alcyonacea; Alcyoniina; Alcyoniidae; Sarcophyton	Soft coral
**77**	*Sarcophyton glaucum*	Eukaryota; Opisthokonta; Metazoa; Eumetazoa; Cnidaria; Anthozoa; Octocorallia; Alcyonacea; Alcyoniina; Alcyoniidae; Sarcophyton	Soft coral
**78**	*Sarcophyton glaucum* and *Lobophytum crassum*	Eukaryota; Opisthokonta; Metazoa; Eumetazoa; Cnidaria; Anthozoa; Octocorallia; Alcyonacea; Alcyoniina; Alcyoniidae; Sarcophyton and Eukaryota; Opisthokonta; Metazoa; Eumetazoa; Cnidaria; Anthozoa; Octocorallia; Alcyonacea; Alcyoniina; Alcyoniidae; Lobophytum	Soft coral
**79**	*Sarcophyton* sp.	Eukaryota; Opisthokonta; Metazoa; Eumetazoa; Cnidaria; Anthozoa; Octocorallia; Alcyonacea; Alcyoniina; Alcyoniidae; Sarcophyton	Soft coral
**80**	*Sarcophyton trocheliophorum*	Eukaryota; Opisthokonta; Metazoa; Eumetazoa; Cnidaria; Anthozoa; Octocorallia; Alcyonacea; Alcyoniina; Alcyoniidae; Sarcophyton	Soft coral
**81**	*Sarcophyton trocheliophorum and Litophyton arboreum*	Eukaryota; Opisthokonta; Metazoa; Eumetazoa; Cnidaria; Anthozoa; Octocorallia; Alcyonacea; Alcyoniina; Alcyoniidae; Sarcophyton and Eukaryota; Opisthokonta; Metazoa; Eumetazoa; Cnidaria; Anthozoa; Octocorallia; Alcyonacea; Alcyoniina; Nephtheidae; Litophyton	Soft coral
**82**	*Sargassum asperifolium*	Eukaryota; Stramenopiles; PX clade; Phaeophyceae; Fucales; Sargassaceae; Sargassum	Brown alga
**83**	*Scopulariopsis* sp.	Eukaryota; Opisthokonta; Fungi; Dikarya; Ascomycota; saccharomyceta; Pezizomycotina; leotiomyceta; sordariomyceta; Sordariomycetes; Hypocreomycetidae; Microascales; Microascaceae; Scopulariopsis	Fungus
**84**	*Sinularia candidula*	Eukaryota; Opisthokonta; Metazoa; Eumetazoa; Cnidaria; Anthozoa; Octocorallia; Alcyonacea; Alcyoniina; Alcyoniidae; Sinularia	Soft coral
**85**	*Sinularia gardineri*	Eukaryota; Opisthokonta; Metazoa; Eumetazoa; Cnidaria; Anthozoa; Octocorallia; Alcyonacea; Alcyoniina; Alcyoniidae; Sinularia	Soft coral
**86**	*Sinularia notanda*	Eukaryota; Opisthokonta; Metazoa; Eumetazoa; Cnidaria; Anthozoa; Octocorallia; Alcyonacea; Alcyoniina; Alcyoniidae; Sinularia	Soft coral

No.	Marine organism	Lineage	Type
**87**	*Sinularia polydactyla*	Eukaryota; Opisthokonta; Metazoa; Eumetazoa; Cnidaria; Anthozoa; Octocorallia; Alcyonacea; Alcyoniina; Alcyoniidae; Sinularia	Soft coral
**88**	*Smenospongia* sp.	Eukaryota; Opisthokonta; Metazoa; Porifera; Demospongiae; Keratosa; Dictyoceratida; Thorectidae; Smenospongia	Sponge
**89**	*Streptomyces* sp. Did-27	Bacteria; Terrabacteria group; Actinobacteria; Actinobacteria; Streptomycetales; Streptomycetaceae; Streptomyces	High GC gram positive bacterium
**90**	*Streptomyces* sp. EGY1	Bacteria; Terrabacteria group; Actinobacteria; Actinobacteria; Streptomycetales; Streptomycetaceae; Streptomyces	High GC gram positive bacterium
**91**	*Streptomyces* sp. Hedaya48	Bacteria; Terrabacteria group; Actinobacteria; Actinobacteria; Streptomycetales; Streptomycetaceae; Streptomyces	High GC gram positive bacterium
**92**	*Streptomyces* sp. SP9	Bacteria; Terrabacteria group; Actinobacteria; Actinobacteria; Streptomycetales; Streptomycetaceae; Streptomyces	High GC gram positive bacterium
**93**	*Stylissa carteri (Acanthella carteri)*	Eukaryota; Opisthokonta; Metazoa; Porifera; Demospongiae; Heteroscleromorpha; Axinellida; Axinellidae; Stylissa	Sponge
**94**	*Suberea mollis*	Eukaryota; Opisthokonta; Metazoa; Porifera; Demospongiae; Verongimorpha; Verongiida; Aplysinellidae; Suberea	Sponge
**95**	*Suberea* sp.	Eukaryota; Opisthokonta; Metazoa; Porifera; Demospongiae; Verongimorpha; Verongiida; Aplysinellidae; Suberea	Sponge
**96**	*Thalassodendron ciliatum*	Eukaryota; Viridiplantae; Streptophyta; Streptophytina; Embryophyta; Tracheophyta; Euphyllophyta; Spermatophyta; Magnoliopsida; Mesangiospermae; Liliopsida; Alismatales; Cymodoceaceae; Thalassodendron	Sea grass
**97**	*Theonella swinhoei*	Eukaryota; Opisthokonta; Metazoa; Porifera; Demospongiae; Heteroscleromorpha; Tetractinellida; Astrophorina; Theonellidae; Theonella	Sponge
**98**	*Vibrio* sp.	Bacteria; Proteobacteria; Gammaproteobacteria; Vibrionales; Vibrionaceae; Vibrio	γ-proteobacterium
**99**	*Xenia lillieae*	Eukaryota; Opisthokonta; Metazoa; Eumetazoa; Cnidaria; Anthozoa; Octocorallia; Alcyonacea; Alcyoniina; Xeniidae	Soft coral
**100**	*Xestospongia exigua*	Eukaryota; Opisthokonta; Metazoa; Porifera; Demospongiae; Heteroscleromorpha; Haplosclerida; P etrosiidae; Neopetrosia	Sponge
**101**	*Xestospongia testudinaria*	Eukaryota; Opisthokonta; Metazoa; Porifera; Demospongiae; Heteroscleromorpha; Haplosclerida; Petrosiidae; Xestospongia	Sponge

References

1. Lopez, J.A.; Al-Lihaibi, S.S.; Alarif, W.M.; Abdel-Lateff, A.; Nogata, Y.; Washio, K.; Morikawa, M.; Okino, T. Wewakazole b, a cytotoxic cyanobactin from the cyanobacterium moorea producens collected in the red sea. *J Nat Prod* **2016**, *79*, 1213-1218.
2. Shaala, L.A.; Youssef, D.T.; Badr, J.M.; Harakeh, S.M. Bioactive 2(1h)-pyrazinones and diketopiperazine alkaloids from a tunicate-derived actinomycete streptomyces sp. *Molecules* **2016**, *21*.
3. Youssef, D.T.; Shaala, L.A.; Mohamed, G.A.; Ibrahim, S.R.; Banjar, Z.M.; Badr, J.M.; McPhail, K.L.; Risinger, A.L.; Mooberry, S.L. 2,3-seco-2,3-dioxo-lyngbyatoxin a from a red sea strain of the marine cyanobacterium moorea producens. *Nat Prod Res* **2015**, *29*, 703-709.
4. Al-Zereini, W.; Fotso Fondja Yao, C.B.; Laatsch, H.; Anke, H. Aqabamycins a-g: Novel nitro maleimides from a marine vibrio species. I. Taxonomy, fermentation, isolation and biological activities. *J Antibiot (Tokyo)* **2010**, *63*, 297-301.
5. Shaala, L.A.; Youssef, D.T.A.; McPhail, K.L.; Elbandy, M. Malyngamide 4, a new lipopeptide from the red sea marine cyanobacterium moorea producens (formerly lyngbya majuscula). *Phytochemistry Letters* **2013**, *6*, 183-188.
6. Youssef, D.T.; Ibrahim, S.R.; Shaala, L.A.; Mohamed, G.A.; Banjar, Z.M. New cerebroside and nucleoside derivatives from a red sea strain of the marine cyanobacterium moorea producens. *Molecules* **2016**, *21*, 324.
7. El-Gendy, M.M.; El-Bondkly, A.M. Production and genetic improvement of a novel antimycotic agent, saadamycin, against dermatophytes and other clinical fungi from endophytic streptomyces sp. Hedaya48. *J Ind Microbiol Biotechnol* **2010**, *37*, 831-841.
8. Abdelfattah, M.S.; Elmallah, M.I.Y.; Mohamed, A.A.; Ishibashi, M. Sharkquinone, a new ana-quinonoid tetracene derivative from marine-derived streptomyces sp. Egy1 with trail resistance-overcoming activity. *J Nat Med* **2017**, *71*, 564-569.
9. Abdelfattah, M.S.; Elmallah, M.I.Y.; Faraag, A.H.I.; Hebishy, A.M.S.; Ali, N.H. Heliomycin and tetracinomycin d: Anthraquinone derivatives with histone deacetylase inhibitory activity from marine sponge-associated streptomyces sp. Sp9. *3 Biotech* **2018**, *8*, 282.
10. Ibrahim, A.H.; Attia, E.Z.; Hajjar, D.; Anany, M.A.; Desoukey, S.Y.; Fouad, M.A.; Kamel, M.S.; Wajant, H.; Gulder, T.A.M.; Abdelmohsen, U.R. New cytotoxic cyclic peptide from the marine sponge-associated nocardiopsis sp. Ur67. *Marine Drugs* **2018**, *16*, 290.
11. Eltamany, E.E.; Abdelmohsen, U.R.; Ibrahim, A.K.; Hassanean, H.A.; Hentschel, U.; Ahmed, S.A. New antibacterial xanthone from the marine sponge-derived micrococcus sp. Eg45. *Bioorg Med Chem Lett* **2014**, *24*, 4939-4942.
12. Thornburg, C.C.; Thimmaiah, M.; Shaala, L.A.; Hau, A.M.; Malmo, J.M.; Ishmael, J.E.; Youssef, D.T.; McPhail, K.L. Cyclic depsipeptides, grassypeptolides d and e and ibu-epidemethoxylyngbyastatin 3, from a red sea leptolyngbya cyanobacterium. *J Nat Prod* **2011**, *74*, 1677-1685.
13. Thornburg, C.C.; Cowley, E.S.; Sikorska, J.; Shaala, L.A.; Ishmael, J.E.; Youssef, D.T.; McPhail, K.L. Apratoxin h and apratoxin a sulfoxide from the red sea cyanobacterium moorea producens. *J Nat Prod* **2013**, *76*, 1781-1788.
14. Tawfike, A.; Attia, E.Z.; Desoukey, S.Y.; Hajjar, D.; Makki, A.A.; Schupp, P.J.; Edrada-Ebel, R.; Abdelmohsen, U.R. New bioactive metabolites from the elicited marine sponge-derived bacterium actinokineospora spheciospongiae sp. Nov. *AMB Express* **2019**, *9*, 12-12.
15. Abdelfattah, M.S.; Elmallah, M.I.Y.; Ebrahim, H.Y.; Almeer, R.S.; Eltanany, R.M.A.; Abdel Moneim, A.E. Prodigiosins from a marine sponge-associated actinomycete attenuate hcl/ethanol-induced gastric lesion via antioxidant and anti-inflammatory mechanisms. *PloS one* **2019**, *14*, e0216737.
16. Gesner, S.; Cohen, N.; Ilan, M.; Yarden, O.; Carmeli, S. Pandangolide 1a, a metabolite of the sponge-associated fungus cladosporium sp., and the absolute stereochemistry of pandangolide 1 and iso-cladospolide b. *Journal of Natural Products* **2005**, *68*, 1350-1353.

17. El-Gendy Bel, D.; Rateb, M.E. Antibacterial activity of diketopiperazines isolated from a marine fungus using t-butoxycarbonyl group as a simple tool for purification. *Bioorg Med Chem Lett* **2015**, *25*, 3125-3128.
18. Elnaggar, M.S.; Ebada, S.S.; Ashour, M.L.; Ebrahim, W.; Müller, W.E.G.; Mándi, A.; Kurtán, T.; Singab, A.; Lin, W.; Liu, Z.*, et al.* Xanthones and sesquiterpene derivatives from a marine-derived fungus scopulariopsis sp. *Tetrahedron* **2016**, *72*, 2411-2419.
19. Nawwar, M.; Hussein, S.; Ayoub, N.A.; Hashim, A.; Mernitz, G.; Cuypers, B.; Linscheid, M.; Lindequist, U. Deuteromycols a and b, two benzofuranoids from a red sea marine-derived deuteromycete sp. *Arch Pharm Res* **2010**, *33*, 1729-1733.
20. Hawas, U.W.; Al-Farawati, R.; Abou El-Kassem, L.T.; Turki, A.J. Different culture metabolites of the red sea fungus fusarium equiseti optimize the inhibition of hepatitis c virus ns3/4a protease (hcv pr). *Mar Drugs* **2016**, *14*.
21. Le Goff, G.; Lopes, P.; Arcile, G.; Vlachou, P.; Van Elslande, E.; Retailleau, P.; Gallard, J.-F.; Weis, M.; Benayahu, Y.; Fokialakis, N.*, et al.* Impact of the cultivation technique on the production of secondary metabolites by chrysosporium lobatum tm-237-s5, isolated from the sponge acanthella cavernosa. *Marine drugs* **2019**, *17*, 678.
22. Copmans, D.; Rateb, M.; Tabudravu, J.N.; Pérez-Bonilla, M.; Dirkx, N.; Vallorani, R.; Diaz, C.; Pérez del Palacio, J.; Smith, A.J.; Ebel, R.*, et al.* Zebrafish-based discovery of antiseizure compounds from the red sea: Pseurotin a2 and azaspirofuran a. *ACS Chemical Neuroscience* **2018**, *9*, 1652-1662.
23. Hawas, U.W.; Abou El-Kassem, L.T. Anticancer and antiviral diketopiperazine produced by the red sea endophytic fungus penicillium chrysogenum. *Letters in Organic Chemistry* **2019**, *16*, 409-414.
24. Talpir, R.; Rudi, A.; Kashman, Y.; Loya, Y.; Hizi, A. Three new sesquiterpene hydroquinones from marine origin. *Tetrahedron* **1994**, *50*, 4179-4184.
25. Alahdal, A.M.; Asfour, H.Z.; Ahmed, S.A.; Noor, A.O.; Al-Abd, A.M.; Elfaky, M.A.; Elhady, S.S. Anti-helicobacter, antitubercular and cytotoxic activities of scalaranes from the red sea sponge hyrtios erectus. *Molecules* **2018**, *23*, 978.
26. Elhady, S.S.; El-Halawany, A.M.; Alahdal, A.M.; Hassanean, H.A.; Ahmed, S.A. A new bioactive metabolite isolated from the red sea marine sponge hyrtios erectus. *Molecules* **2016**, *21*, 82.
27. Elhady, S.S.; Al-Abd, A.M.; El-Halawany, A.M.; Alahdal, A.M.; Hassanean, H.A.; Ahmed, S.A. Antiproliferative scalarane-based metabolites from the red sea sponge hyrtios erectus. *Mar Drugs* **2016**, *14*.
28. Ehrlich, H.; Shaala, L.A.; Youssef, D.T.A.; Zoltowska-Aksamitowska, S.; Tsurkan, M.; Galli, R.; Meissner, H.; Wysokowski, M.; Petrenko, I.; Tabachnick, K.R.*, et al.* Discovery of chitin in skeletons of non-verongiid red sea demosponges. *PLoS One* **2018**, *13*, e0195803.
29. Żółtowska-Aksamitowska, S.; Shaala, L.A.; Youssef, D.T.A.; Elhady, S.S.; Tsurkan, M.V.; Petrenko, I.; Wysokowski, M.; Tabachnick, K.; Meissner, H.; Ivanenko, V.N.*, et al.* First report on chitin in a non-verongiid marine demosponge: The mycale euplectellioides case. *Marine Drugs* **2018**, *16*, 68.
30. Shaala, L.A.; Asfour, H.Z.; Youssef, D.T.A.; Żółtowska-Aksamitowska, S.; Wysokowski, M.; Tsurkan, M.; Galli, R.; Meissner, H.; Petrenko, I.; Tabachnick, K.*, et al.* New source of 3d chitin scaffolds: The red sea demosponge pseudoceratina arabica (pseudoceratinidae, verongiida). *Marine drugs* **2019**, *17*, 92.
31. Shaala, L.A.; Youssef, D.T.A.; Badr, J.M.; Sulaiman, M.; Khedr, A.; El Sayed, K.A. Bioactive alkaloids from the red sea marine verongid sponge pseudoceratina arabica. *Tetrahedron* **2015**, *71*, 7837-7841.
32. El-Gamal, A.A.; Al-Massarani, S.M.; Shaala, L.A.; Alahdald, A.M.; Al-Said, M.S.; Ashour, A.E.; Kumar, A.; Abdel-Kader, M.S.; Abdel-Mageed, W.M.; Youssef, D.T. Cytotoxic compounds from the saudi red sea sponge xestospongia testudinaria. *Mar Drugs* **2016**, *14*.

33. O'Rourke, A.; Kremb, S.; Bader, T.M.; Helfer, M.; Schmitt-Kopplin, P.; Gerwick, W.H.; Brack-Werner, R.; Voolstra, C.R. Alkaloids from the sponge stylissa carteri present prospective scaffolds for the inhibition of human immunodeficiency virus 1 (hiv-1). *Mar Drugs* **2016**, *14*.
34. Youssef, D.T.; Shaala, L.A.; Alshali, K.Z. Bioactive hydantoin alkaloids from the red sea marine sponge hemimycale arabica. *Mar Drugs* **2015**, *13*, 6609-6619.
35. Shaala, L.A.; Youssef, D.T.; Badr, J.M.; Sulaiman, M.; Khedr, A. Bioactive secondary metabolites from the red sea marine verongid sponge suberea species. *Mar Drugs* **2015**, *13*, 1621-1631.
36. Shaala, L.A.; Youssef, D.T.; Sulaiman, M.; Behery, F.A.; Foudah, A.I.; Sayed, K.A. Subereamolline a as a potent breast cancer migration, invasion and proliferation inhibitor and bioactive dibrominated alkaloids from the red sea sponge pseudoceratina arabica. *Mar Drugs* **2012**, *10*, 2492-2508.
37. Abou-Shoer, M.I.; Shaala, L.A.; Youssef, D.T.A.; Badr, J.M.; Habib, A.-A.M. Bioactive brominated metabolites from the red sea sponge suberea mollis. *Journal of Natural Products* **2008**, *71*, 1464-1467.
38. Shaala, L.A.; Bamane, F.H.; Badr, J.M.; Youssef, D.T. Brominated arginine-derived alkaloids from the red sea sponge suberea mollis. *J Nat Prod* **2011**, *74*, 1517-1520.
39. Youssef, D.T.; Shaala, L.A.; Asfour, H.Z. Bioactive compounds from the red sea marine sponge hyrtios species. *Mar Drugs* **2013**, *11*, 1061-1070.
40. Badr, J.M.; Shaala, L.A.; Abou-Shoer, M.I.; Tawfik, M.K.; Habib, A.A. Bioactive brominated metabolites from the red sea sponge pseudoceratina arabica. *J Nat Prod* **2008**, *71*, 1472-1474.
41. Sauleau, P.; Retailleau, P.; Vacelet, J.; Bourguet-Kondracki, M.-L. New polychlorinated pyrrolidinones from the red sea marine sponge lamellodysidea herbacea. *Tetrahedron* **2005**, *61*, 955-963.
42. Isaacs, S.; Berman, R.; Kashman, Y.; Gebreyesus, T.; Yosief, T. New polyhydroxy sterols, dysidamides, and a dideoxyhexose from the sponge dysidea herbacea. *Journal of Natural Products* **1991**, *54*, 83-91.
43. Chuck Dunbar, D.; Rimoldi, J.M.; Clark, A.M.; Kelly, M.; Hamann, M.T. Anti-cryptococcal and nitric oxide synthase inhibitory imidazole alkaloids from the calcareous sponge leucetta cf chagosensis. *Tetrahedron* **2000**, *56*, 8795-8798.
44. Rudi, A.; Yosief, T.; Schleyer, M.; Kashman, Y. Several new isoprenoids from two marine sponges of the family axinellidae. *Tetrahedron* **1999**, *55*, 5555-5566.
45. Mancini, I.; Guella, G.; Pietra, F.; Amade, P. Hanishenols a-b, novel linear or methyl-branched glycerol enol ethers of the axinellid sponge acanthella carteri (= acanthella aurantiaca) from the hanish islands, southern red sea. *Tetrahedron* **1997**, *53*, 2625-2628.
46. Goobes, R.; Rudi, A.; Kashman, Y.; Ilan, M.; Loya, Y. Three new glycolipids from a red sea sponge of the genus erylus. *Tetrahedron* **1996**, *52*, 7921-7928.
47. Isaacs, S.; Kashman, Y.; Loya, S.; Hizi, A.; Loya, Y. Petrosynol and petrosolic acid, two novel natural inhibitors of the reverse transcriptase of human immunodeficiency virus from petrosia sp. *Tetrahedron* **1993**, *49*, 10435-10438.
48. Isaacs, S.; Hizi, A.; Kashman, Y. Toxicols a-c and toxiusol - new bioactive hexaprenoid hydroquinones from toxiclona toxius. *Tetrahedron* **1993**, *49*, 4275-4282.
49. Bourguet-Kondracki, M.-L.; Guyot, M. A new sesquiterpene tetronic acid derivative from the marine sponge smenospongia sp. *Tetrahedron Letters* **1999**, *40*, 3149-3150.
50. Rudi, A.; Stein, Z.; Goldberg, I.; Yosief, T.; Kashman, Y.; Schleyer, M. Yardenone and abudinol two new triterpenes from the marine sponge ptilocaulis spiculifer. *Tetrahedron Letters* **1998**, *39*, 1445-1448.
51. Rudi, A.; Kashman, Y. Aaptosine - a new cytotoxic 5,8-diazabenz[cd]azulene alkaloid from the red sea sponge aaptos aaptos. *Tetrahedron Letters* **1993**, *34*, 4683-4684.
52. Youssef, D.T.A.; Yamaki, R.K.; Kelly, M.; Scheuer, P.J. Salmahyrtisol a, a novel cytotoxic sesterterpene from the red sea sponge hyrtios erecta. *Journal of Natural Products* **2002**, *65*, 2-6.

53. Youssef, D.T.A.; Yoshida, W.Y.; Kelly, M.; Scheuer, P.J. Cytotoxic cyclic norterpene peroxides from a red sea sponge diacarnus erythraenus. *Journal of Natural Products* **2001**, *64*, 1332-1335.
54. Lefranc, F.; Nuzzo, G.; Hamdy, N.A.; Fakhr, I.; Moreno, Y.B.L.; Van Goietsenoven, G.; Villani, G.; Mathieu, V.; van Soest, R.; Kiss, R.*, et al.* In vitro pharmacological and toxicological effects of norterpene peroxides isolated from the red sea sponge diacarnus erythraeanus on normal and cancer cells. *J Nat Prod* **2013**, *76*, 1541-1547.
55. Youssef, D.T.A. Tasnemoxides a−c, new cytotoxic cyclic norsesterterpene peroxides from the red sea sponge diacarnus erythraenus. *Journal of Natural Products* **2004**, *67*, 112-114.
56. Abou-Hussein, D.R.; Badr, J.M.; Youssef, D.T. Dragmacidoside: A new nucleoside from the red sea sponge dragmacidon coccinea. *Nat Prod Res* **2014**, *28*, 1134-1141.
57. Shaala, L.A.; Youssef, D.T.; Ibrahim, S.R.; Mohamed, G.A. Callyptide a, a new cytotoxic peptide from the red sea marine sponge callyspongia species. *Nat Prod Res* **2016**, 1-8.
58. Shaaban, M.; Abd-Alla, H.I.; Hassan, A.Z.; Aly, H.F.; Ghani, M.A. Chemical characterization, antioxidant and inhibitory effects of some marine sponges against carbohydrate metabolizing enzymes. *Org Med Chem Lett* **2012**, *2*, 30.
59. Mohamed, G.A.; Abd-Elrazek, A.E.; Hassanean, H.A.; Alahdal, A.M.; Almohammadi, A.; Youssef, D.T. New fatty acids from the red sea sponge mycale euplectellioides. *Nat Prod Res* **2014**, *28*, 1082-1090.
60. Abdelmohsen, U.R.; Cheng, C.; Reimer, A.; Kozjak-Pavlovic, V.; Ibrahim, A.K.; Rudel, T.; Hentschel, U.; Edrada-Ebel, R.; Ahmed, S.A. Antichlamydial sterol from the red sea sponge callyspongia aff. Implexa. *Planta Med* **2015**, *81*, 382-387.
61. Guo, Y.; Gavagnin, M.; Mollo, E.; Cimino, G.; Hamdy, N.A.; Fakhr, I.; Pansini, M. Hurghamides a-d, new n-acyl-2-methylene-β-alanine methyl esters from red sea hippospongia sp. *Natural Product Letters* **1997**, *10*, 143-150.
62. Angawi, R.F.; Saqer, E.; Abdel-Lateff, A.; Badria, F.A.; Ayyad, S.E. Cytotoxic neviotane triterpene-type from the red sea sponge siphonochalina siphonella. *Pharmacogn Mag* **2014**, *10*, S334-341.
63. Abdelhameed, R.; Elgawish, M.S.; Mira, A.; Ibrahim, A.K.; Ahmed, S.A.; Shimizu, K.; Yamada, K. Anti-choline esterase activity of ceramides from the red sea marine sponge mycale euplectellioides. *RSC Advances* **2016**, *6*, 20422-20430.
64. Youssef, D.T.A.; Singab, A.N.B.; van Soest, R.W.M.; Fusetani, N. Hyrtiosenolides a and b, two new sesquiterpene γ-methoxybutenolides and a new sterol from a red sea sponge hyrtios species. *Journal of Natural Products* **2004**, *67*, 1736-1739.
65. Orabi, K.Y.; El Sayed, K.A.; Hamann, M.T.; Dunbar, D.C.; Al-Said, M.S.; Higa, T.; Kelly, M. Araguspongines k and l, new bioactive bis-1-oxaquinolizidine n-oxide alkaloids from red sea specimens of xestospongia exigua. *Journal of Natural Products* **2002**, *65*, 1782-1785.
66. Youssef, D.T. Hyrtioerectines a-c, cytotoxic alkaloids from the red sea sponge hyrtioserectus. *J Nat Prod* **2005**, *68*, 1416-1419.
67. Youssef, D.T.A.; Shaala, L.A.; Emara, S. Antimycobacterial scalarane-based sesterterpenes from the red sea sponge hyrtios erecta. *Journal of Natural Products* **2005**, *68*, 1782-1784.
68. Sauleau, P.; Martin, M.T.; Dau, M.E.; Youssef, D.T.; Bourguet-Kondracki, M.L. Hyrtiazepine, an azepino-indole-type alkaloid from the red sea marine sponge hyrtios erectus. *J Nat Prod* **2006**, *69*, 1676-1679.
69. Yosief, T.; Rudi, A.; Wolde-ab, Y.; Kashman, Y. Two new c22 1,2-dioxane polyketides from the marine sponge acarnus cf. Bergquistae. *Journal of Natural Products* **1998**, *61*, 491-493.
70. Meyer, M.; Guyot, M. New sphingosines from the marine sponge grayella cyatophora. *J Nat Prod* **2002**, *65*, 1722-1723.
71. Chill, L.; Yosief, T.; Kashman, Y. Halichondramine, a new tetracyclic bipiperidine alkaloid from the marine sponge halichondria sp. *J Nat Prod* **2002**, *65*, 1738-1741.
72. Yosief, T.; Rudi, A.; Kashman, Y. Asmarines a-f, novel cytotoxic compounds from the marine sponge raspailia species. *J Nat Prod* **2000**, *63*, 299-304.

73. Rudi, A.; Yosief, T.; Loya, S.; Hizi, A.; Schleyer, M.; Kashman, Y. Clathsterol, a novel anti-hiv-1 rt sulfated sterol from the sponge clathria species. *J Nat Prod* **2001**, *64*, 1451-1453.
74. Chill, L.; Miroz, A.; Kashman, Y. Haliclonyne, a new highly oxygenated polyacetylene from the marine sponge haliclona species. *Journal of Natural Products* **2000**, *63*, 523-526.
75. Jain, S.; Laphookhieo, S.; Shi, Z.; Fu, L.W.; Akiyama, S.; Chen, Z.S.; Youssef, D.T.; van Soest, R.W.; El Sayed, K.A. Reversal of p-glycoprotein-mediated multidrug resistance by sipholane triterpenoids. *J Nat Prod* **2007**, *70*, 928-931.
76. Jain, S.; Abraham, I.; Carvalho, P.; Kuang, Y.H.; Shaala, L.A.; Youssef, D.T.; Avery, M.A.; Chen, Z.S.; El Sayed, K.A. Sipholane triterpenoids: Chemistry, reversal of abcb1/p-glycoprotein-mediated multidrug resistance, and pharmacophore modeling. *J Nat Prod* **2009**, *72*, 1291-1298.
77. Isaacs, S.; Kashman, Y. Shaagrockol b and c; two hexaprenylhydroquinone disulfates from the red sea sponge toxiclona toxius. *Tetrahedron Letters* **1992**, *33*, 2227-2230.
78. Loya, S.; Tal, R.; Hizi, A.; Issacs, S.; Kashman, Y.; Loya, Y. Hexaprenoid hydroquinones, novel inhibitors of the reverse transcriptase of human immunodeficiency virus type 1. *J Nat Prod* **1993**, *56*, 2120-2125.
79. Vilozny, B.; Amagata, T.; Mooberry, S.L.; Crews, P. A new dimension to the biosynthetic products isolated from the sponge negombata magnifica. *J Nat Prod* **2004**, *67*, 1055-1057.
80. El Sayed, K.A.; Youssef, D.T.; Marchetti, D. Bioactive natural and semisynthetic latrunculins. *J Nat Prod* **2006**, *69*, 219-223.
81. Guo, Y.; Gavagnin, M.; Mollo, E.; Trivellone, E.; Cimino, G.; Hamdy, N.A.; Fakhr, I.; Pansini, M. A new norsesterterpene peroxide from a red sea sponge. *Natural Product Letters* **1996**, *9*, 105-112.
82. Hamed, A.N.E.; Schmitz, R.; Bergermann, A.; Totzke, F.; Kubbutat, M.; Muller, W.E.G.; Youssef, D.T.A.; Bishr, M.M.; Kamel, M.S.; Edrada-Ebel, R.*, et al.* Bioactive pyrrole alkaloids isolated from the red sea: Marine sponge stylissa carteri. *Z Naturforsch C* **2018**, *73*, 199-210.
83. El-Beih, A.A.; El-Desoky, A.H.; Al-hammady, M.A.; Elshamy, A.I.; Hegazy, M.-E.F.; Kato, H.; Tsukamoto, S. New inhibitors of rankl-induced osteoclastogenesis from the marine sponge siphonochalina siphonella. *Fitoterapia* **2018**, *128*, 43-49.
84. Kashman, Y.; Yosief, T.; Carmeli, S. New triterpenoids from the red sea sponge siphonochalina siphonella. *Journal of Natural Products* **2001**, *64*, 175-180.
85. Shaala, L.A.; Youssef, D.T.A. Cytotoxic psammaplysin analogues from the verongid red sea sponge aplysinella species. *Biomolecules* **2019**, *9*.
86. El-Hawary, S.S.; Sayed, A.M.; Mohammed, R.; Hassan, H.M.; Rateb, M.E.; Amin, E.; Mohammed, T.A.; El-Mesery, M.; Bin Muhsinah, A.; Alsayari, A.*, et al.* Bioactive brominated oxindole alkaloids from the red sea sponge callyspongia siphonella. *Marine drugs* **2019**, *17*, 465.
87. Shaaban, M.; Shaaban, K.A.; Ghani, M.A. Hurgadacin: A new steroid from sinularia polydactyla. *Steroids* **2013**, *78*, 866-873.
88. Abou El-Ezz, R.F.; Ahmed, S.A.; Radwan, M.M.; Ayoub, N.A.; Afifi, M.S.; Ross, S.A.; Szymanski, P.T.; Fahmy, H.; Khalifa, S.I. Bioactive cembranoids from the red sea soft coral sarcophyton glaucum. *Tetrahedron Letters* **2013**, *54*, 989-992.
89. Eltahawy, N.A.; Ibrahim, A.K.; Radwan, M.M.; ElSohly, M.A.; Hassanean, H.A.; Ahmed, S.A. Cytotoxic cembranoids from the red sea soft coral, sarcophyton auritum. *Tetrahedron Letters* **2014**, *55*, 3984-3988.
90. Hegazy, M.E.; Mohamed, T.A.; Elshamy, A.I.; Al-Hammady, M.A.; Ohta, S.; Pare, P.W. Casbane diterpenes from red sea coral sinularia polydactyla. *Molecules* **2016**, *21*, 308.
91. Al-Lihaibi, S.S.; Alarif, W.M.; Abdel-Lateff, A.; Ayyad, S.E.; Abdel-Naim, A.B.; El-Senduny, F.F.; Badria, F.A. Three new cembranoid-type diterpenes from red sea soft coral sarcophyton glaucum: Isolation and antiproliferative activity against hepg2 cells. *Eur J Med Chem* **2014**, *81*, 314-322.
92. Shaaban, M.; Ghani Mohamed, A.; Shaaban Khaled, A. Zahramycins a-b, two new steroids from the coral sarcophyton trocheliophorum. In *Zeitschrift für Naturforschung B*, 2013; Vol. 68, p 939.

93. Abdel-Lateff, A.; Alarif, W.M.; Ayyad, S.E.; Al-Lihaibi, S.S.; Basaif, S.A. New cytotoxic isoprenoid derivatives from the red sea soft coral sarcophyton glaucum. *Nat Prod Res* **2015**, *29*, 24-30.
94. Temraz, T.A.; Houssen, W.E.; Jaspars, M.; Woolley, D.R.; Wease, K.N.; Davies, S.N.; Scott, R.H. A pyridinium derivative from red sea soft corals inhibited voltage-activated potassium conductances and increased excitability of rat cultured sensory neurones. *BMC Pharmacol* **2006**, *6*, 10.
95. Hegazy, M.-E.F.; Mohamed, T.A.; Abdel-Latif, F.F.; Alsaid, M.S.; Shahat, A.A.; Paré, P.W. Trochelioid a and b, new cembranoid diterpenes from the red sea soft coral sarcophyton trocheliophorum. *Phytochemistry Letters* **2013**, *6*, 383-386.
96. Gomaa, M.N.; Soliman, K.; Ayesh, A.; Abd El-Wahed, A.; Hamza, Z.; Mansour, H.M.; Khalifa, S.A.; Mohd Ali, H.B.; El-Seedi, H.R. Antibacterial effect of the red sea soft coral sarcophyton trocheliophorum. *Nat Prod Res* **2016**, *30*, 729-734.
97. Elbagory, A.M.; Meyer, M.; Ali, A.H.; Ameer, F.; Parker-Nance, S.; Benito, M.T.; Doyaguez, E.G.; Jimeno, M.L.; Hussein, A.A. New polyhydroxylated sterols from palythoa tuberculosa and their apoptotic activity in cancer cells. *Steroids* **2015**, *101*, 110-115.
98. Groweiss, A.; Kashman, Y. Eight new xenia diterpenoids from three soft corals of the red sea. *Tetrahedron* **1983**, *39*, 3385-3396.
99. Hassan, H.M.; Khanfar, M.A.; Elnagar, A.Y.; Mohammed, R.; Shaala, L.A.; Youssef, D.T.; Hifnawy, M.S.; El Sayed, K.A. Pachycladins a-e, prostate cancer invasion and migration inhibitory eunicellin-based diterpenoids from the red sea soft coral cladiella pachyclados. *J Nat Prod* **2010**, *73*, 848-853.
100. Ellithey, M.S.; Lall, N.; Hussein, A.A.; Meyer, D. Cytotoxic, cytostatic and hiv-1 pr inhibitory activities of the soft coral litophyton arboreum. *Mar Drugs* **2013**, *11*, 4917-4936.
101. Hegazy, M.E.; Gamal Eldeen, A.M.; Shahat, A.A.; Abdel-Latif, F.F.; Mohamed, T.A.; Whittlesey, B.R.; Pare, P.W. Bioactive hydroperoxyl cembranoids from the red sea soft coral sarcophyton glaucum. *Mar Drugs* **2012**, *10*, 209-222.
102. Al-Lihaibi, S.S.; Ayyad, S.E.; Shaher, F.; Alarif, W.M. Antibacterial sphingolipid and steroids from the black coral antipathes dichotoma. *Chem Pharm Bull (Tokyo)* **2010**, *58*, 1635-1638.
103. Řezanka, T.; Dembitsky, V.M. Γ-lactones from the soft corals sarcophyton trocheliophorum and lithophyton arboreum. *Tetrahedron* **2001**, *57*, 8743-8749.
104. Green, D.; Kashman, Y.; Benayahu, Y. Secondary metabolites of the yellow and gray morphs of the soft coral parerythropodium fulvum fulvum: Comparative aspects. *Journal of Natural Products* **1992**, *55*, 1186-1196.
105. Kashman, Y.; Bodner, M.; Loya, Y.; Benayahu, Y. Cembranolids from marine origin (red sea), survey, and isolation of a new sinulariolide derivative. *Israel Journal of Chemistry* **1977**, *16*, 1-3.
106. el Sayed, K.A.; Hamann, M.T. A new norcembranoid dimer from the red sea soft coral sinularia gardineri. *J Nat Prod* **1996**, *59*, 687-689.
107. Hassan, H.M.; Rateb, M.E.; Hassan, M.H.; Sayed, A.M.; Shabana, S.; Raslan, M.; Amin, E.; Behery, F.A.; Ahmed, O.M.; Bin Muhsinah, A.*, et al.* New antiproliferative cembrane diterpenes from the red sea sarcophyton species. *Marine drugs* **2019**, *17*, 411.
108. Ayyad, S.N.; Deyab, M.A.; Kosbar, T.; Alarif, W.M.; Eissa, A.H. Bio-active sesquiterpenoids and norsesquiternoids from the red sea octocoral rhytisma fulvum fulvum. *Nat Prod Res* **2019**, 1-8.
109. Řezanka, T.; Hanuš, L.O.; Dembitsky, V.M. Lytophilippines a–c: Novel macrolactones from the red sea hydroid lytocarpus philippinus. *Tetrahedron* **2004**, *60*, 12191-12199.
110. Guo, Y.; Gavagnin, M.; Mollo, E.; Trivellone, E.; Cimino, G.; Fakhr, I. Structure of the pigment of the red sea nudibranch hexabranchus sanguineus. *Tetrahedron Letters* **1998**, *39*, 2635-2638.
111. Ahmed, H.H.; Hegazi, M.M.; Abd-Alla, H.I.; Eskander, E.F.; Ellithey, M.S. Antitumour and antioxidant activity of some red sea seaweeds in ehrlich ascites carcinoma in vivo. *Z Naturforsch C* **2011**, *66*, 367-376.
112. Mohamed, G.A.; Ibrahim, S.R.M.; Badr, J.M.; Youssef, D.T.A. Didemnaketals d and e, bioactive terpenoids from a red sea ascidian didemnum species. *Tetrahedron* **2014**, *70*, 35-40.

113. Hamdy, A.H.; Mettwally, W.S.; El Fotouh, M.A.; Rodriguez, B.; El-Dewany, A.I.; El-Toumy, S.A.; Hussein, A.A. Bioactive phenolic compounds from the egyptian red sea seagrass thalassodendron ciliatum. *Z Naturforsch C* **2012**, *67*, 291-296.
114. Ibrahim, A.K.; Youssef, A.I.; Arafa, A.S.; Foad, R.; Radwan, M.M.; Ross, S.; Hassanean, H.A.; Ahmed, S.A. Anti-h5n1 virus new diglyceride ester from the red sea grass thallasodendron ciliatum. *Nat Prod Res* **2013**, *27*, 1625-1632.
115. Mohammed, M.M.; Hamdy, A.H.; El-Fiky, N.M.; Mettwally, W.S.; El-Beih, A.A.; Kobayashi, N. Anti-influenza a virus activity of a new dihydrochalcone diglycoside isolated from the egyptian seagrass thalassodendron ciliatum (forsk.) den hartog. *Nat Prod Res* **2014**, *28*, 377-382.
116. Ayyad, S.E.; Al-Footy, K.O.; Alarif, W.M.; Sobahi, T.R.; Bassaif, S.A.; Makki, M.S.; Asiri, A.M.; Al Halwani, A.Y.; Badria, A.F.; Badria, F.A. Bioactive c15 acetogenins from the red alga laurencia obtusa. *Chem Pharm Bull (Tokyo)* **2011**, *59*, 1294-1298.
117. Green, D.; Kashman, Y.; Miroz, A. Colpol, a new cytotoxic c6-c4-c6 metabolite from the alga colpomenia sinuosa. *Journal of Natural Products* **1993**, *56*, 1201-1202.
118. Ayyad, S.E.; Sowellim, S.Z.; el-Hosini, M.S.; Abo-Atia, A. The structural determination of a new steroidal metabolite from the brown alga sargassum asperifolium. *Z Naturforsch C* **2003**, *58*, 333-336.
119. Gedara, S.R.; Abdel-Halim, O.B.; el-Sharkawy, S.H.; Salama, O.M.; Shier, T.W.; Halim, A.F. Cytotoxic hydroazulene diterpenes from the brown alga dictyota dichotoma. *Z Naturforsch C* **2003**, *58*, 17-22.
120. Alarif, W.M.; Ayyad, S.E.; El-Assouli, S.M.; Al-Lihaibi, S.S. Antigenotoxic ketosteroid from the red algae jania adhaerens. *Nat Prod Res* **2012**, *26*, 785-791.
121. Abou-El-Wafa, G.S.; Shaaban, M.; Shaaban, K.A.; El-Naggar, M.E.; Maier, A.; Fiebig, H.H.; Laatsch, H. Pachydictyols b and c: New diterpenes from dictyota dichotoma hudson. *Mar Drugs* **2013**, *11*, 3109-3123.
122. Alarif, W.M.; Al-Lihaibi, S.S.; Ayyad, S.E.; Abdel-Rhman, M.H.; Badria, F.A. Laurene-type sesquiterpenes from the red sea red alga laurencia obtusa as potential antitumor-antimicrobial agents. *Eur J Med Chem* **2012**, *55*, 462-466.
123. Pereira, H.; Custodio, L.; Rodrigues, M.J.; de Sousa, C.B.; Oliveira, M.; Barreira, L.; Neng Nda, R.; Nogueira, J.M.; Alrokayan, S.A.; Mouffouk, F.*, et al.* Biological activities and chemical composition of methanolic extracts of selected autochthonous microalgae strains from the red sea. *Mar Drugs* **2015**, *13*, 3531-3549.
124. Ayyad, S.-E.N.; Dawidar, A.-A.M.; Dias, H.W.; Howie, R.A.; Jakupovic, J.; Thomson, R.H. Three halogenated metabolites fromlaurencia obtusa. *Phytochemistry* **1990**, *29*, 3193-3196.
125. Alarif, W.M.; Al-Lihaibi, S.S.; Bawakid, N.O.; Abdel-Lateff, A.; Al-Malky, H.S. Rare acetogenins with anti-inflammatory effect from the red alga laurencia obtusa. *Molecules* **2019**, *24*.
126. Khattab, R.A.; Elbandy, M.; Lawrence, A.; Paget, T.; Rae-Rho, J.; Binnaser, Y.S.; Ali, I. Extraction, identification and biological activities of saponins in sea cucumber pearsonothuria graeffei. *Comb Chem High Throughput Screen* **2018**, *21*, 222-231.
